# Cisplatin and Starvation Differently Sensitize Autophagy in Renal Carcinoma: A Potential Therapeutic Pathway to Target Variegated Drugs Resistant Cancerous Cells

**DOI:** 10.3390/cells13060471

**Published:** 2024-03-07

**Authors:** Ankita Dutta, Subarna Thakur, Debasish Kumar Dey, Anoop Kumar

**Affiliations:** 1 Advanced Nanoscale Molecular Oncology Laboratory (ANMOL), Department of Biotechnology, University of North Bengal, Siliguri 734013, West Bengal, India; 2 Department of Bioinformatics, University of North Bengal, Siliguri 734013, West Bengal, India; 3 Stephenson Cancer Center, University of Oklahoma Health Sciences Center, Oklahoma City, OK 73104, USA

**Keywords:** cisplatin, autophagy, starvation, transcriptome, differentially expressed genes (DEGs), pathway enrichment analysis, hub genes, qRT-PCR, MTT assay, apoptosis

## Abstract

Cisplatin, a powerful chemotherapy medication, has long been a cornerstone in the fight against cancer due to chemotherapeutic failure. The mechanism of cisplatin resistance/failure is a multifaceted and complex issue that consists mainly of apoptosis inhibition through autophagy sensitization. Currently, researchers are exploring ways to regulate autophagy in order to tip the balance in favor of effective chemotherapy. Based on this notion, the current study primarily identifies the differentially expressed genes (DEGs) in cisplatin-treated autophagic ACHN cells through the Illumina Hi-seq platform. A protein–protein interaction network was constructed using the STRING database and KEGG. GO classifiers were implicated to identify genes and their participating biological pathways. ClueGO, David, and MCODE detected ontological enrichment and sub-networking. The network topology was further examined using 12 different algorithms to identify top-ranked hub genes through the Cytoscape plugin Cytohubba to identify potential targets, which established profound drug efficacy under an autophagic environment. Considerable upregulation of genes related to autophagy and apoptosis suggests that autophagy boosts cisplatin efficacy in malignant ACHN cells with minimal harm to normal HEK-293 growth. Furthermore, the determination of cellular viability and apoptosis by AnnexinV/FITC-PI assay corroborates with in silico data, indicating the reliability of the bioinformatics method followed by qRT-PCR. Altogether, our data provide a clear molecular insight into drug efficacy under starved conditions to improve chemotherapy and will likely prompt more clinical trials on this aspect.

## 1. Introduction

Resistance developed against standard chemotherapeutic drugs emerges as a major challenge, where patients with advanced-stage solid tumors only exhibit transient response to targeted therapy [1]. In advanced-stage solid cancer patients, popular targeted therapies such as kinase inhibitors against *ALK* (anaplastic lymphoma kinase), *EGFR* (epidermal growth factor receptor), *RET* (ret proto-oncogene), *ROS1* (ROS proto-oncogene 1), and *MEK* (mitogen-activated protein kinase 1) lead to the progression of a therapy-resistant tumor after an inadequate therapeutic response [1]. Therefore, efficient targeted therapy, which enables the application of chemotherapeutic drugs either alone or in combination, is now widely being investigated to overcome chemo-resistance.

Caloric modulation (fasting) holds a vast potential to support conventional chemotherapy in many ways involving systemic alteration in hormones (glucagon, insulin, glucocorticoids), metabolites (amino acids, glucose, ketone bodies), and growth factors [2]. Due to their extended high proliferation rates, cancer cells are unable to adapt to a fasting environment, unlike normal cells [3], and as a consequence, different cells exhibit differential susceptibilities toward nutrient deprivation [4,5]. Normal cells refocus their energy on cellular repair and maintenance activities rather than proliferation and growth and thus are protected from unpleasant chemo-toxicity [5]. The literature suggests that short-term fasting induces autophagy, a physiologically conserved mechanism that recycles unutilized or damaged cellular components to produce ATP for cell survival [5,6]. However, autophagy activation/suppression plays a dicey role during chemotherapy [6,7].

Cisplatin (cis-diaminedichloroplatinum) is the first line of chemotherapeutic defense used to control fatalities caused by testicular, ovarian, and even kidney cancer [8,9,10], but its application is limited due to chemotherapeutic resistance caused by decreased drug uptake, increased efflux, and altered expression of autophagy-associated genes [11]. The use of cisplatin has also been limited due to reports of nephrotoxicity from long-term medication exposure [12]. To combat this, a number of targeted therapies, including the use of plant alkaloids, miRNA, or autophagy modulation, are currently employed widely to increase the chemo-sensitivity of cancer cells [9,10]. But efficient therapeutic strategies are still limited in this context. Notably, no direct evidence has been reported yet that delineates the exact role of autophagy in tumor progression or suppression. Here, for the first time, we conceptualize using short-term fasting-induced autophagy to control (ACHN) renal carcinoma growth and counteract the previously reported nephrotoxic effect on normal HEK-293 cells. The following treatment not only controls cancer cell proliferation but also minimizes its cytotoxic potential against HEK-293, possibly through normal cells’ differential stress responses followed by immunomodulatory effects [2,13].

To date, there is no bioinformatics-based evidence reported that supports increasing chemo-sensitivity of kidney carcinoma cells toward cisplatin under an autophagic environment. In this study, by using multiple bioinformatics approaches, we identified major regulatory genes and subsequent pathways controlling autophagy-mediated drug toxicity. The differential gene expression (DEG) profiling obtained from Illumina Hiseq transcriptomic data enables us to consider all GO (gene ontology) and KEGG (Kyoto Encyclopedia of Genes and Genomes) database searches to identify potential gene expression under the experimental parameters. Furthermore, the PPI (protein–protein interaction) network generated by STRING (Search Tool for the Retrieval of Interacting Genes/Proteins, version 11.5; https://string-db.org, accessed on 1 September 2023) also enables us to target major hub genes through the Cytoscape plugin Cytohubba, ver. 1.5.1. Finally, cellular viability and apoptosis data also corroborate in silico results that suggest inhibition of cancer cells under autophagy and cisplatin medication. Thus, our findings particularly delineate the major biological mechanism and identify potential bio-markers to increase chemotherapeutic efficacy during renal carcinoma under short-term fasting.

## 2. Materials and Methods

### 2.1. Cell Lines

ACHN (human kidney carcinoma) and HEK-293 (normal human embryonic kidney) cell lines were procured from National Centre for Cell Science (NCCS), Pune (India), and were grown in DMEM-F12 (Dulbecco’s modified Eagle medium; Hi-media Laboratory Pvt. Ltd., India; AT127-20L) media with 10% FCS (fetal calf serum; Hi-media Laboratory Pvt. Ltd., India; RM10437-500mL) at 37 °C in humidified atmosphere containing 5% CO_2_. The growth medium was supplemented with 10 U/mL of penicillin G and 10 U/mL of streptomycin (Sigma-Aldrich, USA; A004-5X50ML). When cells were at an appropriate confluency (80–90%), they were passaged by trypsinizing with 1X Trypsin-EDTA (Hi-media Laboratory Pvt. Ltd., India; TCL070-100mL). By substituting culture media with PBS, complete nutritional deprivation was achieved (phosphate-buffered saline; Hi-media Laboratory Pvt. Ltd., India; ML023).

### 2.2. Antibodies and Chemicals

Antibodies used include Beclin-1 rabbit mAb 3495 (Cell Signalling Technology, USA; D4065), LC3A/BXP^®^ Rabbit mAb #12741 (Cell Signalling Technology, USA; D3U4C), Atg5 rabbit mAb #12994 (Cell Signalling Technology, USA; D5F5U), Atg12 rabbit mAb #4180 (Cell Signalling Technology, USA; D88H11), Atg16L1 rabbit mAb #8089 (Cell Signalling Technology, USA; D6D5), Atg7 rabbit mAb #8558 (Cell Signalling Technology, USA; D12B11), Atg3 rabbit mAb #3415 (Cell Signalling Technology, USA), and Anti-rabbit IgG, HRP-linked Antibody #7074. ß-actin was taken as a housekeeping gene (Santa Cruz Biotechnology, USA; SC 47778). The CYTO-ID^®^ autophagy detection kit was obtained from Enzo Life Sciences, USA. The kit (ENZ-51031-0050) included rapamycin (500 mM), which was used as a positive inducer of autophagy. Cisplatin (Sigma-Aldrich, USA; CAS 15663-27-1) was dissolved in water to prepare 1 mM stock solution. RevertAid First-Strand cDNA Synthesis Kit (Thermo Scientific, USA) was used for first-strand cDNA synthesis. TRI reagent, annexin V-FITC apoptosis kit (APOAF-20TST), and MTT [3-(4,5-dimethylthiazol-2-yl)-2,5-diphenyltetrazolium bromide)] were purchased (m5655-100mg) from Sigma-Aldrich, USA.

### 2.3. Autophagy Detection after Complete Nutrient Deprivation via Qualitative and Quantitative Approaches

HEK-293 and ACHN cell lines were cultured in sterile coverslips placed in 35 mm Petri dishes at a 37 °C temperature with 5% CO_2_. After 24 h, cell culture media were replaced with PBS and kept for at least 3 h to detect autophagy induction [14]. Rapamycin was taken as a positive control (5 µM). Visualization was performed under 40× magnification of a fluorescence microscope (Magnus MLXi, India) after staining the cells with CYTO-ID^®^ autophagy detection kit, according to the manufacturer’s protocol [15].

After successful autophagy detection, both treated and untreated cells were subjected to quantitative expression profiling of autophagy-related antibodies by indirect ELISA [16] method. Total protein was isolated and quantified by Bradford’s technique [17]. Quantitative estimation of major autophagy-related biomarkers (1:100 dilution was used for each antibody) was recorded at 450 nm wavelength by a SPECTROstar Nano plate reader (BMG Labtech, Germany) [16].

### 2.4. Cell Viability Determination by MTT Assay and Trypan Blue Exclusion Assay

To determine the cellular reproducibility deduction by cisplatin, an MTT assay [18] was performed. Briefly, cells were incubated with increasing concentrations (10–100 μM) of cisplatin drug at 37 °C in a 96-well culture plate. After 24 h, culture media were replaced with 5 mg/mL of MTT solution and kept for an additional 3 h. Thereafter, isopropanol was added to dissolve formazan produced by living cells. At 620 nm, absorbance was recorded with a microplate reader (SPECTROstar Nano, BMG Labtech, Germany). Cellular viability was calculated as the percentage of cell viability = [100 − {(A − B)/ × 100}], where “A” is the mean optical density of cells without any drug, and “B” is the mean optical density of cells treated with the drug in each mentioned concentration. A 50% inhibitory concentration (IC_50_) of cisplatin was determined separately for each cell line. Following that, trypan blue exclusion experiment was performed [19] to determine the viable and non-viable cells under cisplatin treatment. For that, The IC_50_ dose of 50 μM was applied for HEK-293, and 30 μM doses were applied in the case of ACHNs.

### 2.5. Preparation of Cells for RNA Isolation and High-Throughput Illumina Sequencing

Under an autophagic environment, transcriptomic analysis was performed to identify any altered gene expression that differentially increased cisplatin cytotoxicity. ACHN autophagic cell line (here, considered as control) and autophagic carcinoma cells treated with cisplatin (considered as treated) were further subjected to streaming experiments. For that, the conventional TRIzol (Sigma-Aldrich, USA) RNA extraction method was followed with some minor modifications [20]. 1% denaturing agarose gel electrophoresis and NanoDrop spectrophotometer (SPECTROstar Nano BMG Labtech, Germany) were used for qualitative and quantitative evaluation of extracted RNA samples (Table S1). Total RNA from three replicates of each sample was used for library processing and paired-end sequencing [21]. According to the TruSeq standard total RNA reference guide (Illumina Technologies, San Diego, CA, USA), library preparation was carried out using the Illumina HiSeq 4000 platform (Illumina Inc., CA, USA), which was subjected to automated cycles of paired-end sequencing (2 × 150 bp chemistry).

### 2.6. RNA Sequencing Data Pre-Processing, De Novo Transcriptome Assembly, and Analysis of Differential Gene Expression

Adapter removal (version 2.2.0) was applied to remove adapter sequences of Illumina raw reads. From the paired-end reads, reads with an average quality score of <20 were filtered away. With the help of Trimmomatic v0.38, low-quality reads were trimmed. Homosapien genome assembly GRCh38 was used as a reference to align the trimmed reads. Transcriptomes were assembled by NFCore RNA Seq pipeline followed by R package EdgeR (version 3.6) for differential gene expression analysis. Using KAAS (KEGG Automatic Annotation Server, an integrated database made up of various curated databases that offers a higher level of comprehension of systemic functions), functional annotations of all the genes were performed [22]. Manual transcriptomic data mining was accomplished to identify differentially upregulated and downregulated genes and interpreted through TBtools (https://github.com/CJ-Chen/TBtools/releases) to generate the heat maps with FPKM (fragments per kilobase of transcript per million mapped reads) values [23]. SRPLOT online tool (http://www.bioinformatics.com.cn/srplot (accessed on 11 September 2023)) was used to generate cneplot, emamplot, enrichment score, and bubble plot of all expressed genes with a cut-off value of log2Fc > 2 and *p* < 0.05 [24].

### 2.7. Functional Enrichment Analysis of Identified Differentially Expressed Genes

A web-based database STRING (Search Tool for the Retrieval of Interacting Genes, version 11.5; https://string-db.org) was implicated in generating a protein–protein interaction (PPI) network for functional enrichment analysis of differentially expressed genes [25]. To identify the top-ranked biological process (BP), cellular component (CC) and molecular function (MF) generated by STRING were aligned with respect to percentile ranking of the acquired GO (gene ontology) score by ClueGO (v3.9.1). A combination score cut off >0.4 was chosen for the differentially upregulated genes. A score of >0.4 denotes a moderate level of confidence. In order to avoid false positives, interactions below 0.4 were not taken into consideration. Thereafter, Cytoscape v3.9.1 was used to display the PPI network [26]. In order to extract the highly interconnected regions (cluster) based on topology, the Cytoscape plugin MCODE (molecular complex detection) was used (version 2.0.0) considering the following parameters: degree cut-off = 2 (nodes with a single link have been disregarded since the degree cut-off specifies the minimum number of connections that a node must possess to be eligible for cluster membership); node score cut-off = 0.2 (using connections and other parameters, this score evaluates a node’s significance within the network; nodes with less than 0.2 scores were not considered); node density cut-off = 0.1 (the number of connections is the bare minimum score needed for a cluster to be categorized as a “dense” region; when selecting a 0.1 node density cut-off, only clusters with at least 10% of their potential connections present are taken into consideration); K-core = 2 (it is the measure of a node’s connectivity which depends on the number of connections/associations it has with other high-degree nodes; with a K-core of 2, only nodes that are part of a subgraph with at least two edges were taken into consideration for clustering); and max depth = 100 (in a maximum of these stages, MCODE will expand a cluster from its seed nodes; if a cluster’s maximum depth is set to 100, it will continue to expand until it is 100 nodes away from its seed nodes) [27]. The ClueGO plugin v3.9.1 was used to integrate all GO data into each cluster, and a separate analysis was performed to functionally annotate each of them [28].

### 2.8. Hub Gene Identification among the Upregulated DEGs

MCODE clusters were clubbed together to identify the hub genes governing biological pathways using the Cytoscape plugin cytoHubba v1.5.1. A total of 12 different algorithms, including stress, radiality, MNC, MCC, EcCentricity, BottleNeck, DEGREE, betweenness, EPC, Closeness, clustering coefficient, and DMNC, were employed to evaluate the process [29]. Finally, KEGG analysis was carried out once more to study the hub genes and reveal the precise pathway they are regulating.

### 2.9. Cross-Validation of Identified Hub Genes by Quantitative Real-Time PCR

To validate differentially regulated major hub genes, quantitative real-time PCR (qPCR) analysis was carried out using gene-specific primer sets (Table S2). Total RNA isolation was performed by TRIzol (Sigma-Aldrich, USA) method following first-strand cDNA synthesis using RevertAid First-Strand cDNA Synthesis Kit (Thermo Scientific, USA), according to manufacturer’s protocol. The primers for the gene expression studies were designed from the retrieved sequences using the Primer Quest^TM^ tool (https://eu.idtdna.com/PrimerQuest). Quantitative real-time PCR was executed on the synthesized first-strand cDNA using GoTaq qPCR Master Mix (Promega, Madison, WI, USA) in a total reaction volume of 20 μL containing 10 pmol primers and 50 ng cDNA template following the manufacturer’s instructions. Real-time PCR amplification was carried out in QuantStudio™ 3 System (Applied Biosystems, USA) using the SYBR Green Chemistry with three biological replicates. qRT PCR amplification was performed by pre-denaturation at 95 °C followed by 40 cycles of 15 s at 95 °C and 30 s at 62 °C, followed by 20 s at 95 °C, 60 s at 60 °C, and 15 s at 95 °C for dissociation curve generation [30]. In all experiments, the β-actin gene was used as endogenous control. After successful amplification, all the data were analyzed to determine the relative expression of the target gene and plotted graphically using GraphPad Prism v10.0.1 (GraphPad Software, San Diego, CA, USA).

### 2.10. Apoptosis Assay by AnnexinV/FITC-PI Staining

By using AnnexinV/FITC-PI staining method, cellular apoptosis was encountered under the experimental conditions [31]. Briefly, the cell pellet was isolated and suspended in 1× binding buffer, followed by staining with AnnexinV/FITC-PI dye for 10 min in the dark at room temperature. Fluorescence was recorded at 480/530 nm wavelength for annexin V/FITC, and 535/615 nm was applied for PI dye. Slides were visualized under 40× microscopic field (Magnus MLXi, India).

### 2.11. Statistical Analysis

All values are shown as the mean standard deviation (±SD) of three biological replicates. GraphPad Prism v10.0.1 (GraphPad Software, San Diego, CA, USA) was used to generate the graphs. One-way ANOVA was used to assess significance, and multiple comparisons between the control and treatment groups were compared using Tukey’s post hoc test. Differences with *p* < 0.05 were considered as significant. To assess each outcome, three independent experiments were conducted, and one representative data set was provided.

## 3. Results

### 3.1. Effect of Cisplatin under Starvation-Induced Autophagic Environment

**Figure 1:**
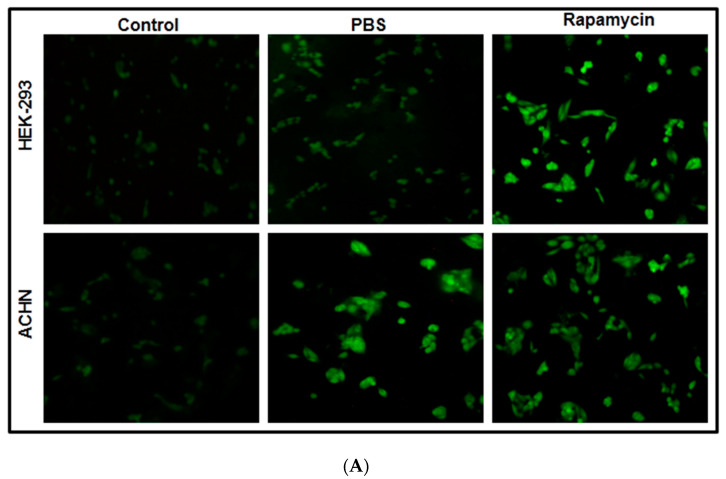

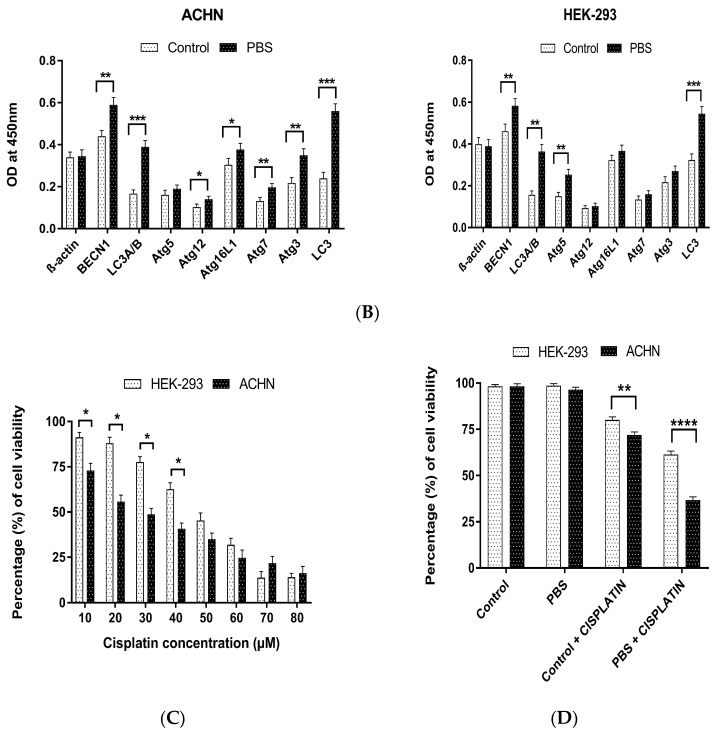
Effect of cisplatin in starvation-induced autophagic cell lines. (**A**) Autophagosomes were detected by CYTO-ID autophagy detection kit in nutrient-deficient normal (HEK-293) and cancer cell (ACHN) lines. Cells were treated in the presence of complete media (CM), PBS (autophagy inducer), and rapamycin (positive control) in both cell lines. (**B**) Antibody-based indirect ELISA was used to assess autophagy progression in control (cells without treatment) and in PBS-treated (nutrient-deprived for 3 h) cells. The cytosolic protein was extracted from each treatment condition from both cell lines, and quantification of autophagy-related biomarkers was achieved by recording absorbance at 450 nm using SPECTROStar Nano plate reader (BMG Labteck, Germany). (**C**) Percentage of cell viability after cisplatin treatment in HEK-293 and ACHN cell by MTT assay after 24 h. (**D**) Trypan blue exclusion assay for precise quantification of viable and non-viable cells in all conditions—control, PBS (without nutrient), control + cisplatin, and PBS + cisplatin (starvation-induced autophagic condition + cisplatin). All data are mean ± SD and are indicative of three separate studies. The significance level was set at *p* < 0.05 (*: *p* ≤ 0.05, **: *p* ≤ 0.01, ***: *p* ≤ 0.001, ****: *p* ≤ 0.0001), and the standard deviations of the data were displayed as error bars.

Anti-cancer drug efficacy has been proven to be influenced by autophagy-mediated cellular response [32,33]. In this study, we examined autophagy by complete nutrient deficiency (PBS treatment) in both normal (HEK-293) and cancerous (ACHN) kidney cell lines. The development of autophagic flux in PBS-treated cells was primarily examined through fluorescence microscopic observation (Figure 1A). In this instance, bright green fluorescence from starved cells indicated a positive autophagy progression, which is apparently absent on the control panel (in both cell lines). Rapamycin-treated cells (5 µM) were considered as positive control. The qualitative assay was further confirmed with quantitative expression profiling of autophagy-related bio-markers by indirect ELISA [16]. Experimental findings clearly demonstrated an elevated expression of autophagy-related biomarkers like LC3B, Beclin1, Atg5, etc., in both cell lines (Figure 1B). Upregulation of macro-autophagy-associated genes was observed in PBS-treated cells when compared with each respective control. Hence, the development of autophagic cell lines through nutrient starvation had been accomplished successfully. Next, in order to investigate the autophagic effect, we assessed how nutritional deficiency modulates cellular viability under cisplatin treatment. To accomplish our hypothesis that nutritional deficiency somehow differentially regulates anti-cancer drug cytotoxicity, first, we determined the optimum cytotoxic dose (IC_50_) of cisplatin in both cell lines. MTT assay data (Figure 1C) show that the minimum inhibitory concentration to reduce cellular viability to 50% from its initial concentration was 50 µM for HEK-293 and 30 µM for ACHN cells. For the comparative analysis of viable and non-viable cells in autophagic conditions alone and in combination with the chemotherapeutic drug (cisplatin), these particular doses were further implicated in the trypan blue exclusion assay (Figure 1D). Observation suggests that in both cell lines, a short duration of nutrient deprivation (3 h) did not result in a significant drop in cellular reproducibility. However, compared to normal cells, cancer cells treated with cisplatin under starving circumstances displayed a higher level of therapeutic sensitivity and exhibited rapid declination in cellular viability.

### 3.2. Identification of Major Biological Pathways Regulated by All Differentially Expressed Genes (DEGs)

**Figure 2:**
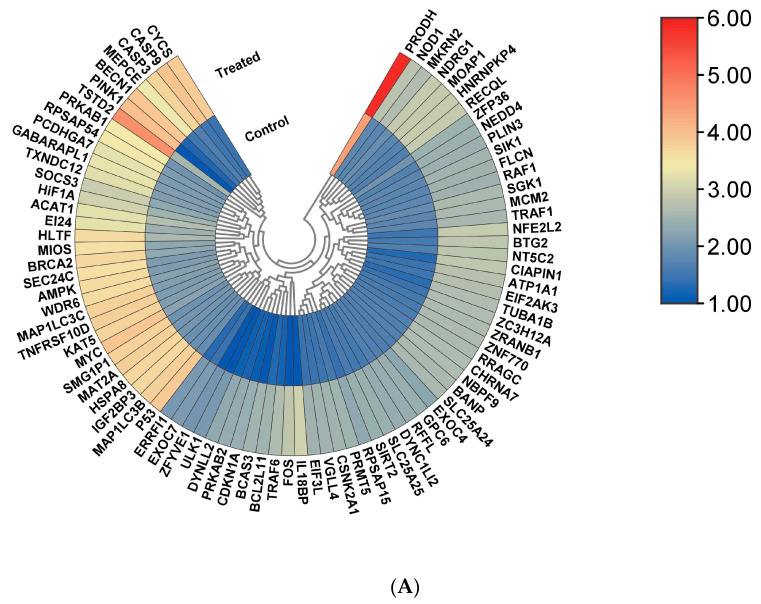

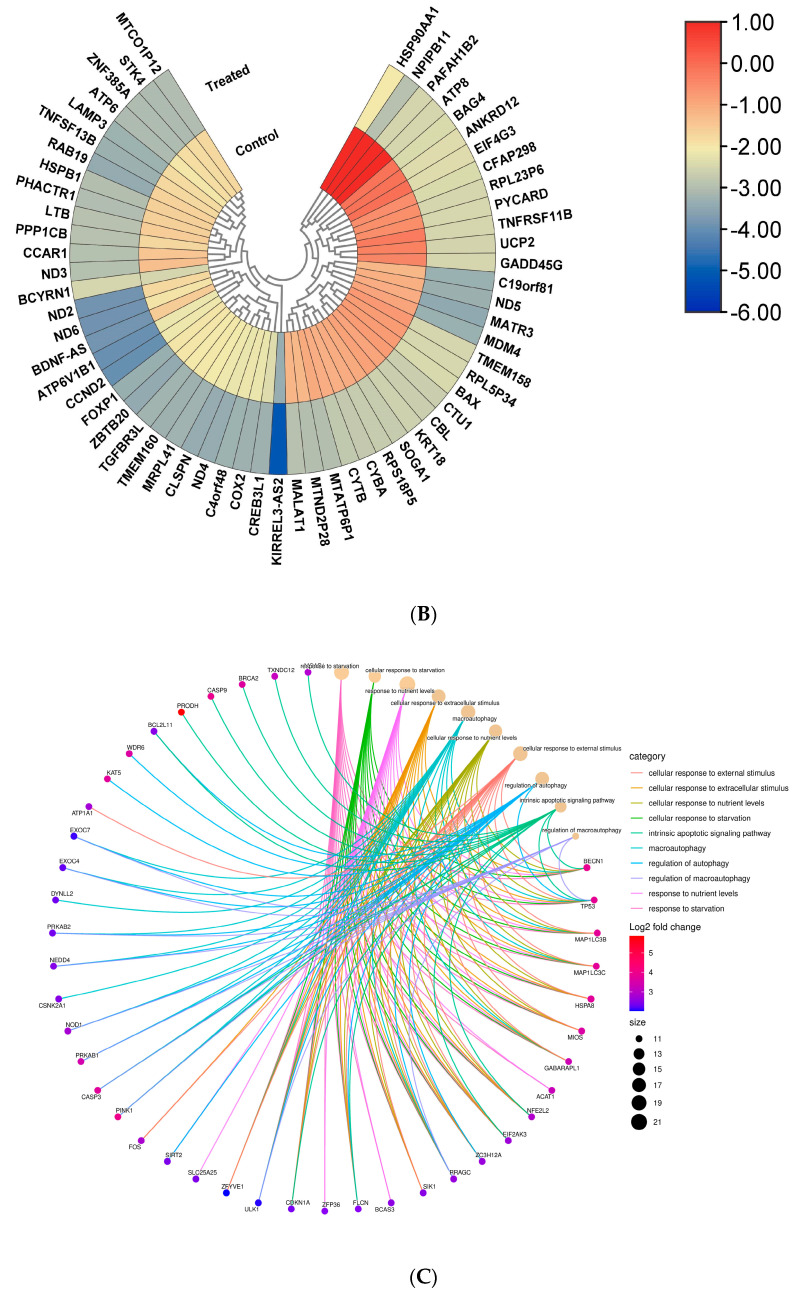

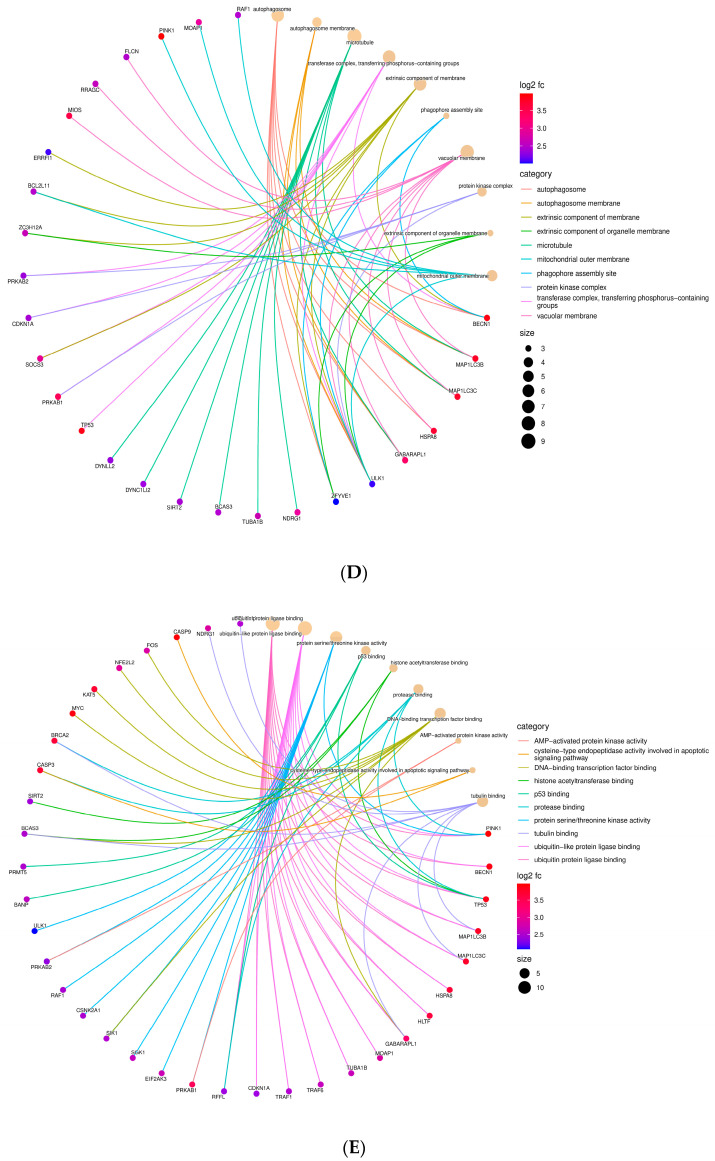

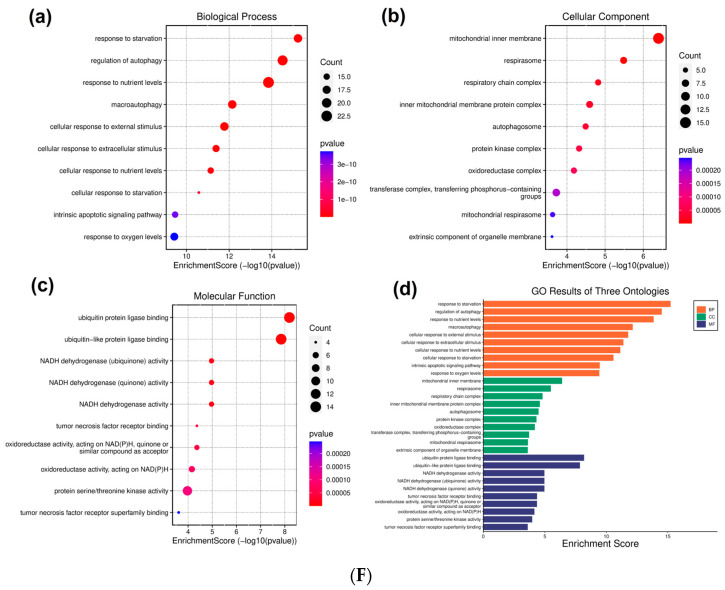
(**A**) Hierarchical clustering of differentially expressed up−regulated and (**B**) down−regulated genes in autophagic-treated (cisplatin-treated autophagic ACHN) and autophagic non-treated ACHN (control) cell lines. Heat−map was generated by TBtools (https://github.com/CJ-Chen/TBtools/releases) with the FPKM (fragments per kilobase of transcript per million mapped reads) value of both samples. (**C**) Cnet plot of genes regulating major biological processes (BP); (**D**) Cnet plot of major cellular components involved (CC); (**E**) Cnet plot of major molecular function (MF)-regulating genes. The plots were generated by SRPLOT (http://www.bioinformatics.com.cn/srplot) based on the results of the enriched KEGG pathway. Size = number of differentially expressed genes in the enriched KEGG pathway; fold change = the fold change difference between cisplatin-treated autophagic ACHNs and non-treated autophagic ACHNs. (**F**) Bubble plot showing GO results of all three ontologies ((**a**–**c**): bubble plot showing significant pathways for up-regulated DEGs in terms of BP, CC, MF respectively. Larger bubbles indicate higher number of genes. The colour of each bubble reflects significance; (**d**): combined GO results of three different ontologies) with defined enrichment score.

Autophagic ACHN cell lines and cisplatin-treated autophagic ACHN cell lines were subjected to transcriptomic profiling to identify major genes responsible for increased cytotoxicity under starvation conditions. Raw data retrieved from sequencing were immediately submitted to the NCBI sequence read archive (SRA) with bio-project accession numbers PRJNA926369 and PRJNA926370 for the autophagic cell line and cisplatin-treated autophagic cell line, respectively. The former generated nearly 3.28 GB of data with 1,08,78,317 paired-end reads (total number of bases = 3,285,251,734) and a GC% score of 48.48%. The latter one generated 3.74 GB of data with 1,23,87,553 paired-end reads (total number of bases = 3,741,041,006) and a 49.77% GC score (Table S3). The differential gene expression profiling through EdgeR shows that approximately 146 genes significantly controlled the drug-mediated toxicity under an autophagic environment. Among them, 86 genes with >2 log2 fold change were significantly upregulated, and 60 genes with >−2 were significantly downregulated (Tables S4 and S5). Heatmap generation based upon DEGs provides a complete profiling of significantly altered expression of each gene (Figure 2A,B). Thereafter, to determine the major biological pathways influenced by all identified DEGs, we subjected them to one bioinformatic analysis tool, SRPLOT [24]. From the biological process (BP) analysis, as shown in the cnet plot, we observed that all significantly expressed genes were associated majorly with cellular response to external stimuli, particularly in response to starvation and autophagy and intrinsic apoptosis signaling pathway (Figure 2C). Cellular components (ccs) involved in the generation of autophagosomes, inner mitochondrial membrane formation, oxidoreductase complex, protein kinase complex, and respirasome (respiratory chain complex) formations were involved (Figure 2D). Molecular function (MF) indicates that genes regulating NADH dehydrogenase (quinone and ubiquinone), serine/threonine kinase activity, tumor necrosis factor receptor, and ubiquitin-like protein ligase binding were significantly altered (Figure 2E). A complete gene ontology (GO) result based on the enrichment score of these three categories (cellular component, molecular function, and biological process) is shown (BAR DIAGRAM), which corroborates with all previous observations (Figure 2F). Enrichment analysis of all DEGs through the David-based SRPLOT tool (http://www.bioinformatics.com.cn/srplot) generates the emamplot, Go plot, and enrichment score bar plots, which were also consistent with the previous findings (Figures S1–S10).

### 3.3. Protein–Protein Interaction (PPI) Network and Pathway Network Analysis

**Figure 3:**
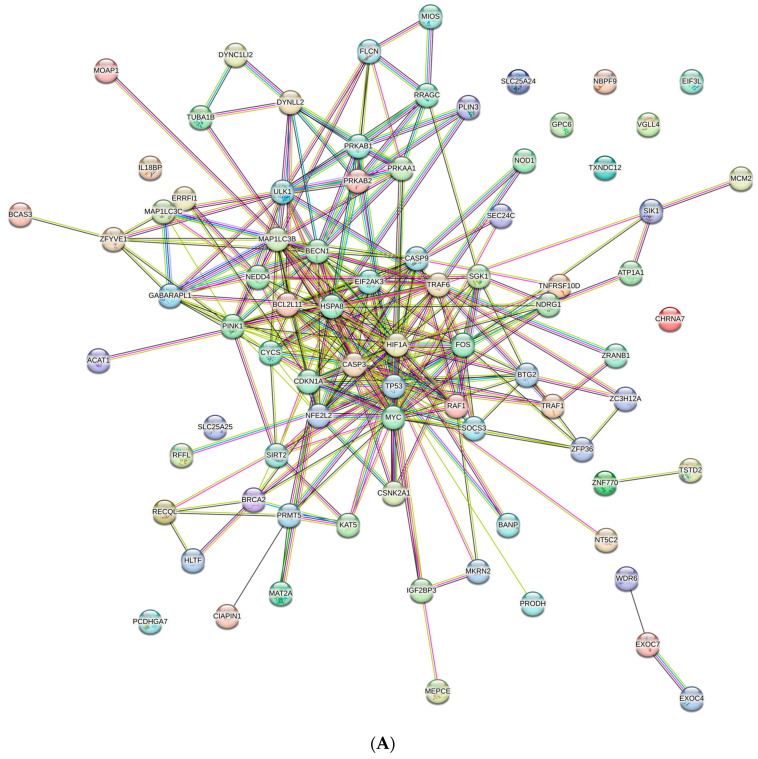

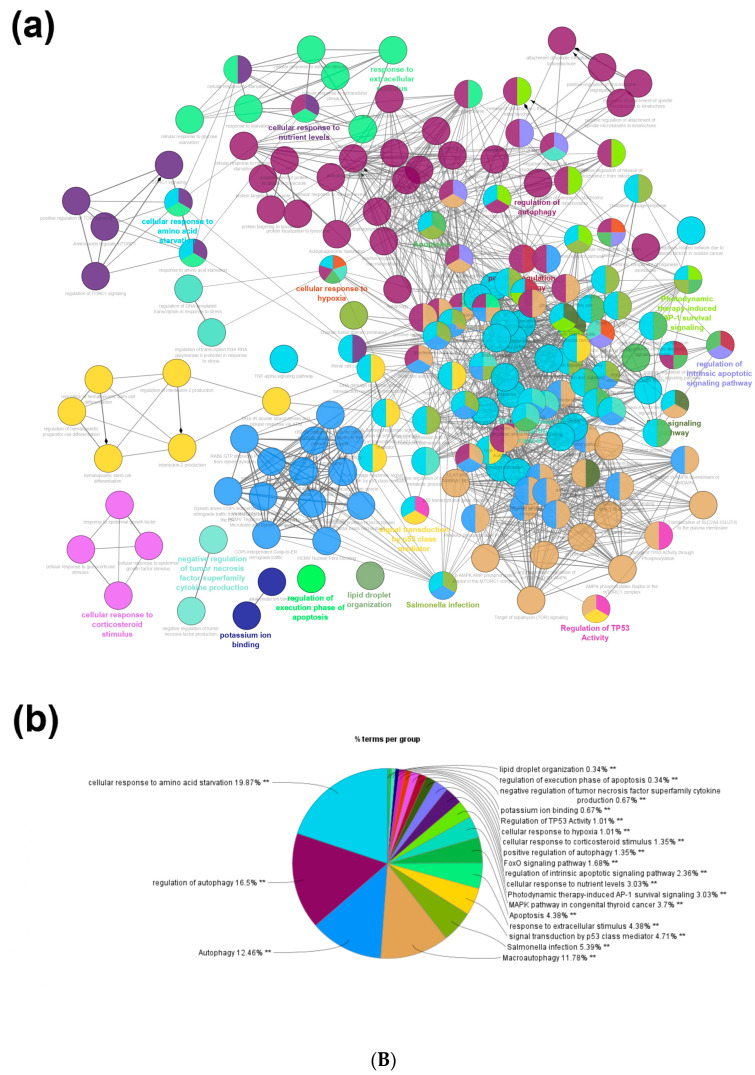

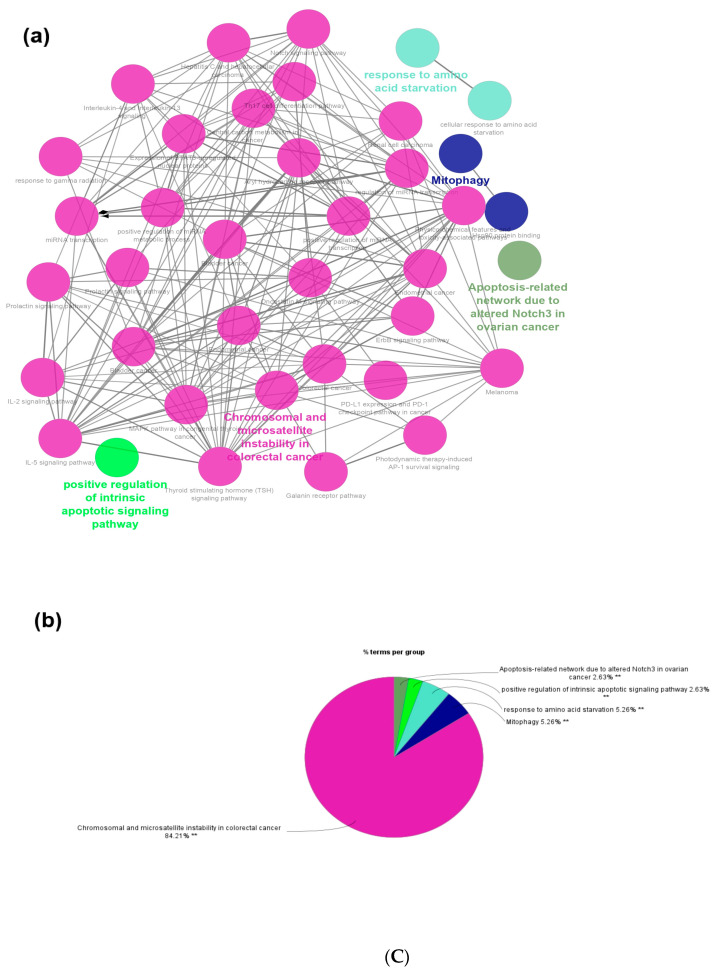

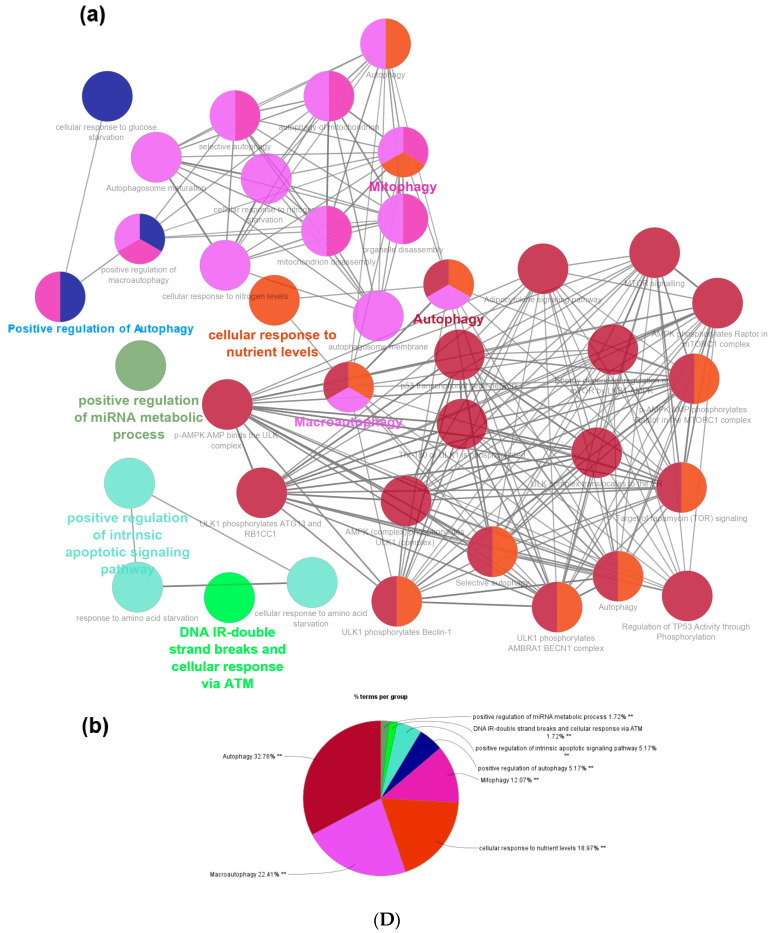
(**A**) Protein–protein interaction network constructed using STRING (Search Tool for the Retrieval of Interacting Genes/Proteins, version 11.5; https://string-db.org) with significantly upregulated DEGs in autophagic ACHNs in response to cisplatin. (**B**) Pathway network constructed using all the upregulated DEGs (**a**); Pie chart shows all the significantly up-regulated pathways (**b**). (**C**) Constructed pathway network from MCODE Cluster 1 generated using Cytoscape plugin ClueGO (**a**); pie chart shows the stress associated with overexpressed functional categories of the ClueGo pathway analysis (**b**). (**D**) Constructed pathway network from MCODE Cluster 2 generated using Cytoscape plugin ClueGO (**a**); pie chart shows the autophagy- and apoptosis-associated overexpressed functional categories of ClueGo (**b**) pathway analysis (** *p* < 0.001). The significantly enriched pathways are denoted by different colors.

Based on known inputs from several databases, such as text mining, high-throughput studies, conserved co-expressions, genomic context predictions, and other sources, the STRING database forecasts interactions between genes and proteins [25]. The upregulated gene network was anticipated to have 82 nodes and 296 edges with an average node degree of 7.22 and PPI enrichment value < 1.0 × 10^−16^ (Figure 3A), where differentially colored edges serve as indicators of the interactions. The downregulated gene network was anticipated to have 50 nodes and 52 edges with an average node degree of 2.08 and a PPI enrichment value of 1.1 × 10^−11^. All interactions with less than 0.4 indicate a fair level of confidence in the data used to forecast these interactions (Figure S11). Thereafter, ClueGO, a Cytoscape plugin, was used to examine all PPI networks [28], and most of the interactions among upregulated DEGs were found to be associated with cellular response to amino acid starvation (19.87%), regulation of autophagy (16.5%), mammalian autophagy (12.46%), macro-autophagy (11.78%), apoptosis (4.38%), signal transduction by p53 class mediator (4.71%), response to extracellular stimulus (4.38%), cellular response to nutrient level (3.03%), etc. (Figure 3B and Figure S12). Notably, the downregulation of genes associated with respiratory electron transport and ATP synthesis (80%), mitochondrial oxidative phosphorylation (12.31%), interferon production (4.62%), etc., were found (Figures S13 and S14).

An additional layer of functional annotation was achieved through MCODE over conventional guilt-by-association technique, which allow us to locate molecular complexes (clusters) in the broad PPI network [27]. The network modules identified by MCODE using a graph-theoretic approach are based on the density of connections between interacting nodes. The approach initially selects the “seed” node, following which its immediate neighbors are found. When neighboring nodes meet a set of criteria, such as minimum degree or density of connections, the cluster is then gradually expanded, and the operation is eventually completed when there are no more nodes that can be added to the cluster. As soon as a module was discovered, MCODE assessed it based on its size, density, and connectivity; higher-scoring modules were considered to be more significant. MCODE can prioritize finding larger or more significant modules and can also spot overlapping modules. A cluster’s sub-units typically contribute to the same biological objective, making MCODE an effective tool for predicting unknown proteins. Two clusters with substantial scores were created from our PPI input (Figures S15 and S16). Each cluster was then subjected to route analysis using the Cytoscape plugin ClueGO (v3.9.1), which incorporates the chromosomal location (CO), biological process (BP), cellular component (CC), molecular function (MF), immune system (IS) process, gene ontology (GO) terminology, and additional enriched molecular pathways including the CORUM, Reactome, KEGG, InterPro, and WikiPathways databases. Cluster 1 forms with 54 nodes and 272 edges with a cluster score of 10.56, and Cluster 2 forms with 64 nodes and 290 edges with a cluster score of 6.73. The pathway analysis of Cluster 1 revealed proteins that were predominantly engaged in positive regulation of apoptosis-regulating networks (5.26%), cellular response to starvation (5.26%), mitophagy (5.26%), pathways associated with chromosomal instability (84.21%), and others. (Figure 3C and Figure S17). While considering Cluster 2, proteins participating in autophagy (32.76%), macro-autophagy (22.41%), cellular response to nutrient level (18.97%), mitophagy (12.70%), positive regulation of intrinsic apoptosis signaling pathway (5.17%), and others were found (Figure 3D and Figure S18).

### 3.4. Major Regulatory Hub Genes Identification by Cytohubba Algorithm and Their Functional Analysis

The Cytohubba plugin’s 12 algorithms were used to score the proteins [29,34], and we picked the top-performing candidate in the majority of algorithms. Here, based on the quantity of edges, the degree algorithm ranks the nodes (genes). Nodes with a high degree are given more weight by the network (Table S6). The centrality of a node within a network is used by the betweenness method to rank them. A high betweenness node is typically used as a bridge between two networks. By measuring how tightly neighbors are related, the clustering coefficient method ranks nodes. A densely connected subnetwork is more likely to contain nodes with high clustering coefficients. Based on their proximity to neighboring nodes, nodes are ranked according to the closeness algorithm. High-proximity nodes are closer together and have shorter paths to other nodes, making them more central to the network. The maximum neighborhood component (MNC) determines a node’s significance to a certain network based on its position. The average shortest path length between all network nodes is used to calculate this statistic. The distance between a node and every other node in the network is referred to as a node’s eccentricity. It displays the distance between a node and other nodes in the network. This symbolizes a node’s “centrality” inside the network. Less out-of-the-ordinary nodes are thought to be more central and to have a bigger impact on the network. It is believed that MNC radiality nodes are more important to the network and have a greater impact on its dynamics, whereas nodes known as bottleneck nodes are those that, if eliminated from a network, would seriously impede communication or information flow. In order to construct network control mechanisms, it can be useful to identify bottleneck nodes. The maximal clique centrality (MCC) computes each node’s centrality by identifying the biggest cliques (fully linked subgraphs) in a network. High MCC nodes are important because they support preserving the stability and robustness of the network. The number of shortest pathways that pass through a node in a network determines its degree of stress centrality. The largest group of nodes in a network that are connected by direct or indirect edges are ranked by EPCs. According to the DMNC algorithm, the seed node, or node with the highest degree (the node with the most connections to other nodes), should be found first. A seed node and all of its neighbors are contained in a subgraph known as a maximum neighborhood component (MNC). The MNC is subsequently located using the MNC algorithm. The ratio of the number of edges to the total number of potential edges within the MNC is used to calculate the DMNC. It enables the density of the MNC to be calculated. To establish the radiality, the shortest path between each network node and each other node must first be identified. The measure is calculated by summing up the inverse distances between each node and each other node in the network.

**Figure 4:**
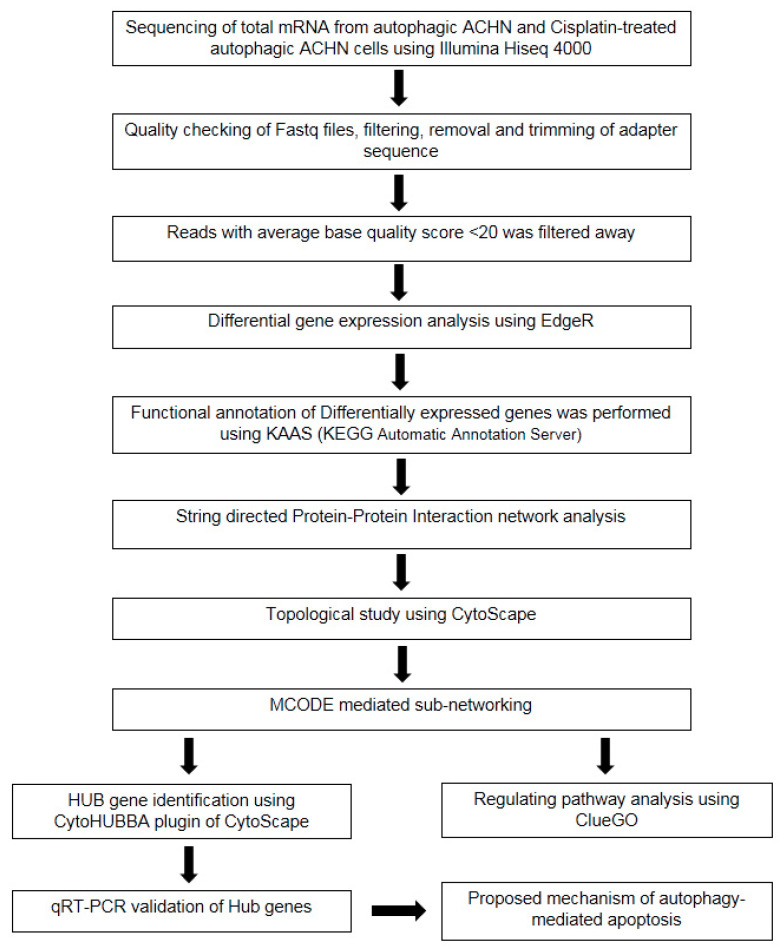
Sequential workflow of bioinformatics analysis pipeline. Flowchart describing the steps of data processing and subsequent analysis of differentially expressed genes.

Together, all these algorithms identify the key metabolic pathways controlled by top-ranked hub genes, which were the AMPK signaling pathway, FOXO signaling pathway, p53 signaling pathway, autophagic pathway (including mitophagy), and apoptosis pathway (Figure S19). The top-ranked prime hub genes, *MYC* (proto-oncogene codes for myc protein; controls cell cycle, apoptosis, cellular metabolism, and mitochondrial biogenesis), *BECN1* (mammalian orthologue of *ATG6*; interplays between autophagy- and apoptosis-associated pathways), *MAP1LC3B* (encodes LC3B protein and is required for autophagy progression), *TP53* (tumor suppressor p53-encoding gene, which is associated with apoptosis), and *HIF1A* (hypoxia-inducing factor, which is involved in several carcinoma, playing significant role in regulating autophagy and cellular growth), were predicted by ten algorithms, whereas several apoptosis inducer genes, like *CASP3* (encodes caspase3 and leads to plasma membrane blabbing, DNA damage, and phosphatidylserine exposure outside the plasma membrane during apoptosis), *CASP9* (caspase9, a major regulator of intrinsic mitochondrial apoptosis), *CDKN1A* (encodes p21 and is a cyclin-dependent protein kinase, which suppresses cellular growth through JAK/STAT signaling pathway), and *CYCS* (encodes cytochrome c and is a regulator of apoptotic pathway through caspase activation), were predicted by eleven, eight, and seven major algorithms, respectively. Hub genes like *ULK1* (major activator of mammalian autophagy by mTOR inactivation under starvation), *EIF2AK3* (eukaryotic translation initiation factor 2-alpha kinase 3; controls apoptosis and autophagy), and *BCL2L11* (known as bim; involved in FOXO signaling to direct cellular apoptosis) were also predicted by these algorithms (Table S6). A complete workflow of bioinformatics study is depicted in Figure 4.

### 3.5. Validation of Differentially Expressed Genes by Quantitative Real-Time PCR and Apoptosis Study for Determining Cell Death Induced by Cisplatin under an Autophagic Environment

**Figure 5:**
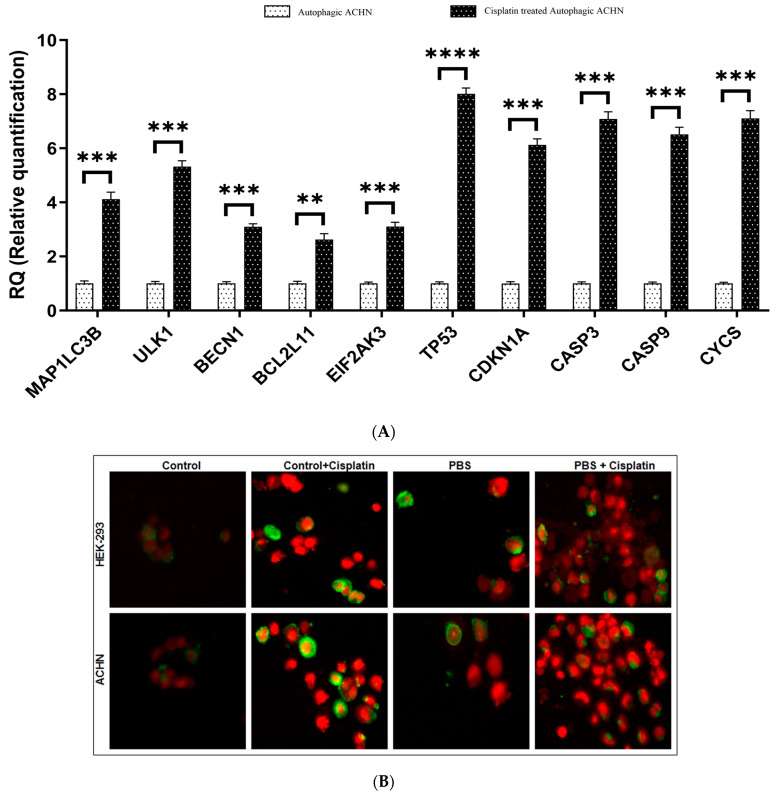
(**A**) Validation of RNAseq data by measuring the relative expression level of 10 differentially expressed genes in the ACHN cell line in control (autophagic ACHN cells without cisplatin) and treated (cisplatin-treated autophagic ACHN cells) cell lines by qRT-PCR. Values are represented as ±SD of at least three independent experiments. *p* < 0.05 was considered significant (**: *p* ≤ 0.01, ***: *p* ≤ 0.001, ****: *p* ≤ 0.0001); the standard deviations of the data have been shown in the form of error bars. (**B**) Cell death was recorded in HEK-293 and ACHN cells through annexin V-FITC/PI assay—HEK-293 and ACHN cells were treated with cisplatin in nutrient-sufficient (control + cisplatin) and nutrient-deficient conditions (PBS + cisplatin). Slides were stained according to the manufacturer’s instructions, and visualization was achieved under the 40× objective of a fluorescence microscope (Magnus MLXi, India). Each image is a representative of at least three independent biological experiments.

To validate in silico findings, the expression of identified hub genes was quantitatively estimated by real-time PCR analysis using the SYBR green method. The chosen upregulated hub genes include *BECN1*, *ULKI*, and *MAP1LC3B*, which regulates autophagy, and *EIF2AK3*, *BCL2*, *CDKN1A*, *CASP9*, *CASP3*, *CYCS*, which are mainly involved in different cell death regulatory pathway. The primers used during PCR amplification are listed in Table S6. The resultant study summarizes that the in vitro data obtained were in good agreement with transcriptomic data, indicating the reliability and accuracy of the transcriptomic-based findings (Figure 5A). All the amplified genes were also checked on agarose gel (Figure S20).

As the major regulatory hub genes were linked to apoptosis/necrosis-associated cell death and to correlate this with the synergistic mechanism of starvation and cisplatin in a dose-dependent manner, we continuously monitored the cells in each mentioned condition. In Figure 5B, some early apoptotic HEK-293 cells were viewed under the combinatorial treatment, while necrosis/late apoptotic ACHN cells were clearly visible after 3 h of treatment. The abundance of late apoptotic cells confirmed our previous hypothesis that starvation has differentially sensitized cells in response to cisplatin toxicity, and the identified hub genes were in association with this metabolic tuning. Results obtained from indirect ELISA assay also corroborated the above findings (Figure S21).

## 4. Discussion

Over the last decade, several preclinical studies have extensively demonstrated the advantage of daily caloric restriction [35]. Reports suggest that fasting affects metabolic reprogramming on a systemic level via nutrient sensor pathway activation [8]. Surprisingly, under a nutrient-deficient environment, the dual stress responsiveness of cancer and normal cells led to the discovery of an interesting phenomenon known as differential stress resistance/differential stress sensitization [36,37,38]. The healthy cell undergoes metabolic rewiring that triggers autophagy, stimulating the internal damage repair mechanism, which provides protection against chemo-toxicity [5]. On the contrary, tumor cells are more vulnerable to nutrient deficiency. As tumor cells rely on anaerobic glycosylation for ATP production (Warburg effect), the increased metabolic activity requires an abundance of energy to meet their requirement in terms of a high proliferation rate [3]. However, fasting directs cancer cells toward a metabolic transition to oxidative phosphorylation, known as the anti-Warburg effect [2,39]. Our choice of applying cisplatin after fasting-derived autophagy induction relies on previous findings that report improved chemotherapeutic efficacy under a restricted diet or fast [40,41,42]. Besides this, as nephrotoxicity emerges as the major backlog associated with cisplatin treatment [43], we tried to eliminate such side effects by restricting drug exposure time through dose-dependent assessment.

Our data clearly demonstrate that differential autophagic stress response (imposed by PBS) has made the cancer cells more susceptible to cisplatin toxicity, whereas normal cells could easily withstand the same toxicity. Cellular viability data obtained from MTT assay (Figure 1C) and trypan blue exclusion assay (Figure 1D) proved the hypothesis that cisplatin efficiently decreased the ACHN cell growth (30 µM) under an autophagic environment, with minimal inhibition of HEK-293 (50 µM) viability. AnnexinV/FITC staining also corroborates with the previous findings, indicating a direct involvement of apoptosis behind autophagy-mediated increased cytotoxicity of the chemotherapeutic agent (Figure 5B). An extensive survey of transcriptionally regulated genes under cisplatin non-treated (considered the control) and cisplatin-treated autophagic (considered the treated) conditions were then considered to obtain an insight into the major regulatory pathways influencing such phenomenon. ClueGO analysis reveals (Figure 3C,D) that the pathway generated in PPI (protein–protein interaction) network includes the significantly upregulated genes, which are mostly associated with autophagy (12.46%) and cellular response to starvation (19.6%). Similarly, we observed that genes involved in respiratory electron transport (80%) and oxidative phosphorylation are mostly downregulated (Figure S13). Thus, our data provide a metabolic strategy for cisplatin-mediated oxidative phosphorylation inhibition under nutrient restriction. The above observational findings, if generalized, suggest that cisplatin acts upon cancer cells’ anti-Warburg response under a nutrient-deficient environment [2,39]. A comparative cell death analysis was simultaneously performed on normal HEK-293 cells (Figure 5B). Data curated from wet-lab experiments ensure that short-term exposure to a chemotherapeutic drug itself could not inhibit normal cells’ reproducibility because of their differential stress response under starvation [2,13]. Studies reported on metformin under nutrient restriction [41,44] also suggest that dietary restriction imposes additional toxicity over oxidative phosphorylation inhibition by metformin and thus implicates a “dual-whammy” effect on cancer cells. However, targeting glycolysis by non-selective pharmacological targets might elicit an adverse effect on normal cells [45,46]. Therefore, modulating a physiological autophagy inducer (starvation) with restricted drug exposure time (in this case, cisplatin), a novel therapeutic strategy, has been summarized here to specifically target renal carcinoma.

To the best of our knowledge, no reports have been published yet that identify master regulator(s) controlling cisplatin toxicity under starvation-mediated autophagy in renal carcinoma. Therefore, we analyzed the DEGs under the Cytohubba plugin ver. 1.5.1 using 12 different algorithms and identified the top-ranked genes governing selective pathways that ClueGO has previously examined [34,42]. The significant differential expression/interaction among genes controlling cellular growth and proliferation leads to autophagy-mediated apoptosis in ACHN cells. There are several key metabolic pathways were previously reported to control cancer cell proliferation by chemotherapeutic drugs under autophagic conditions [42]. In this study, we found that multiple genes involved in cell proliferation/inhibition act canonically to achieve remarkable efficacy. The differentially expressed gene *MYC*, which controls cellular growth, proliferation, and apoptosis, is a potential therapeutic target for controlling cancer growth [47]. Overexpression of this proto-oncogene provides resistance against chemotherapeutic agents, which can be reversed by the *MYC* gene-silencing method [47,48]. The interconnection of this particular gene with autophagy-associated *BECN1*, *ULK1*, and *MAP1LC3B* and apoptosis-associated genes, namely *TP53*, *EIF2AK3*, *BCL2L11*, *CDKN1A*, *CASP3*, *CASP9*, etc., suggests a strong autophagy-dependent apoptosis progression in the ACHN cell line. The literature suggests that AMPK activation via PERK (*EIF2AK3*) causes *bcl2*-dependent mitochondrial apoptosis [49]. In an AMPK-dependent manner, PERK accelerated mitochondrial apoptosis and significantly contributed to the defective mitochondrial oxidative metabolism by suppressing the TCA cycle (tricarboxylic acid), oxidative phosphorylation, and pyrimidine production [49], which was consistent with our findings from ClueGO analysis, which suggests 80% downregulation of oxidative phosphorylation genes (Figure S13). The upregulation of caspase-associated genes (*CASP3*, *CASP9*) and cytochrome-encoding genes (*CYCS*) are also supporting the findings. Similarly, a decrease in ATP (during nutrient deficiency) caused by Ulk1-triggered autophagy (especially mitophagy) causes nonmitophagic mitochondria to overwork the electron transport cycle to meet energy demands and unintentionally contributing to ROS overproduction, ultimately leading toward apoptosis [50]. When Ulk1 enters mitochondria, it blocks manganese dismutase action and increases superoxide formation [50]. Under such elevated autophagic conditions, activated *EIF2AK3* leads to cellular apoptosis through caspase activation as soon as cisplatin is introduced. Subsequently, a cell cycle-dependent kinase inhibitor target gene (*CDKNIA*; encodes p21) present downstream of *TP53* has been known to be capable of promoting apoptosis in a variety of tumor types by either activating the TNF receptor or generating the proapoptotic protein bcl2 [51,52]. It regulates cell apoptosis by mediating changes in mitochondrial membrane permeability, too. Notably, the activation of the hypoxia-inducible factor (*HIF1*) and *MYC* gene suggests cancer cells’ defense mechanism (Table S4) modulates anti-apoptotic signals to survive under acute stress conditions; however, the inhibition of these through several mechanistic approaches suggests a significant positive response for controlling cancer cell proliferation [53,54]. Since combinatorial treatment offers a promising transition from autophagic sensitized cells toward apoptotic cell death, this can be an effective therapeutic strategy to control renal carcinoma. However, one disadvantage of this approach could be that some cancer patients are metabolically compromised to withstand the fasting period [2]. In those particular cases, careful monitoring of treatment response should be performed to maintain clinical efficacy and safety [3,55,56]. Together, the insights described here will open a new therapeutic window to improve chemotherapy against renal carcinoma and encourage clinical trials that use combination therapy of fasting with cisplatin where no molecular therapy is significantly effective. Furthermore, we identified potential biomarkers regulating autophagy-mediated drug toxicity, which can be further evolved as molecular targets for inducing cytotoxicity in cancer cells without affecting normal cell proliferation.

## 5. Conclusions

The current study sheds light on the transcription-level response of cisplatin-treated starved renal carcinoma cells (ACHNs) and unfolds major regulatory pathways influencing additional sensitivity toward chemotherapy. This study will pave the way for clinical studies to begin for better drug efficacy during chemotherapy while on a restricted diet or fasting.

## Data Availability

The raw read sequence from each data set was submitted to the National Centre for Biotechnology Information (NCBI) and can be accessed in the form of a sequenced read archive (SRA) under the bio-project accession numbers PRJNA926369 and PRJNA926370.

## Supplementary Materials

The following supporting information can be downloaded at https://www.mdpi.com/article/10.3390/cells13060471/s1. Figure S1: KEGG enrichment analyses of the total DEGs (a), upregulated DEGs (b), and downregulated DEGs (c). The data in the figures present the number of enriched DEGs. Figure S2: Emap plot of enriched “Biological Process” gene ontology terms (*p* < 0.05, FDR < 0.05). The plot is generated by SRPLOT (http://www.bioinformatics.com.cn/srplot) tool based on the output of enriched biological processes. *p* value—Benjamini–Hochberg adjusted the *p*-value for the enriched ontology term. Size—number of DEGs belonging to enriched gene ontology term. Figure S3: Emap plot of enriched “Cellular component” ontology terms (*p* < 0.05, FDR < 0.05). The plot is generated by SRPLOT (http://www.bioinformatics.com.cn/srplot) tool based on the output of enriched biological processes. *p* value—Benjamini–Hochberg adjusted the *p*-value for the enriched ontology term. Size—number of DEGs belonging to enriched gene ontology term. Figure S4: Emap plot of enriched “Molecular function” ontology terms (*p* < 0.05, FDR < 0.05). The plot is generated by SRPLOT (http://www.bioinformatics.com.cn/srplot) tool based on the output of enriched biological processes. *p* value—Benjamini–Hochberg adjusted the *p*-value for the enriched ontology term. Size—number of DEGs belonging to enriched gene ontology term. Figure S5: Go plot of enriched “Biological Process” (*p* < 0.05, FDR < 0.05). The plot is generated by SRPLOT (http://www.bioinformatics.com.cn/srplot) tool based on the output of enriched biological processes. *p* value—Benjamini–Hochberg adjusted the *p*-value for the enriched ontology term. Size—number of DEGs belonging to enriched biological process. Color intensity indicates enriched biological processes. Figure S6: Go plot of enriched “Cellular components” (*p* < 0.05, FDR < 0.05). The plot is generated by SRPLOT (http://www.bioinformatics.com.cn/srplot) tool based on the output of KEGG analysis. *p* value—Benjamini–Hochberg adjusted the *p*-value for the enriched ontology term. Size—number of DEGs belonging to enriched cellular components. Color intensity indicates enriched cellular components. Figure S7: Go plot of enriched “Molecular function” (*p* < 0.05, FDR < 0.05). The plot is generated by SRPLOT (http://www.bioinformatics.com.cn/srplot) tool based on the output of KEGG analysis. *p* value—Benjamini–Hochberg adjusted the *p*-value for the enriched ontology term. Size—number of DEGs belonging to enriched molecular functions. Color intensity indicates enriched molecular activities of DEGs. Figure S8: Enrichment score plot of major “Biological Process” involved. The plot is generated by SRPLOT (http://www.bioinformatics.com.cn/srplot) tool based on the output of KEGG analysis. Figure S9: Enrichment score plot of major “Cellular components” involved. The plot is generated by SRPLOT (http://www.bioinformatics.com.cn/srplot) tool based on the output of KEGG analysis. Figure S10: Enrichment score plot of major “Molecular function” involved. The plot is generated by SRPLOT (http://www.bioinformatics.com.cn/srplot) tool based on the output of KEGG analysis. Figure S11: Protein–protein interaction network constructed using STRING (Search Tool for the Retrieval of Interacting Genes, version 11.5; https://string-db.org) with significantly downregulated DEGs in ACHN cell line response to starvation-induced autophagy and cisplatin. Figure S12: Enriched pathways in ACHN cell line response to starvation-induced autophagy and cisplatin, constructed from all the upregulated PPI networks using Cytoscape plugin ClueGO (v3.9.1). The significantly enriched pathways are denoted by different colors. Figure S13: Pathway interactions in ACHN cell line response to starvation-induced autophagy and cisplatin. The pathway network was constructed from all the downregulated PPI networks using Cytoscape plugin ClueGO (v3.9.1). (a) The significantly enriched pathways were denoted by different colors. (b) The pie chart shows the overrepresented functional categories of the ClueGO pathway analysis (** *p* < 0.001, * *p* < 0.01, without star *p* < 0.05). Figure S14: Enriched pathways in ACHN cell line response to starvation-induced autophagy and cisplatin, constructed from all the downregulated PPI networks using Cytoscape plugin ClueGO (v3.9.1). The significantly enriched pathways are denoted by different colors. Figure S15: MCODE Cluster 1 generated with all the upregulated DEGs using Cytoscape plugin MCODE. Cluster 1 forms with 54 nodes and 272 edges with a cluster score of 10.56. Figure S16: MCODE Cluster 2 generated with all the upregulated DEGs using Cytoscape plugin MCODE. Cluster 2 forms with 64 nodes and 290 edges with a cluster score of 6.73. Figure S17: Enriched pathways in ACHN cell line response to starvation-induced autophagy and cisplatin, constructed from cluster—1 using Cytoscape plugin ClueGO (v3.9.1). Figure S18: Enriched pathways in ACHN cell line response to starvation-induced autophagy and cisplatin, constructed from cluster 2 using Cytoscape plugin ClueGO (v3.9.1). Figure S19: Hub gene identification by KEGG pathway viewer by analyzing upregulated DEGs. a. AMPK signaling pathway; b. FOXO signaling pathway; c. p53 signaling pathway; d. autophagic pathway (including mitophagy); e. apoptosis pathway. Figure S20: Validation of transcriptomic data by qRT-PCR amplification of different identified HUB genes. M-100 bp DNA ladder (Takara), gene_C—control (cells without treatment); gene_T—PBS (nutrient deprived for 3 h). Figure S21: Antibody-based indirect ELISA was used to assess apoptosis in autophagic ACHN cells and cisplatin-treated autophagic ACHN cells. The cytosolic protein was extracted from each treatment condition, and quantification of apoptosis-related biomarkers was achieved by recording absorbance at 450 nm using SPECTROStar Nano plate reader (BMG Labteck, Germany). Table S1: Quantification of RNA from autophagic ACHN (control-AT1) and cisplatin-treated autophagic ACHN (treated AT2) cell lines. Quantification was carried out at 260/280 nm by NanoDrop plate reader (BMG Labteck, Germany). Table S2: Details of primers used in qRT-PCR for validation of RNAseq results. Table S3: Sequencing statistics obtained from Illumina Hiseq 4000 paired-end sequencing, where AT-1 denotes the control (autophagic ACHN), and AT-2 denotes the treated (cisplatin-treated autophagic ACHN) cell lines. Table S4: All the significantly upregulated DEGs in response to combinatorial treatment of starvation-induced autophagy and cisplatin in ACHN cell line were generated by EdgeR package. Up to 86 upregulated DEGs were identified with log2Fc > 2 with adjusted *p*-value < 0.05. Table S5: All the significantly downregulated DEGs in response to combinatorial treatment of starvation-induced autophagy and cisplatin in ACHN cell line. The top 60 DEGs were identified by EdgeR with log2Fc > 2 with adjusted *p*-value < 0.05. Table S6: Top-ranked hub genes with individual scores determined by 12 different algorithms run by Cytoscape plugin Cytohubba.
