# Supplementary material for: Cisplatin and Starvation Differently Sensitize Autophagy in Renal Carcinoma: A Potential Therapeutic Pathway to Target Variegated Drugs Resistant Cancerous Cells
Source: Cells. 2024 Mar 7;13(6):471. doi: 10.3390/cells13060471 (PMC10968928; doi:10.3390/cells13060471)
Supplement: Supplementary file 1 [file cells-13-00471-s001.zip › cells-2845043-supplementary.pdf]

Cisplatin and Starvation Differently Sensitize Autophagy in Renal Carcinoma: A Potential Therapeutic Pathway to Target Variegated Drugs Resistant Cancerous Cells

Ankita Dutta [1], Subarna Thakur [2], Debasish Kumar Dey [3,*] and Anoop Kumar [1,*]

[1] Advanced Nanoscale Molecular Oncology Laboratory (ANMOL), Department of Biotechnology, University of North Bengal, Siliguri 734013, West Bengal, India; ankitadutta71994@gmail.com
[2] Department of Bioinformatics, University of North Bengal, Siliguri 734013, West Bengal, India;
subarna.thakur@nbu.ac.in
[3] Stephenson Cancer Center, University of Oklahoma Health Sciences Center, Oklahoma City, OK 73104, USA
* Correspondence: debasish-dey@ouhsc.edu (D.K.D.); anoop@nbu.ac.in (A.K.); Tel.: +91-353-2776354 (A.K.); Fax: +91-353-2699001 (A.K.)

Citation: Dutta, A.; Thakur, S.; Dey, D.K.; Kumar, A. Cisplatin and Starvation Differently Sensitize Autophagy in Renal Carcinoma: A Potential Therapeutic Pathway to Target Variegated Drugs Resistant Cancerous Cells. *Cells* **2024**, *13*, x. https://doi.org/10.3390/xxxxx

Academic Editors: Fulvio Reggiori and Shigeomi Shimizu

Received: 12 January 2024
Revised: 26 February 2024
Accepted: 3 March 2024
Published: 7 March 2024

Copyright: © 2024 by the authors. Submitted for possible open access publication under the terms and conditions of the Creative Commons Attribution (CC BY) license (https://creativecommons.org/licenses/by/4.0/).

Supplementary Figures

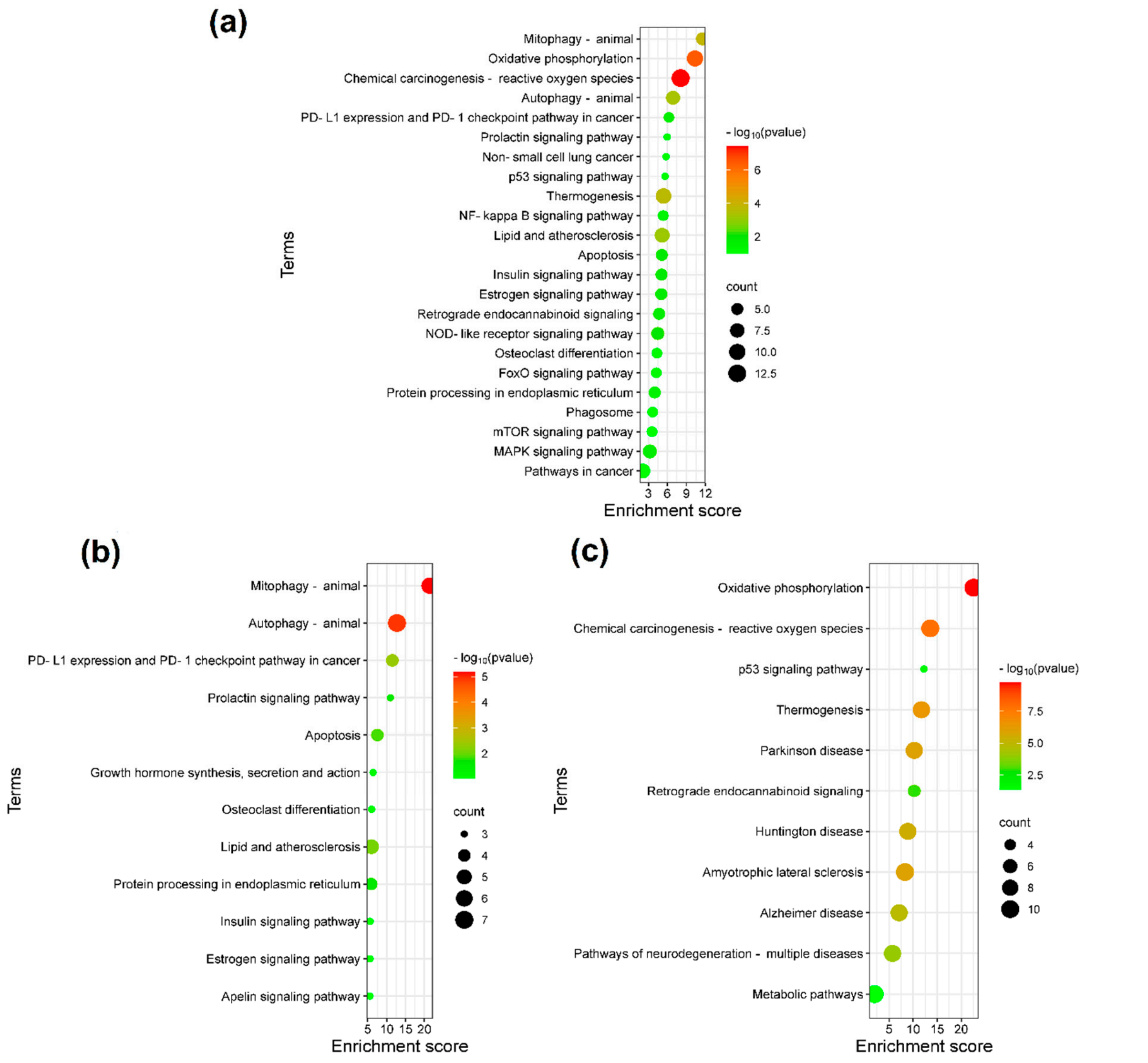

Figure S1. KEGG enrichment analyses of the total DEGs (**a**), upregulated DEGs (**b**), and downregulated DEGs (**c**). The data in the figures present the number of enriched DEGs

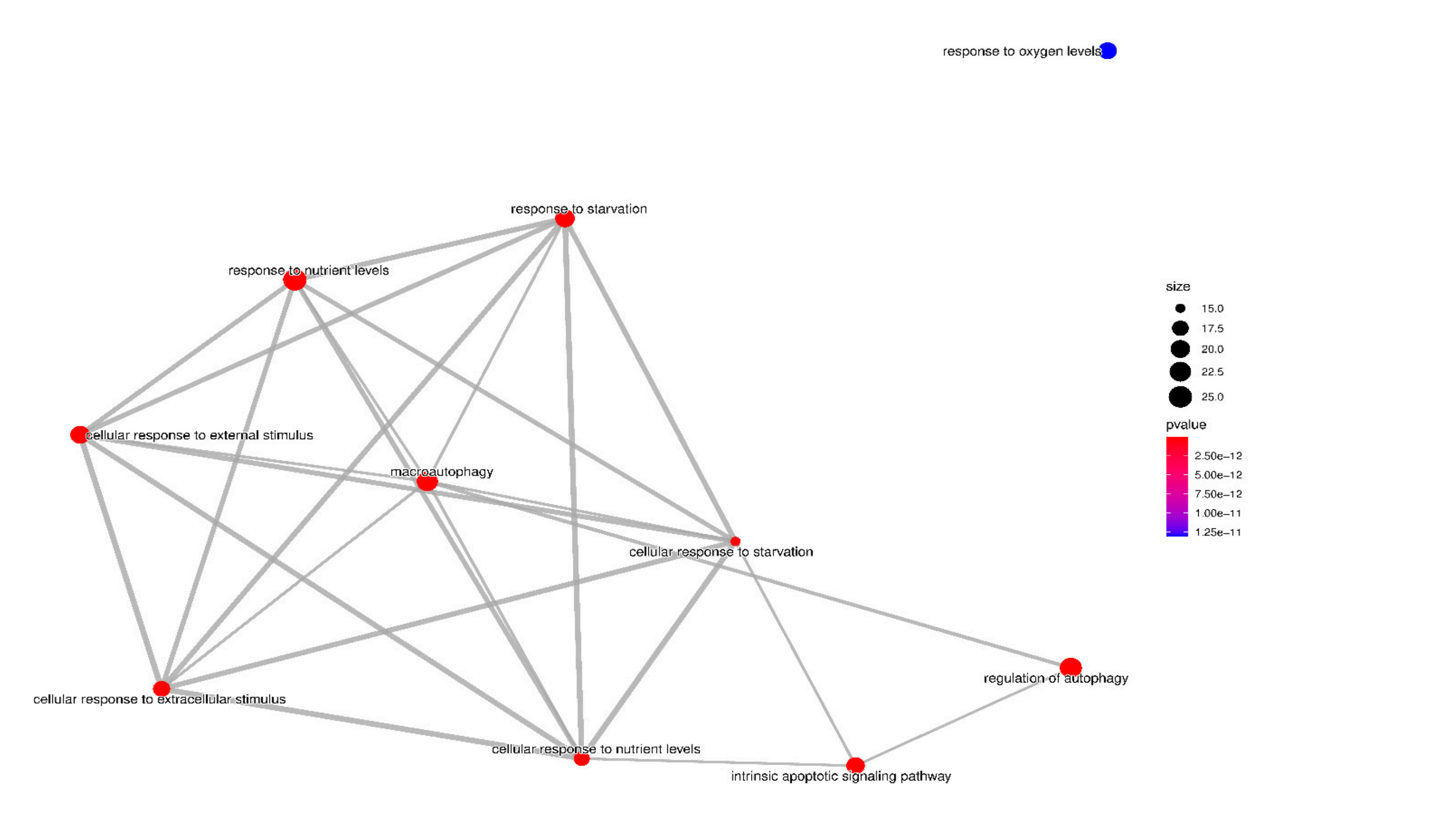

Figure S2. Emap plot of enriched “Biological Process” gene ontology terms (P < 0.05, FDR < 0.05). The plot is generated by SRPLOT (http://www.bioinformatics.com.cn/srplot) tool based on the output of enriched biological processes. P value - Benjamini–Hochberg adjusted P-value for the enriched ontology term. Size – number of DEGs belonging to enriched gene ontology term.

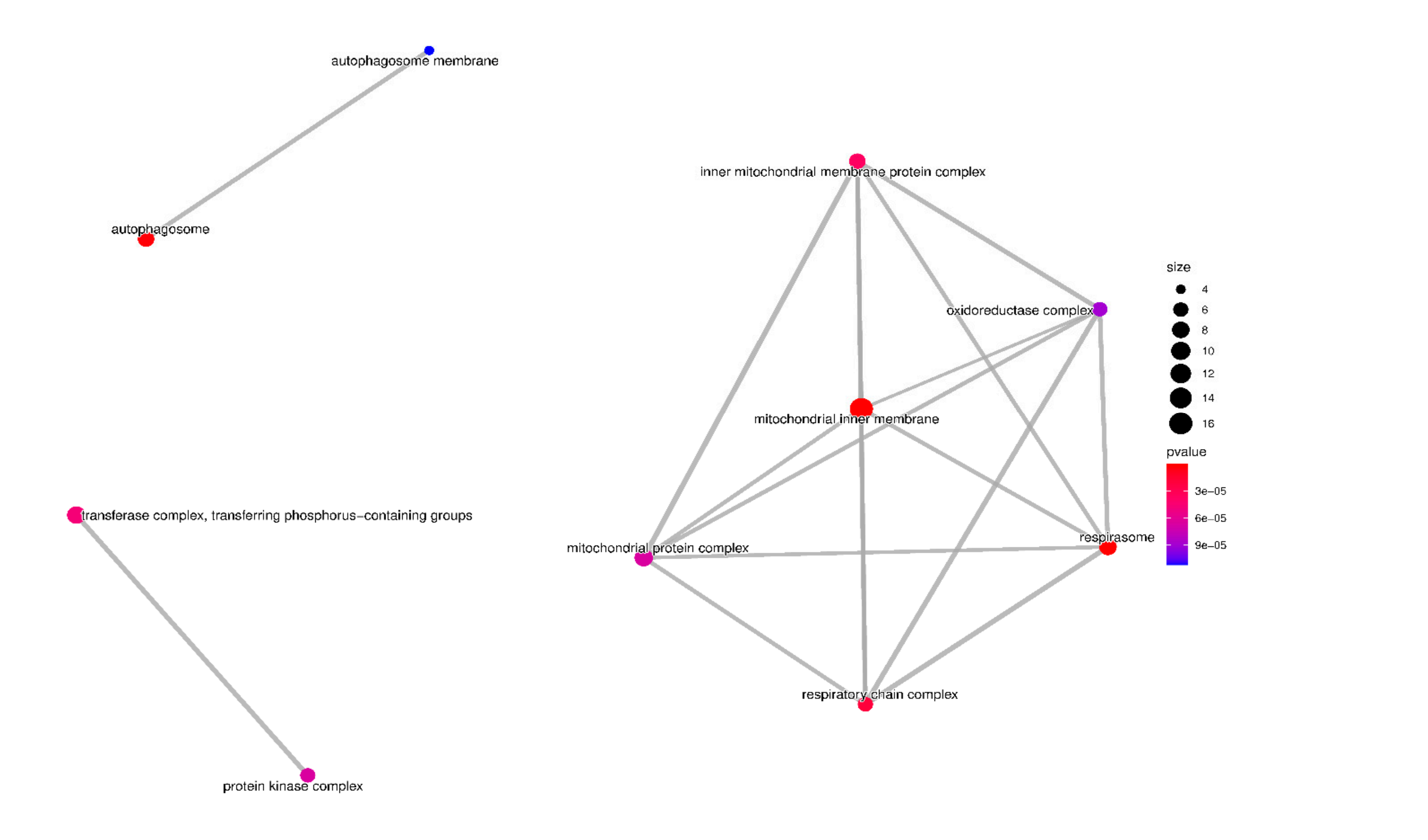

Figure S3. Emap plot of enriched "Cellular component" ontology terms (P< 0.05, FDR < 0.05). The plot is generated by SRPLOT(http://www.bioinformatics.com.cn/srplot) tool based on the output of enriched biological processes. P value - Benjamini–Hochberg adjusted P-value for the enriched ontology term. Size – number of DEGs belonging to enriched gene ontology term.

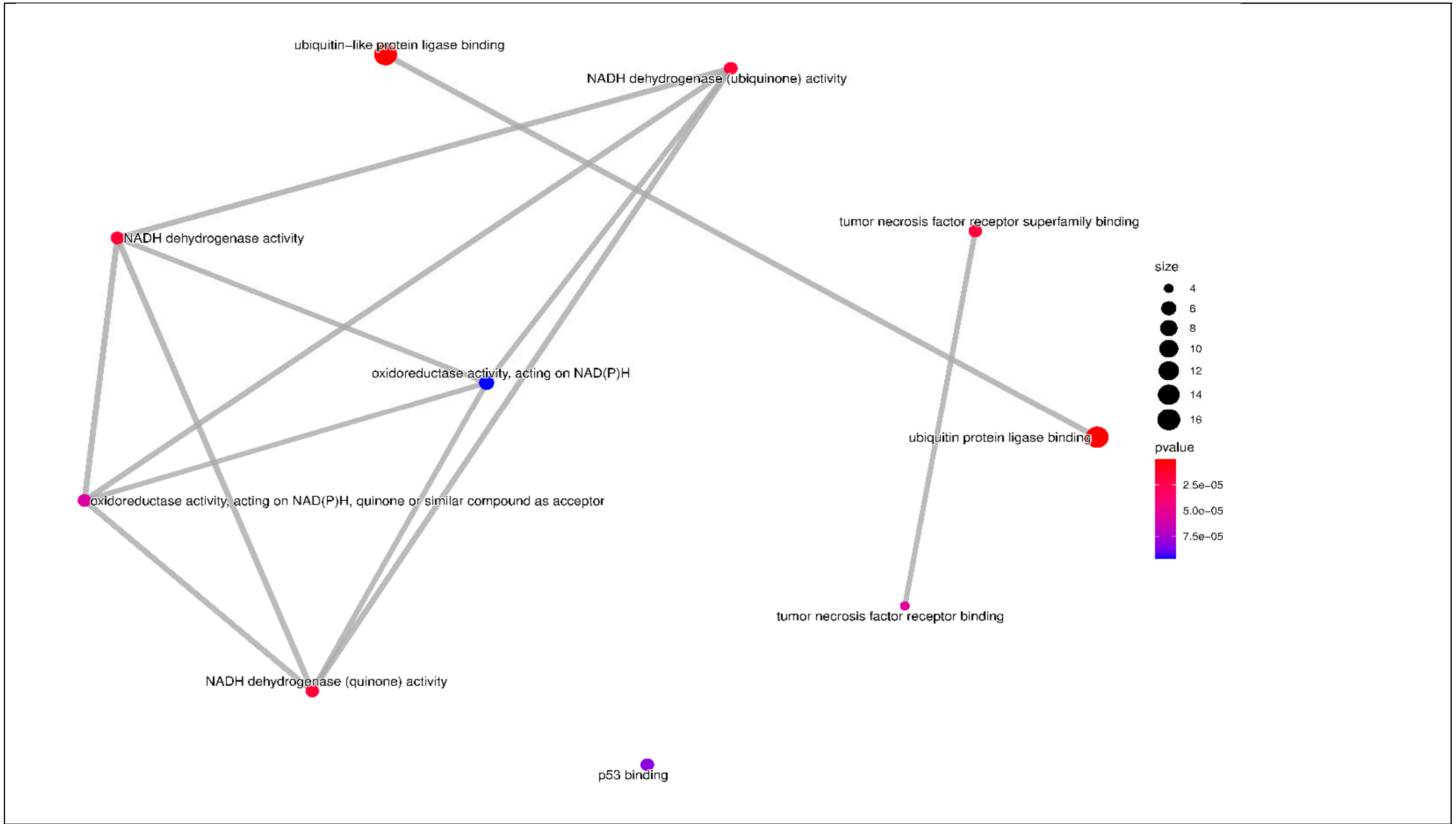

Figure S4. Emap plot of enriched "Molecular function" ontology terms (P< 0.05, FDR < 0.05). The plot is generated by SRPLOT(http://www.bioinformatics.com.cn/srplot) tool based on the output of enriched biological processes. P value - Benjamini–Hochberg adjusted P-value for the enriched ontology term. Size – number of DEGs belonging to enriched gene ontology term.

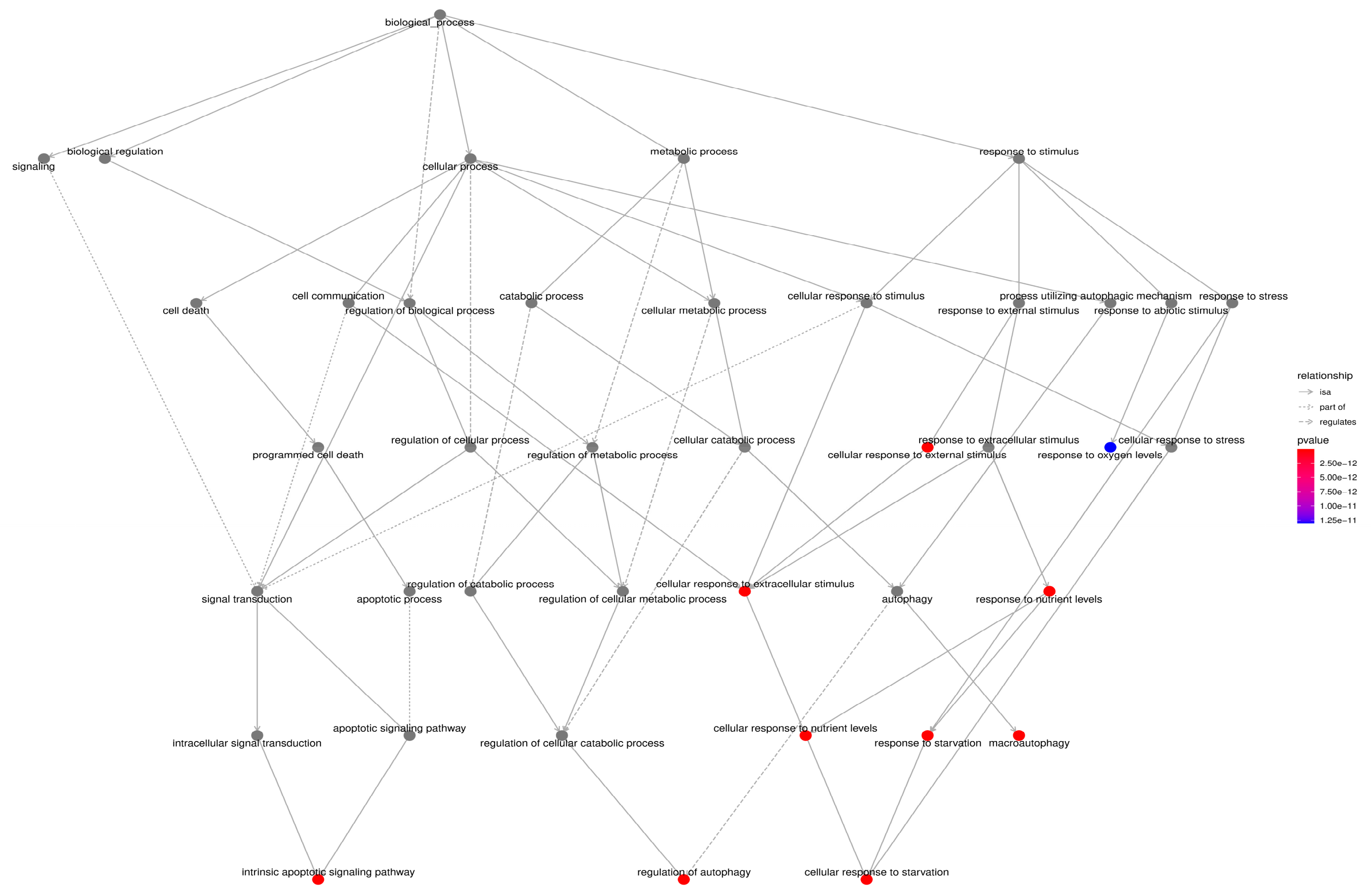

Figure S5. Go plot of enriched "Biological Process" (P< 0.05, FDR < 0.05). The plot is generated by SRPLOT(http://www.bioinformatics.com.cn/srplot) tool based on the output of enriched biological processes. P value - Benjamini–Hochberg adjusted P-value for the enriched ontology term. Size – number of DEGs belonging to enriched biological process. Colour- intensity indicates enriched biological processes.

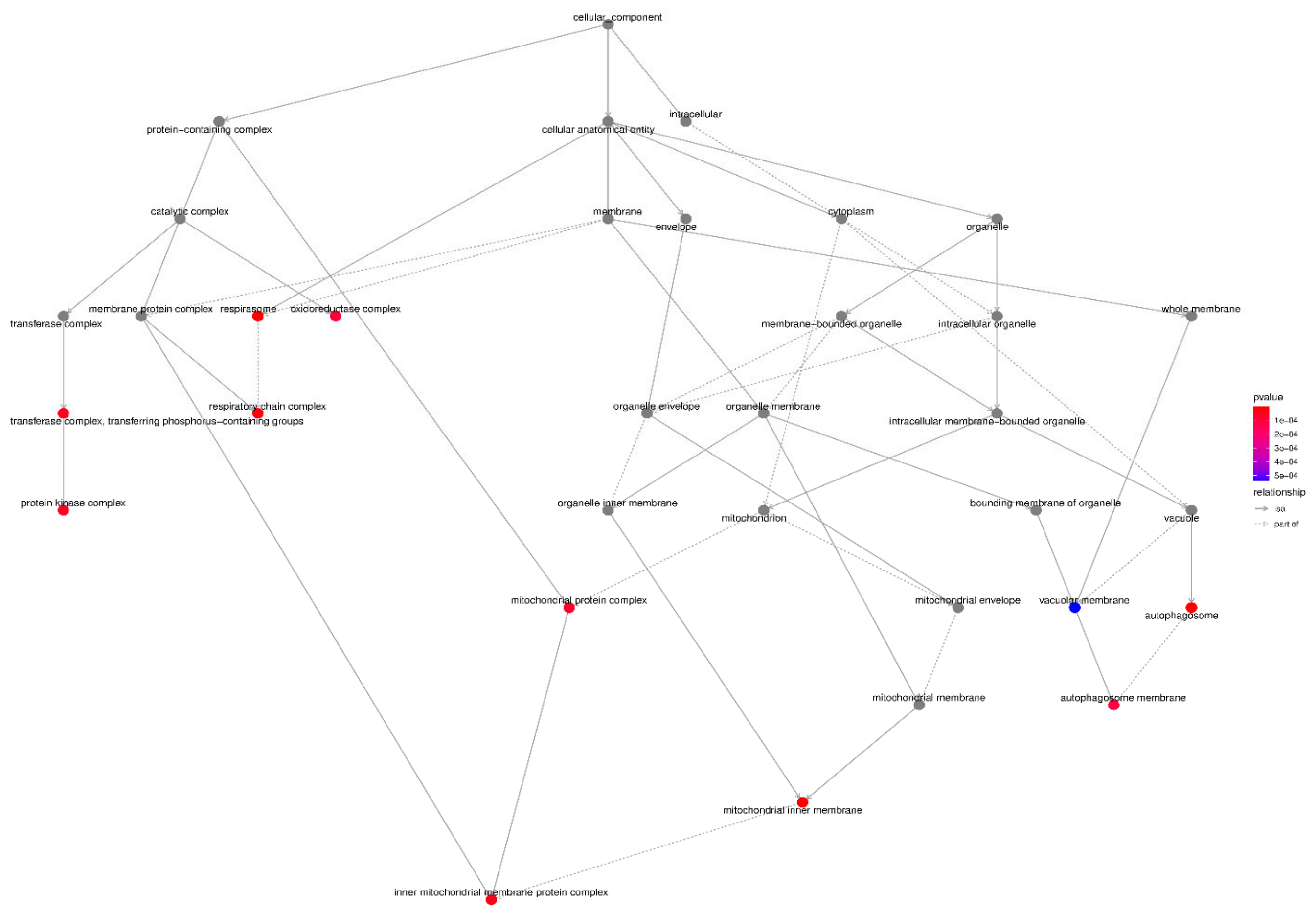

Figure S6. Go plot of enriched "Cellular components" (P< 0.05, FDR < 0.05). The plot is generated by SRPLOT(http://www.bioinformatics.com.cn/srplot) tool based on the output of KEGG analysis. P value - Benjamini–Hochberg adjusted P-value for the enriched ontology term. Size – number of DEGs belonging to enriched cellular components. Colour- intensity indicates enriched cellular components.

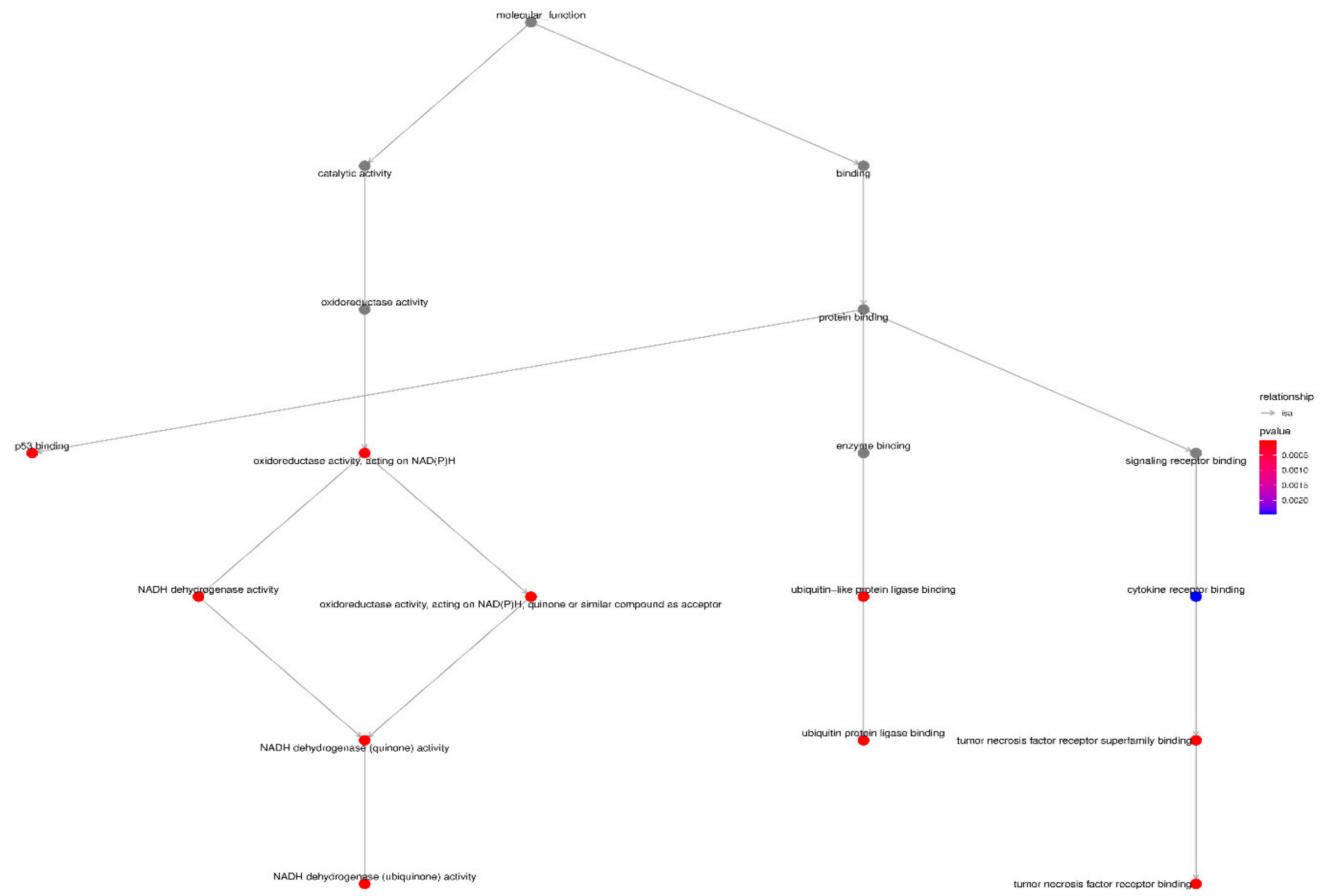

Figure S7. Go plot of enriched "Molecular function" (P< 0.05, FDR < 0.05). The plot is generated by SRPLOT(http://www.bioinformatics.com.cn/srplot) tool based on the output of KEGG analysis. P value - Benjamini–Hochberg adjusted P-value for the enriched ontology term. Size – number of DEGs belonging to enriched molecular functions. Colour- intensity indicates enriched molecular activities of DEGs.

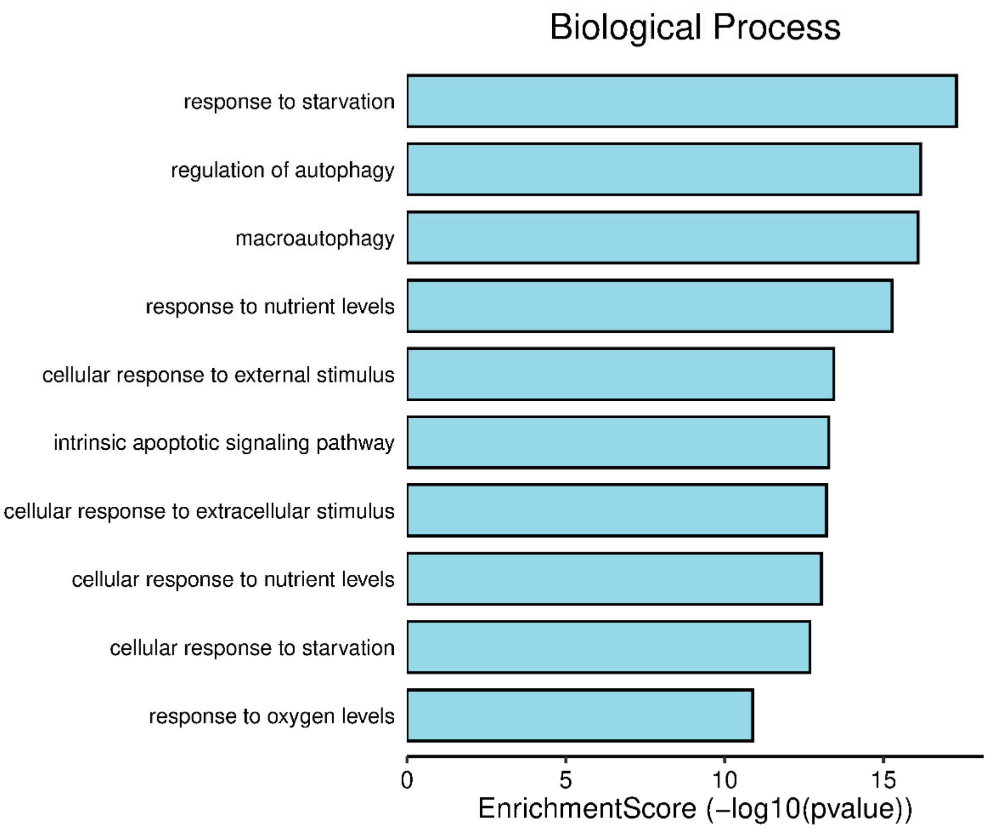

Figure S8. Enrichment Score plot of major "Biological Process" involved. The plot is generated by SRPLOT(http://www.bioinformatics.com.cn/srplot) tool based on the output of KEGG analysis.

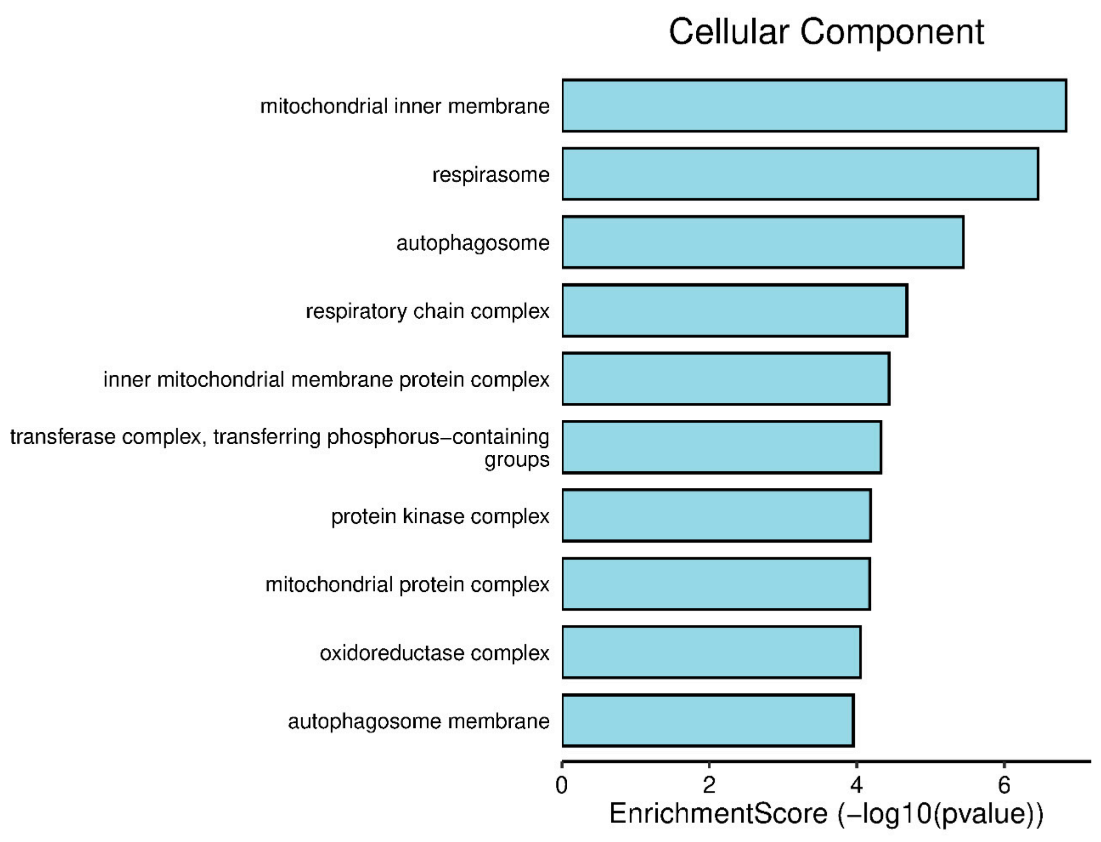

Figure S9. Enrichment Score plot of major "Cellular components" involved. The plot is generated by SRPLOT(http://www.bioinformatics.com.cn/srplot) tool based on the output of KEGG analysis.

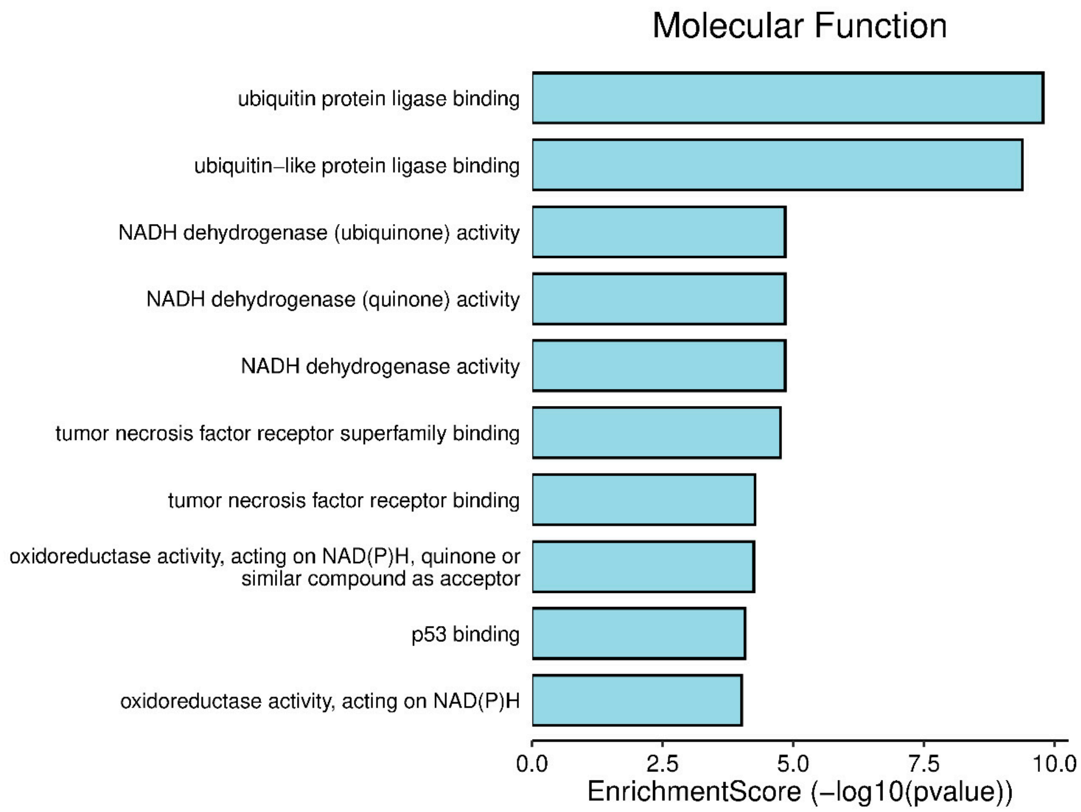

Figure S10. Enrichment Score plot of major "Molecular function" involved. The plot is generated by SRPLOT(http://www.bioinformatics.com.cn/srplot) tool based on the output of KEGG analysis.

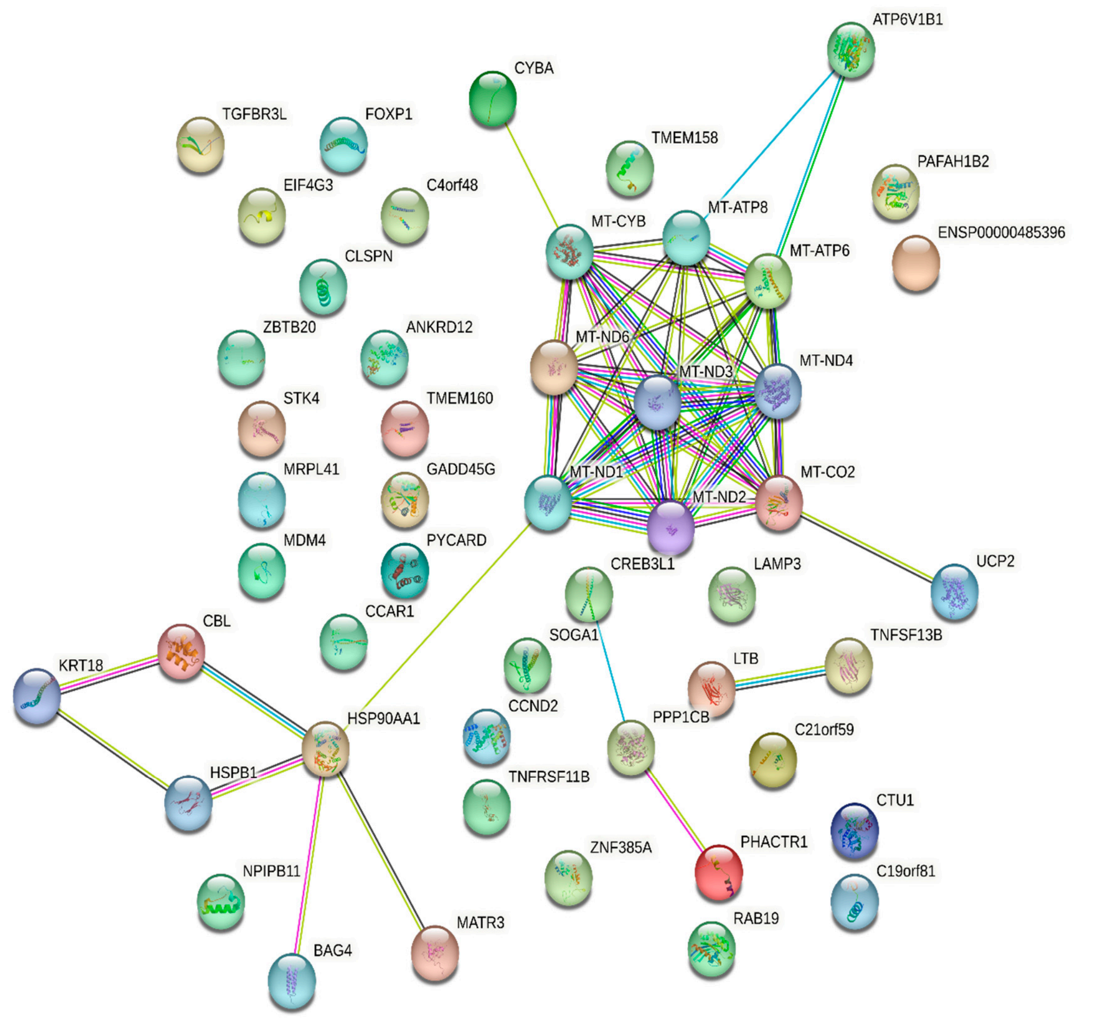

Figure S11. Protein-protein interaction network constructed using STRING (Search Tool for the Retrieval of Interacting Genes; version 11.5; https://string-db.org) with significantly downregulated DEGs in ACHN cell line response to starvation induced autophagy and cisplatin.

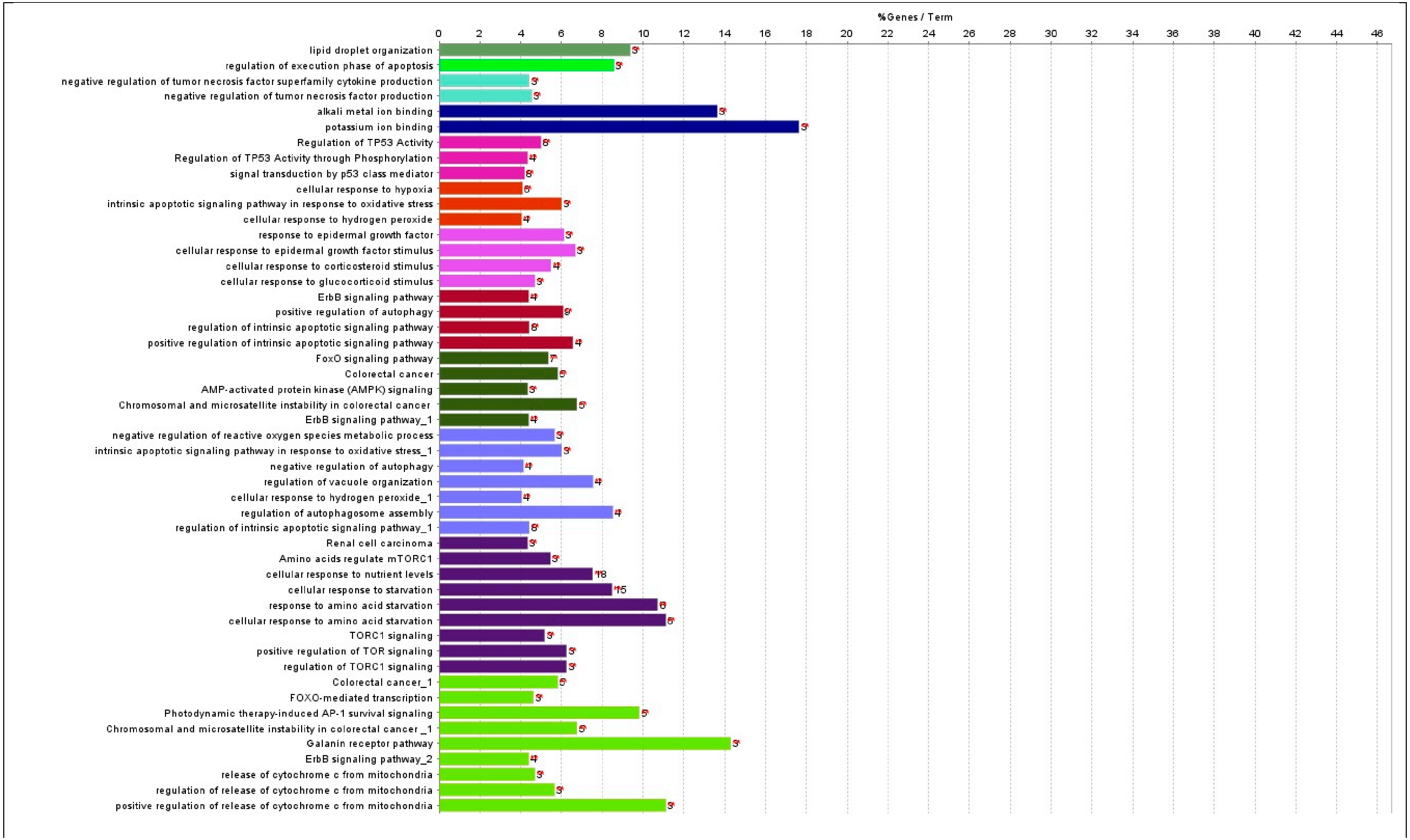

Bar plot continue

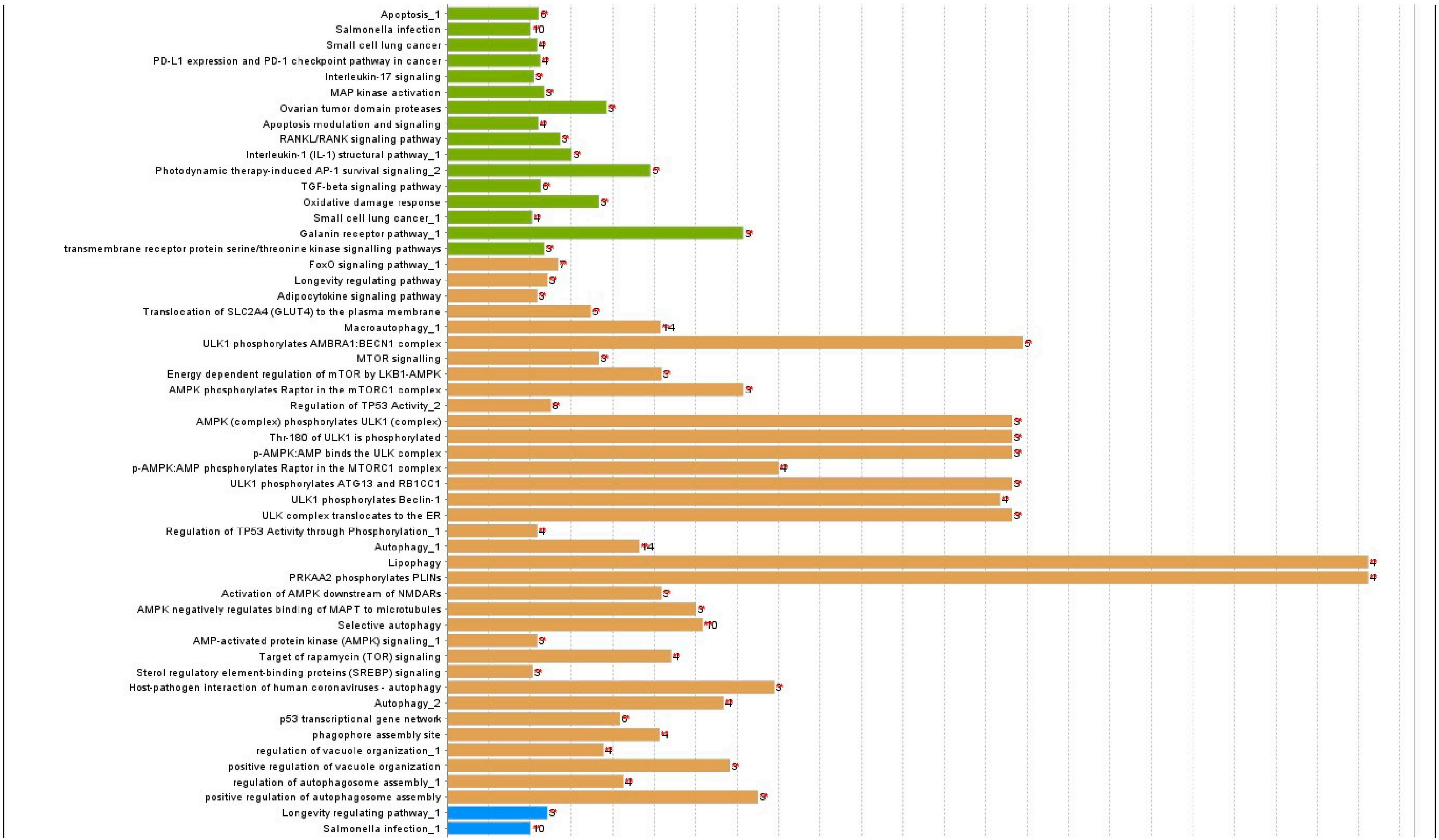

Bar plot continue

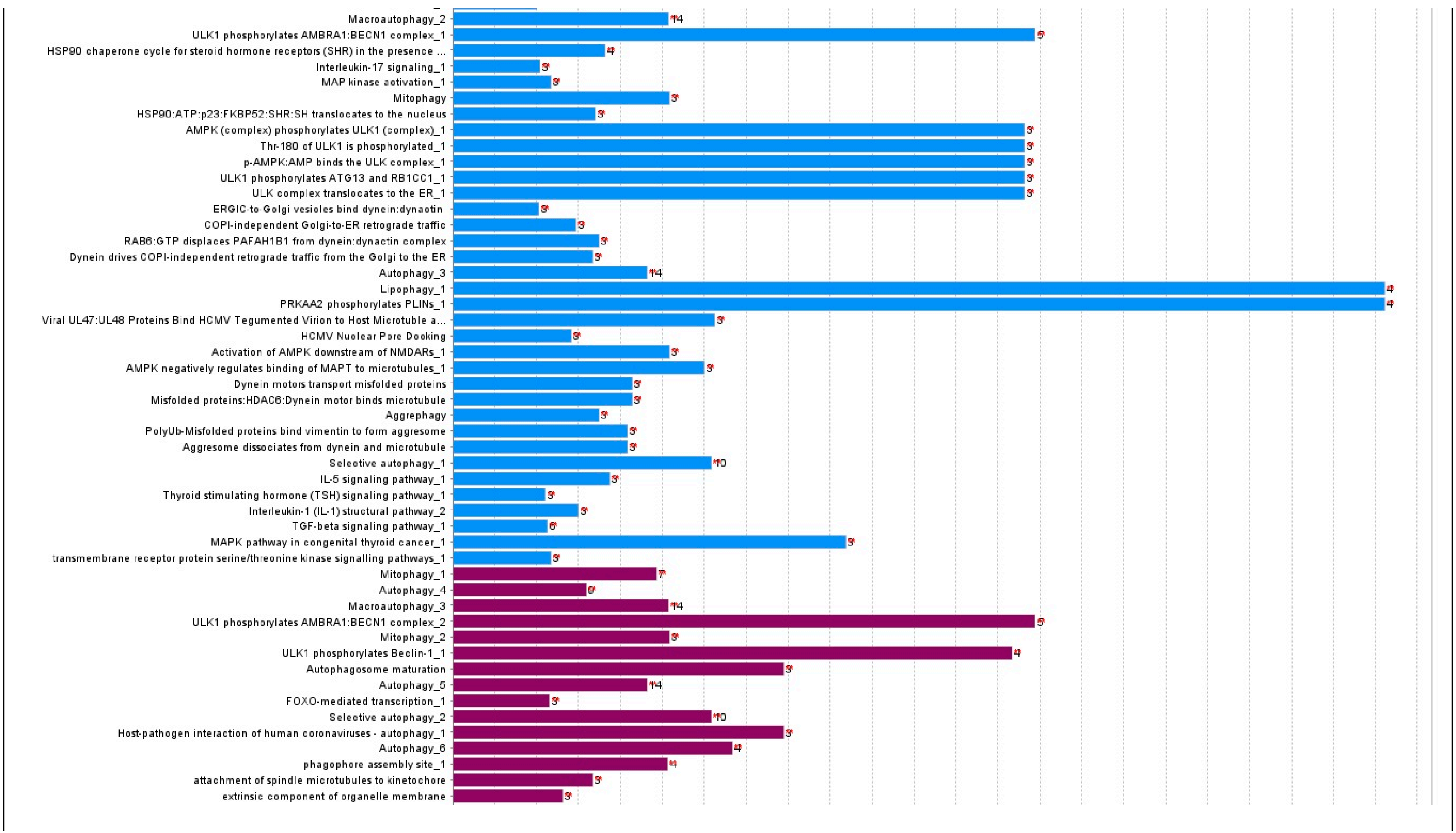

Bar plot continue

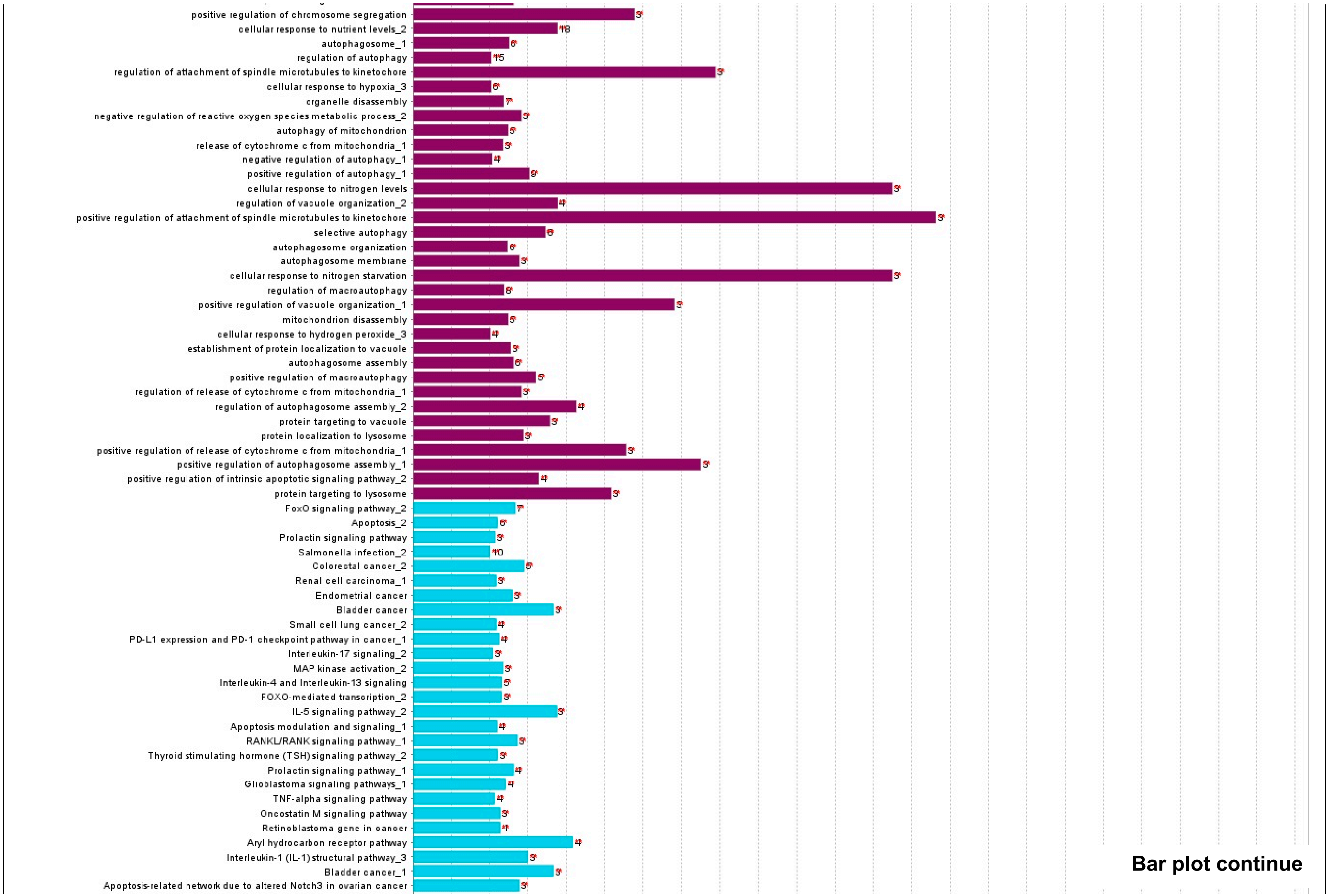
positive regulation of chromosome segregation
cellular response to nutrient levels_2
autophagosome_1
regulation of autophagy
regulation of attachment of spindle microtubules to kinetochore
cellular response to hypoxia_3
organelle disassembly
negative regulation of reactive oxygen species metabolic process_2
autophagy of mitochondrion
release of cytochrome c from mitochondria_1
negative regulation of autophagy_1
positive regulation of autophagy_1
cellular response to nitrogen levels
regulation of vacuole organization_2
positive regulation of attachment of spindle microtubules to kinetochore
selective autophagy
autophagosome organization
autophagosome membrane
cellular response to nitrogen starvation
regulation of macroautophagy
positive regulation of vacuole organization_1
mitochondrion disassembly
cellular response to hydrogen peroxide_3
establishment of protein localization to vacuole
autophagosome assembly
positive regulation of macroautophagy
regulation of release of cytochrome c from mitochondria_1
regulation of autophagosome assembly_2
protein targeting to vacuole
protein localization to lysosome
positive regulation of release of cytochrome c from mitochondria_1
positive regulation of autophagosome assembly_1
positive regulation of intrinsic apoptotic signaling pathway_2
protein targeting to lysosome
FoxO signaling pathway_2
Apoptosis_2
Prolactin signaling pathway
Salmonella infection_2
Colorectal cancer_2
Renal cell carcinoma_1
Endometrial cancer
Bladder cancer
Small cell lung cancer_2
PD-L1 expression and PD-1 checkpoint pathway in cancer_1
Interleukin-17 signaling_2
MAP kinase activation_2
Interleukin-4 and Interleukin-13 signaling
FOXO-mediated transcription_2
IL-5 signaling pathway_2
Apoptosis modulation and signaling_1
RANKL/RANK signaling pathway_1
Thyroid stimulating hormone (TSH) signaling pathway_2
Prolactin signaling pathway_1
Glioblastoma signaling pathways_1
TNF-alpha signaling pathway
Oncostatin M signaling pathway
Retinoblastoma gene in cancer
Aryl hydrocarbon receptor pathway
Interleukin-1 (IL-1) structural pathway_3
Bladder cancer_1
Apoptosis-related network due to altered Notch3 in ovarian cancer
Bar plot continue

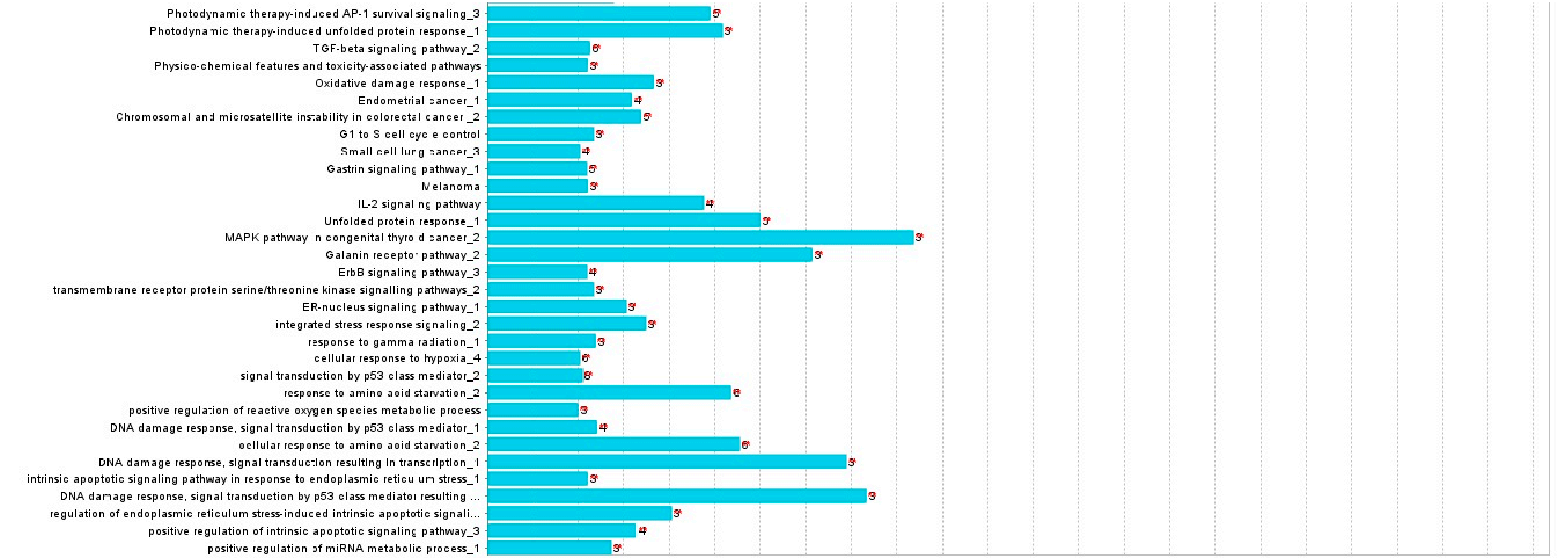

Figure S12. Enriched pathways in ACHN cell line response to starvation induced autophagy and cisplatin, constructed from all the upregulated PPI networks using Cytoscape plugin ClueGO (v3.9.1). The significantly enriched pathways are denoted by different colors.

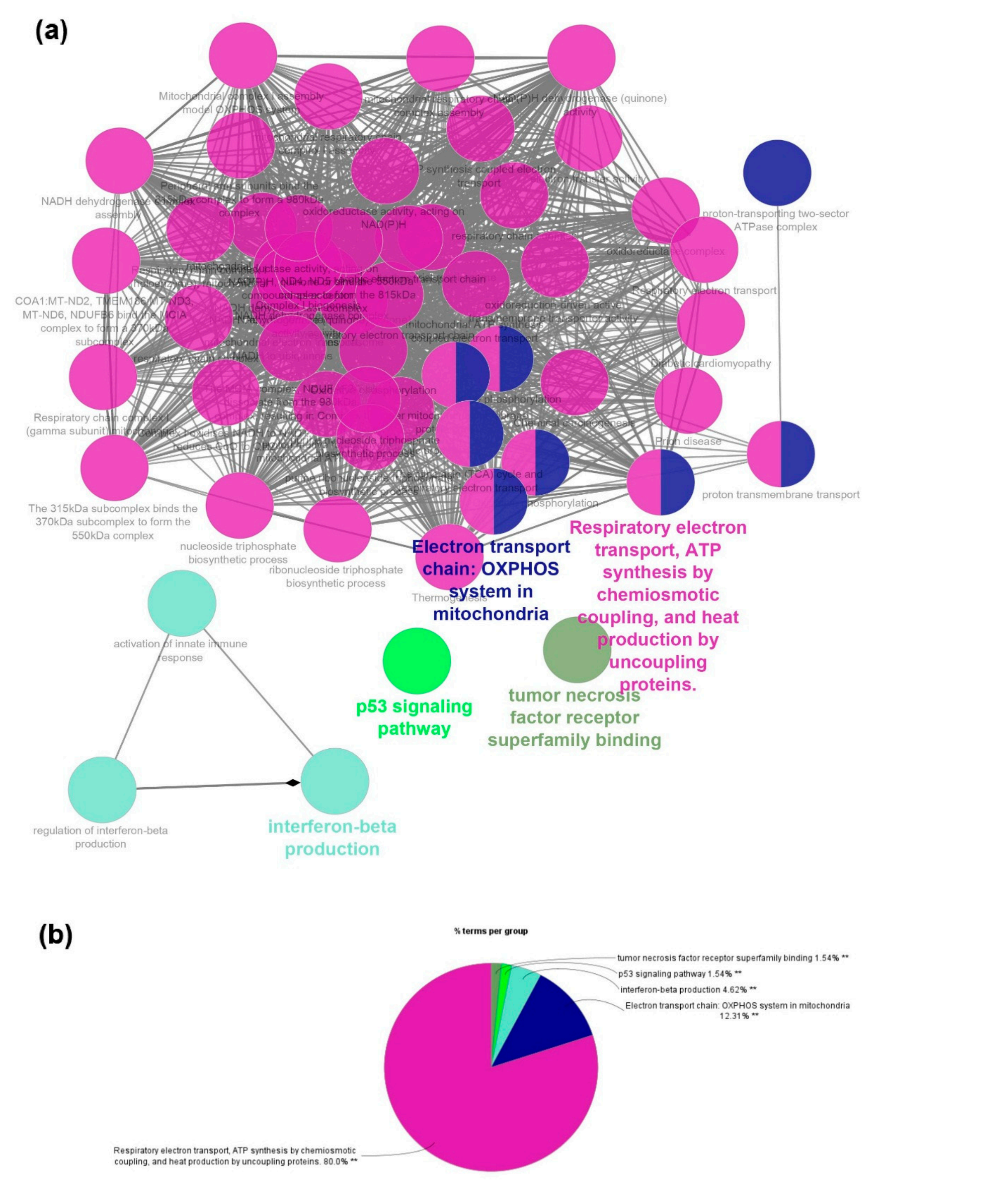

Figure S13. Pathway interactions in ACHN cell line response to starvation induced autophagy and cisplatin. Constructed pathway network from all the downregulated PPI networks using Cytoscape plugin ClueGO (v3.9.1). **(a)** The significantly enriched pathways were denoted by different colours. **(b)** Pie chart shows the overrepresented functional categories of the ClueGO pathway analysis (** $p < 0.001$, * $p < 0.01$, without star $p < 0.05$).

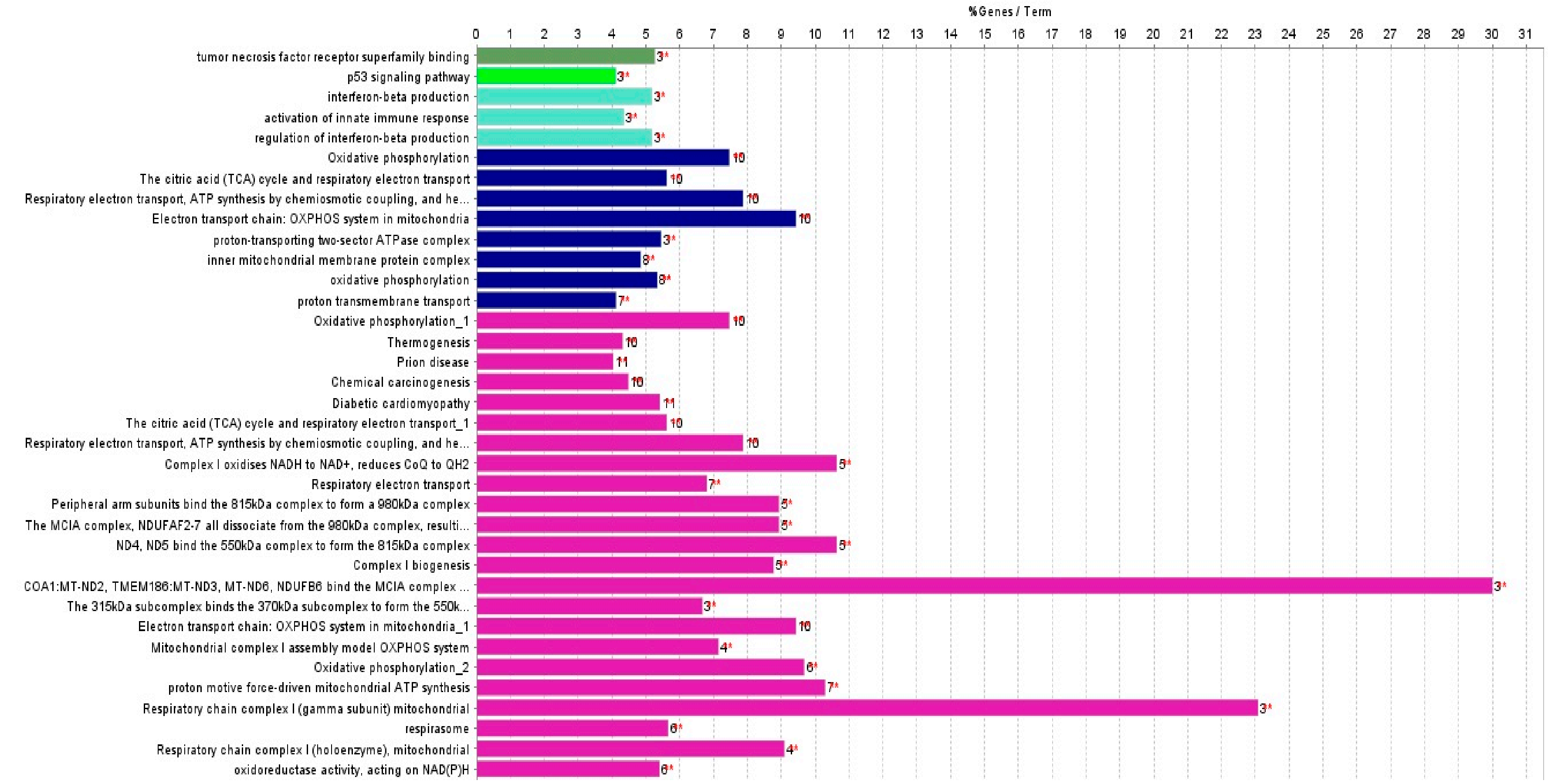

Bar plot continue

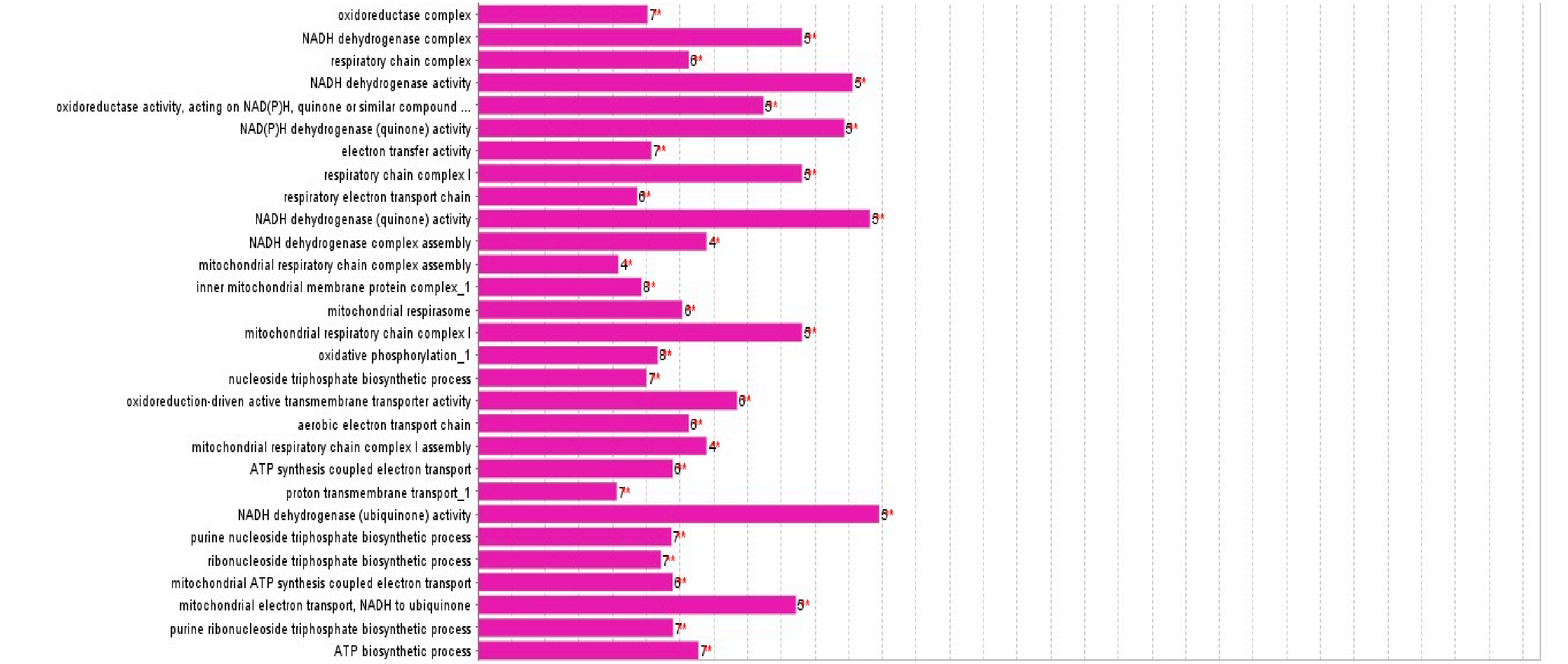

Figure S14. Enriched pathways in ACHN cell line response to starvation induced autophagy and cisplatin, constructed from all the downregulated PPI networks using Cytoscape plugin ClueGO(v3.9.1). The significantly enriched pathways are denoted by different colors.

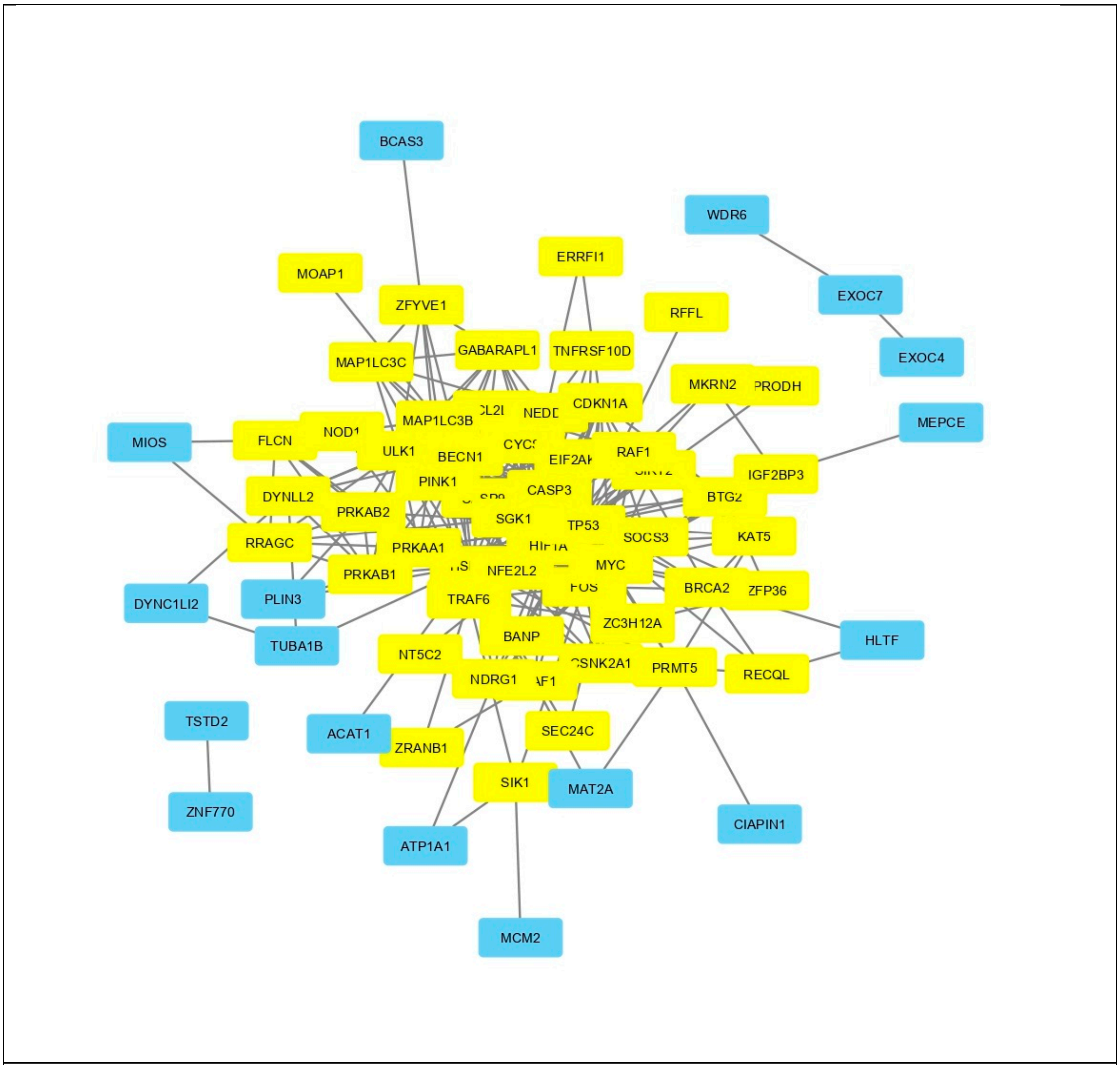

Figure S15. MCODE Cluster 1 generated with all the upregulated DEGs using Cytoscape plugin MCODE. Cluster 1 forms with 54 nodes and 272 edges with a cluster score of 10.56.

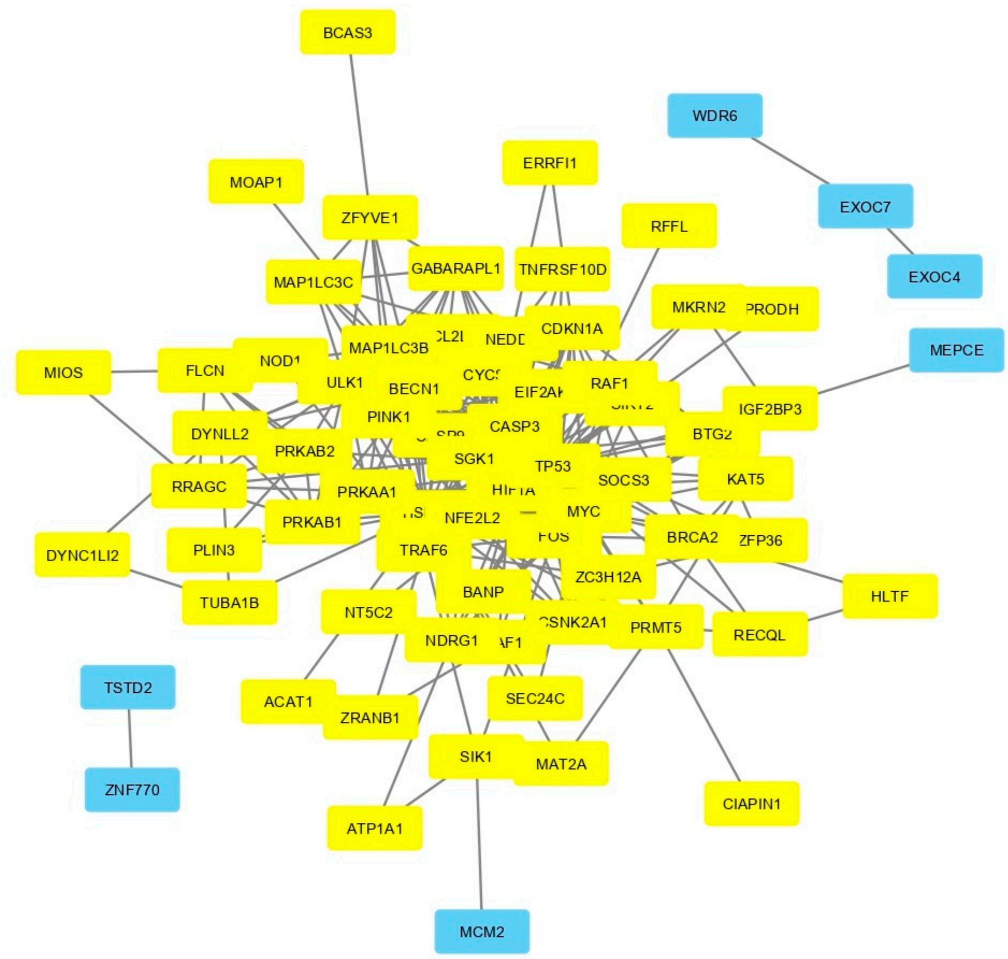

Figure S16. MCODE Cluster 2 generated with all the upregulated DEGs using Cytoscape plugin MCODE. Cluster 2 forms with 64 nodes and 290 edges with a cluster score of 6.73.

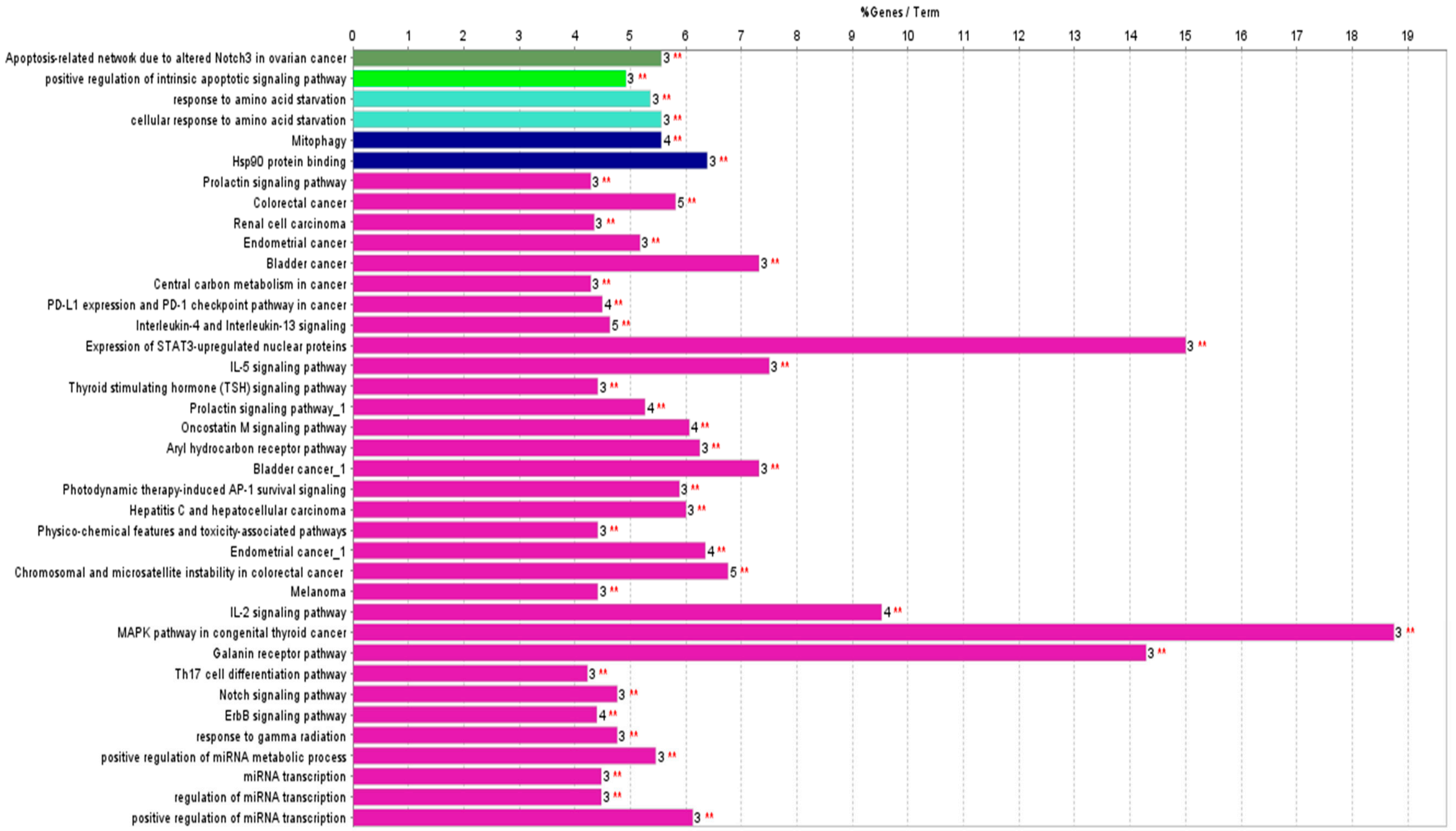

Figure S17. Enriched pathways in ACHN cell line response to starvation induced autophagy and cisplatin, constructed from cluster - 1 using Cytoscape plugin ClueGO (v3.9.1).

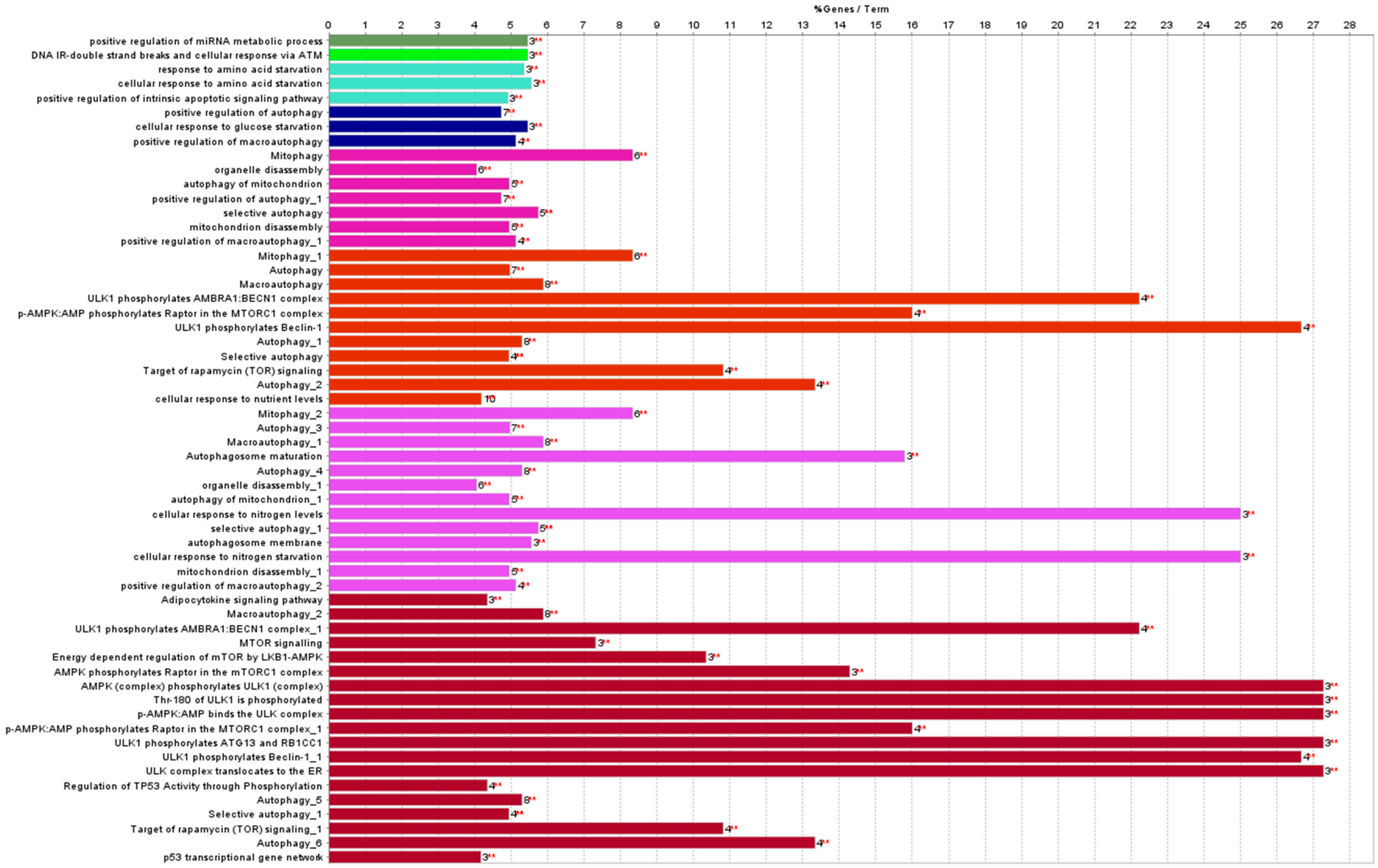

Figure S18. Enriched pathways in ACHN cell line response to starvation induced autophagy and cisplatin, constructed from cluster - 2 using Cytoscape plugin ClueGO (v3.9.1).

(A)

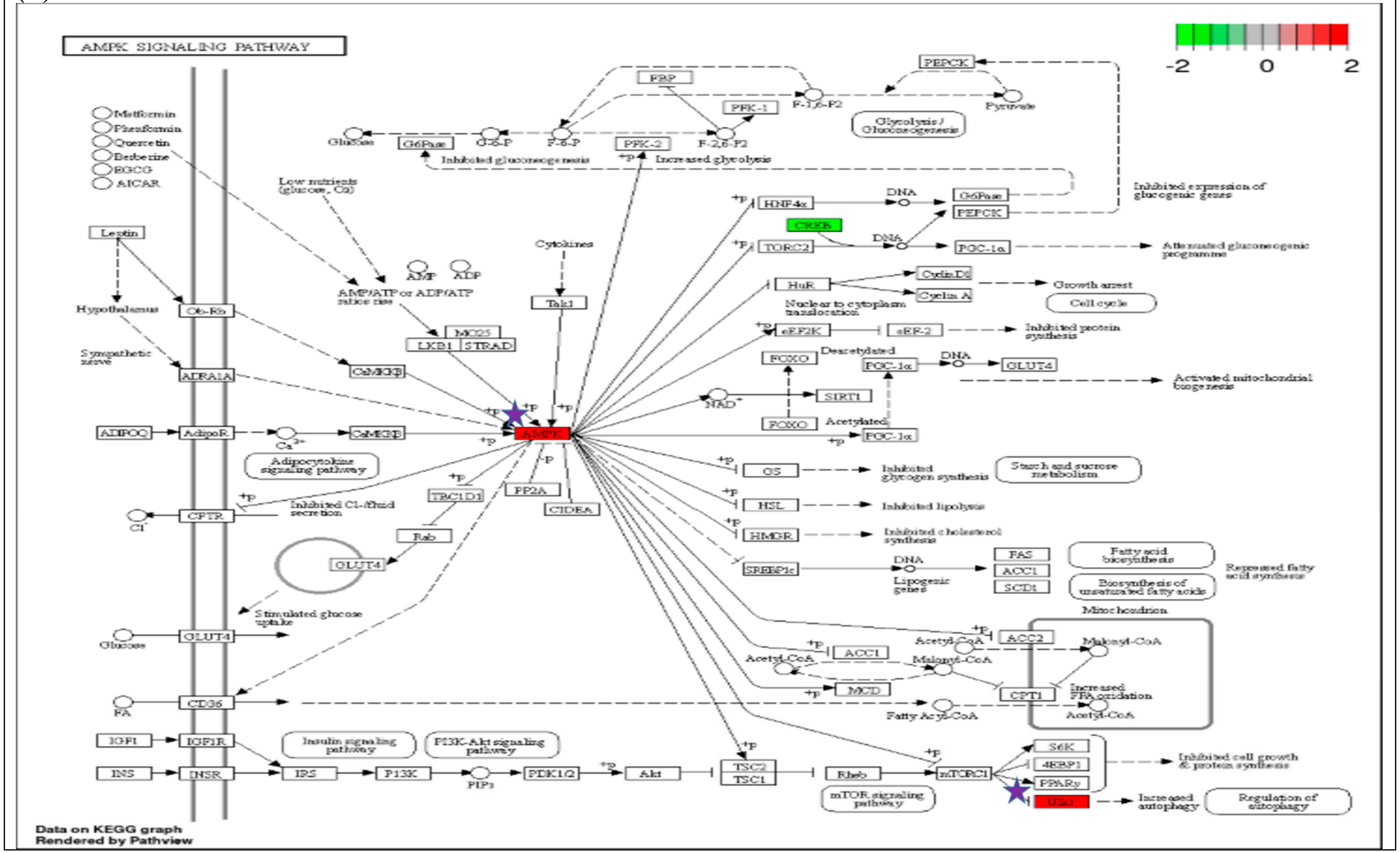

AMPK SIGNALING PATHWAY
-2
0
2
Metformin
Phenformin
Quercetin
Berberine
EGCG
AICAR
Leptin
Ob-Rb
Hypothalamus
Sympathetic nerve
ADRA1A
ADIPOQ
AdipoR
CaMKKβ
Adipocytokine signaling pathway
CFTR
Inhibited Cl-/fluid secretion
GLUT4
Stimulated glucose uptake
Glucose
CD36
FA
IGF1
IGF1R
INS
INSR
IRS
PI3K
PIP3
PDK1/2
Akt
Insulin signaling pathway
PI3K-Akt signaling pathway
Low nutrients (glucose, O2)
AMP/ATP or ADP/ATP ratios rise
AMP
ADP
MO25
LKB1
STRAD
Cytokines
Tak1
AMPK
PP2A
CIDEA
TBC1D1
Rab
Glucose
G6Pase
G-6-P
F-6-P
FBP
PFK-1
PFK-2
F-2,6-P2
F-1,6-P2
PEPCK
Pyruvate
Glycolysis / Gluconeogenesis
Inhibited gluconeogenesis
Increased glycolysis
HNF4α
CREB
TORC2
DNA
G6Pase
PEPCK
PGC-1α
Inhibited expression of glucogenic genes
Attenuated gluconeogenic programme
HuR
Cyclin D1
Cyclin A
Growth arrest
Cell cycle
Nuclear to cytoplasm translocation
eEF2K
eEF-2
Inhibited protein synthesis
FOXO
Deacetylated
GLUT4
Activated mitochondrial biogenesis
NAD+
SIRT1
Acetylated
GS
Inhibited glycogen synthesis
Starch and sucrose metabolism
HSL
Inhibited lipolysis
HMGR
Inhibited cholesterol synthesis
SREBP1c
Lipogenic genes
FAS
ACC1
SCD1
Fatty acid biosynthesis
Biosynthesis of unsaturated fatty acids
Repressed fatty acid synthesis
Mitochondrion
ACC2
Acetyl-CoA
Malonyl-CoA
ACC1
MCD
CPT1
Increased FFA oxidation
Fatty Acyl-CoA
TSC2
TSC1
Rheb
mTORC1
mTOR signaling pathway
S6K
4EBP1
PPARγ
ULK1
Inhibited cell growth & protein synthesis
Increased autophagy
Regulation of autophagy
Data on KEGG graph
Rendered by Pathview

(B)

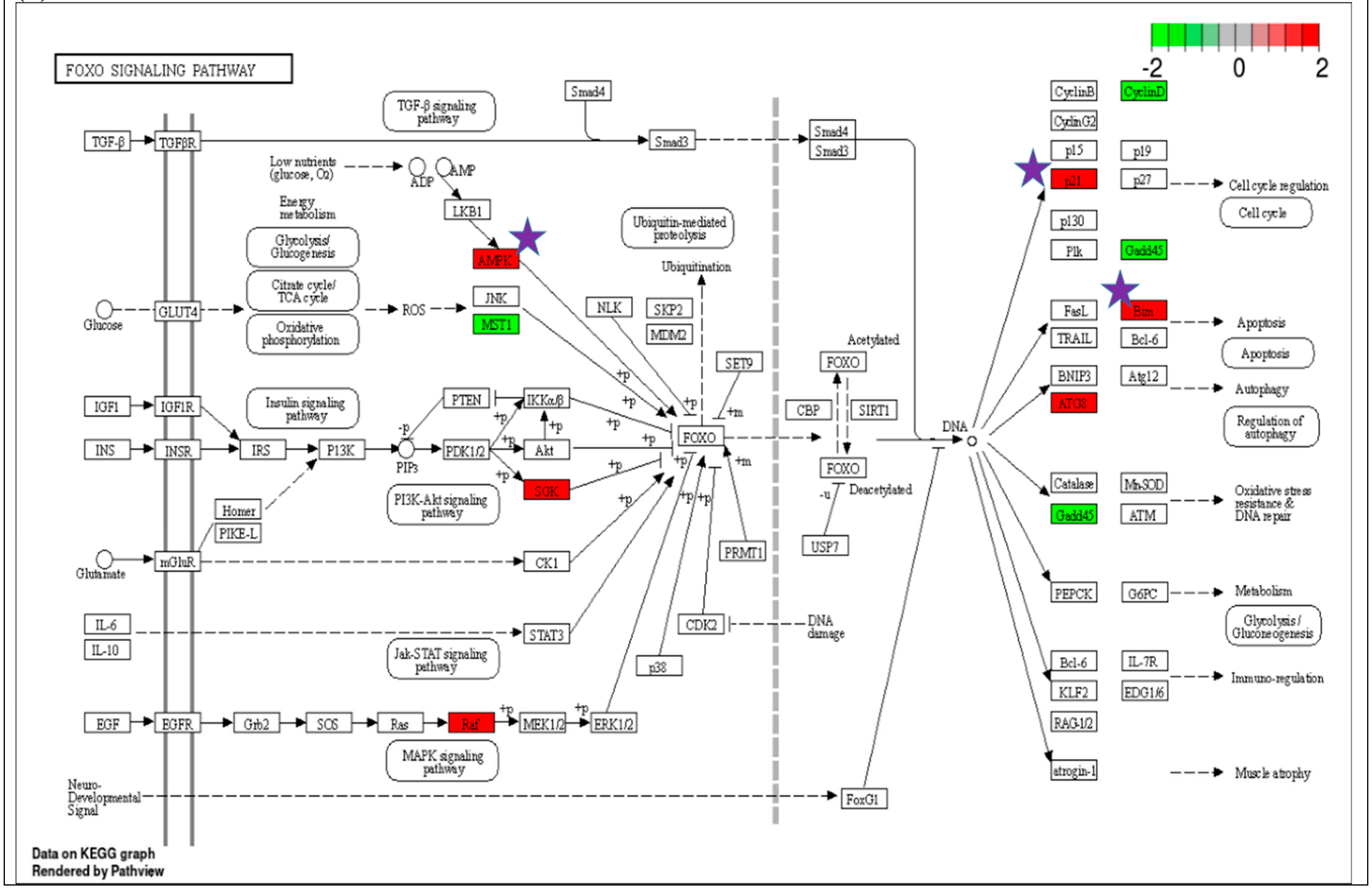
FOXO SIGNALING PATHWAY
-2
0
2
TGF-β
TGFβR
TGF-β signaling pathway
Smad4
Smad3
Low nutrients (glucose, O2)
ADP
AMP
Energy metabolism
LKB1
AMPK
Glycolysis/ Gluconeogenesis
Citrate cycle/ TCA cycle
Oxidative phosphorylation
Glucose
GLUT4
ROS
JNK
MST1
Ubiquitin-mediated proteolysis
Ubiquitination
NLK
SKP2
MDM2
SET9
IGF1
IGF1R
INS
INSR
IRS
PI3K
PIP3
PTEN
IKKα/β
PDK1/2
Akt
SGK
Insulin signaling pathway
PI3K-Akt signaling pathway
Homer
PIKE-L
Glutamate
mGluR
CK1
IL-6
IL-10
STAT3
Jak-STAT signaling pathway
EGF
EGFR
Grb2
SOS
Ras
Raf
MEK1/2
ERK1/2
MAPK signaling pathway
p38
CDK2
PRMT1
DNA damage
FOXO
Acetylated
CBP
SIRT1
Deacetylated
USP7
Neuro-Developmental Signal
FoxG1
DNA
CyclinB
CyclinD
CyclinG2
p15
p19
p21
p27
p130
Plk
Gadd45
Cell cycle regulation
Cell cycle
FasL
Bim
TRAIL
Bcl-6
BNIP3
Atg12
ATG8
Apoptosis
Autophagy
Regulation of autophagy
Catalase
MnSOD
ATM
Oxidative stress resistance & DNA repair
PEPCK
G6PC
Metabolism
Glycolysis / Gluconeogenesis
IL-7R
KLF2
EDG1/6
RAG-1/2
Immuno-regulation
atrogin-1
Muscle atrophy
Data on KEGG graph
Rendered by Pathview

(C)

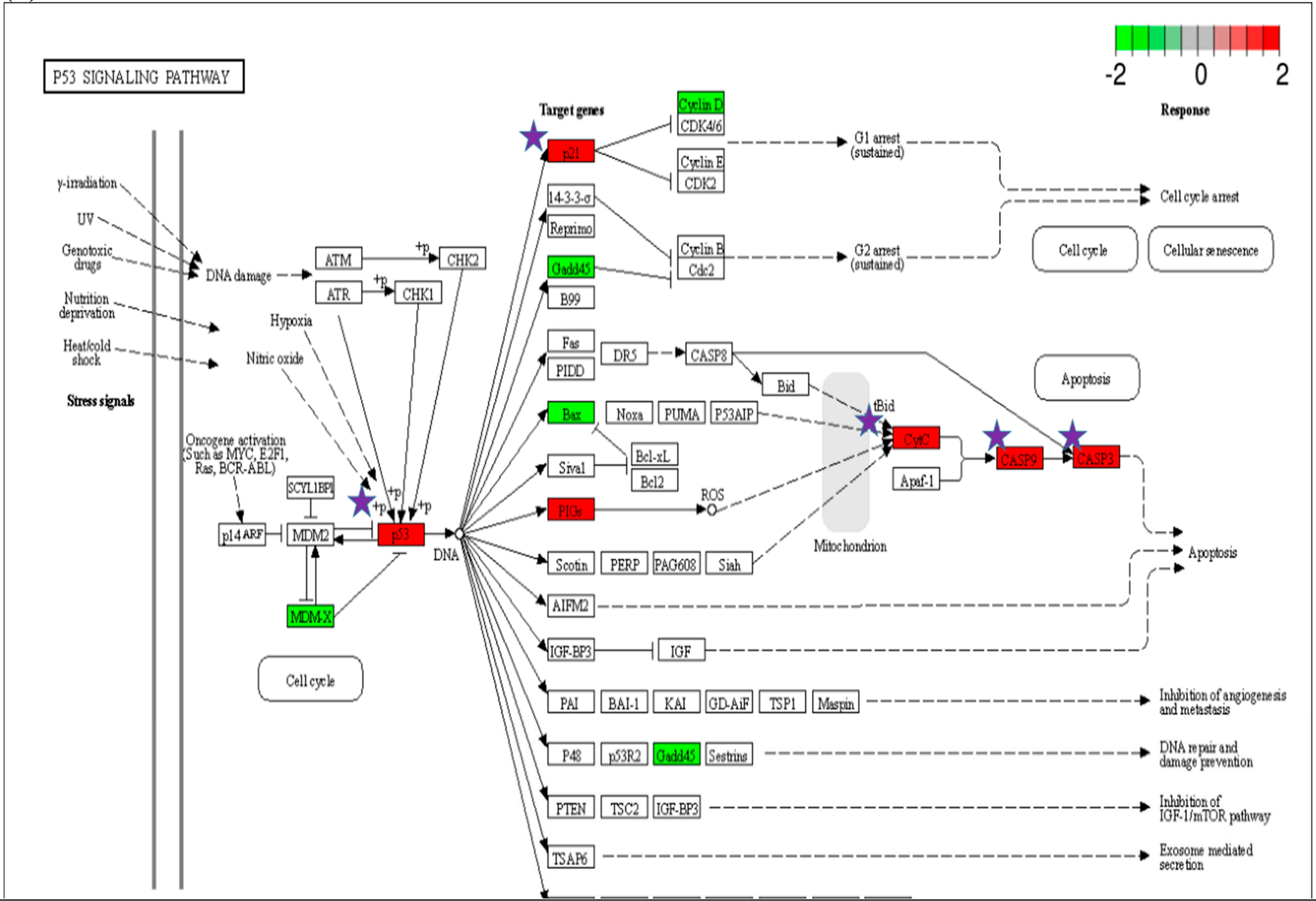

P53 SIGNALING PATHWAY
-2
0
2
Response
Target genes
Cyclin D
CDK4/6
Cyclin E
CDK2
G1 arrest (sustained)
Cell cycle arrest
14-3-3-σ
Reprimo
Cyclin B
Cdc2
G2 arrest (sustained)
Gadd45
B99
Cell cycle
Cellular senescence
γ-irradiation
UV
Genotoxic drugs
DNA damage
ATM
CHK2
ATR
CHK1
+p
Nutrition deprivation
Hypoxia
Heat/cold shock
Nitric oxide
Stress signals
Fas
PIDD
DR5
CASP8
Bid
Apoptosis
Bax
Noxa
PUMA
P53AIP
tBid
CytC
CASP9
CASP3
Apaf-1
Bcl-xL
Bcl2
Sival
ROS
PIGs
Oncogene activation (Such as MYC, E2F1, Ras, BCR-ABL)
SCYL1BP1
p14 ARF
MDM2
p53
DNA
MDM-X
Scotin
PERP
PAG608
Siah
Mitochondrion
AIFM2
IGF-BP3
IGF
Cell cycle
PAI
BAI-1
KAI
GD-AiF
TSP1
Maspin
Inhibition of angiogenesis and metastasis
P48
p53R2
Sestrins
DNA repair and damage prevention
PTEN
TSC2
Inhibition of IGF-1/mTOR pathway
TSAP6
Exosome mediated secretion

(D)

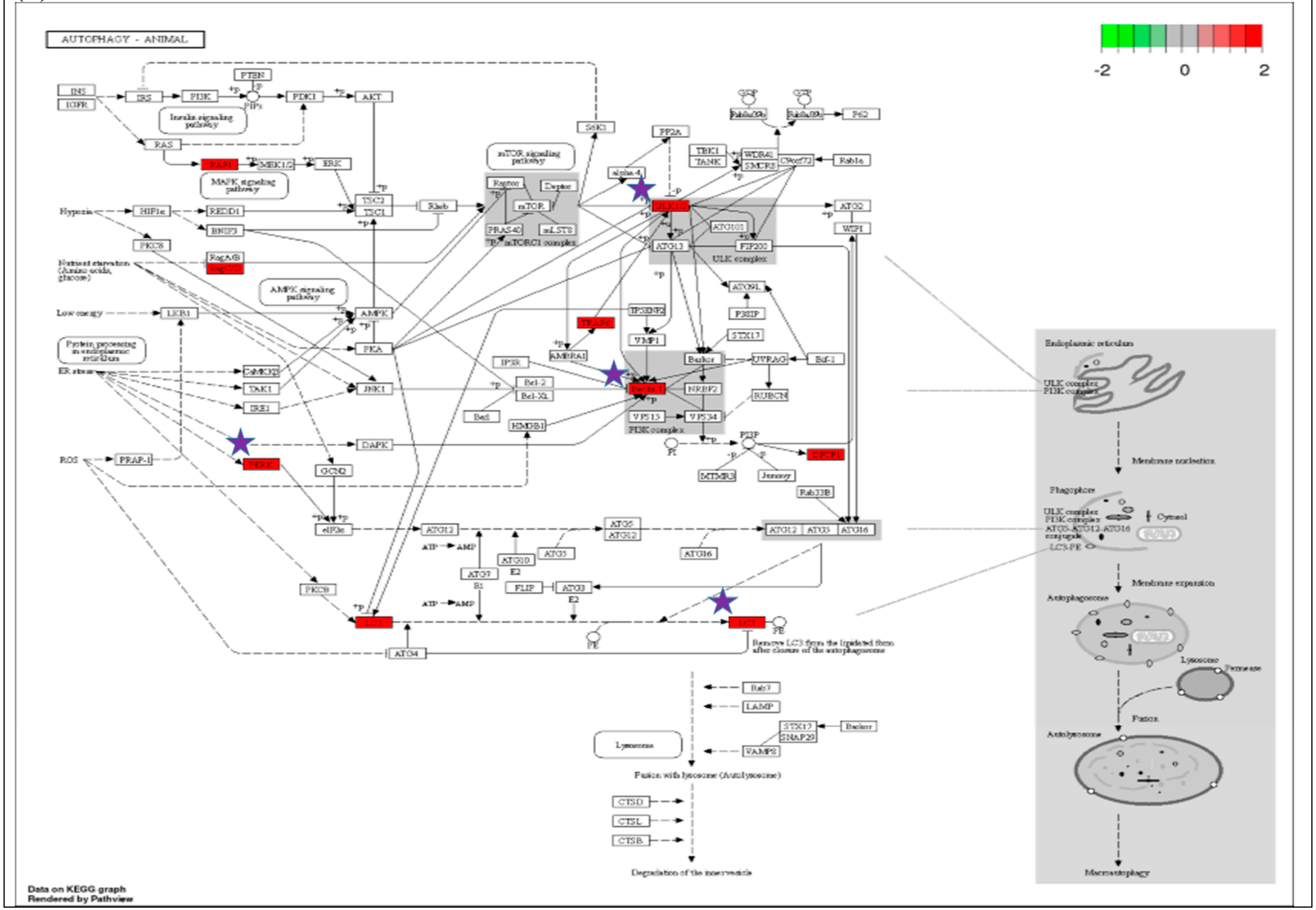
AUTOPHAGY - ANIMAL
-2
0
2
INS
IGFR
IRS
PI3K
PTEN
PDK1
AKT
RAS
RAP1
MEK1/2
ERK
Insulin signaling pathway
MAPK signaling pathway
mTOR signaling pathway
Hypoxia
HIF1α
REDD1
BNIP3
TSC2
TSC1
Rheb
Raptor
Deptor
mTOR
PRAS40
mLST8
mTORC1 complex
S6K1
PP2A
alpha-4
ULK1/2
ATG13
ATG101
FIP200
ULK complex
TBK1
TANK
WDR41
SMCR8
C9orf72
Rab1a
P62
ATG2
WIPI
ATG9L
P38IP
STX17
Nutrient starvation (Amino acids, glucose)
RagA/B
RagC/D
PKCδ
AMPK signaling pathway
Low energy
LKB1
AMPK
PKA
Protein processing in endoplasmic reticulum
ER stress
CaMKK2
TAK1
IRE1
JNK1
IP3R
Bcl-2
Bcl-XL
Bad
HMGB1
TRAF6
AMBRA1
VMP1
Beclin1
NRBF2
UVRAG
Bif-1
RUBCN
VPS15
VPS34
PI3K complex
PI
PI3P
DFCP1
MTMR3
Jumpy
Rab33B
ROS
PRAP-1
PERK
DAPK
GCN2
eIF2α
ATG12
ATG5
ATG10
ATG7
ATG3
ATG16
FLIP
PKCθ
LC3
ATG4
PE
ATP
AMP
E1
E2
Remove LC3 from the lipidated form after closure of the autophagosome
Rab7
LAMP
STX17
SNAP29
VAMP8
Lysosome
Fusion with lysosome (Autolysosome)
CTSD
CTSL
CTSB
Degradation of the inner vesicle
Endoplasmic reticulum
ULK complex
PI3K complex
Membrane nucleation
Phagophore
ATG5-ATG12-ATG16 conjugate
LC3-PE
Cytosol
Membrane expansion
Autophagosome
Lysosome
Permease
Fusion
Autolysosome
Macroautophagy
Data on KEGG graph
Rendered by Pathview

(**E**)

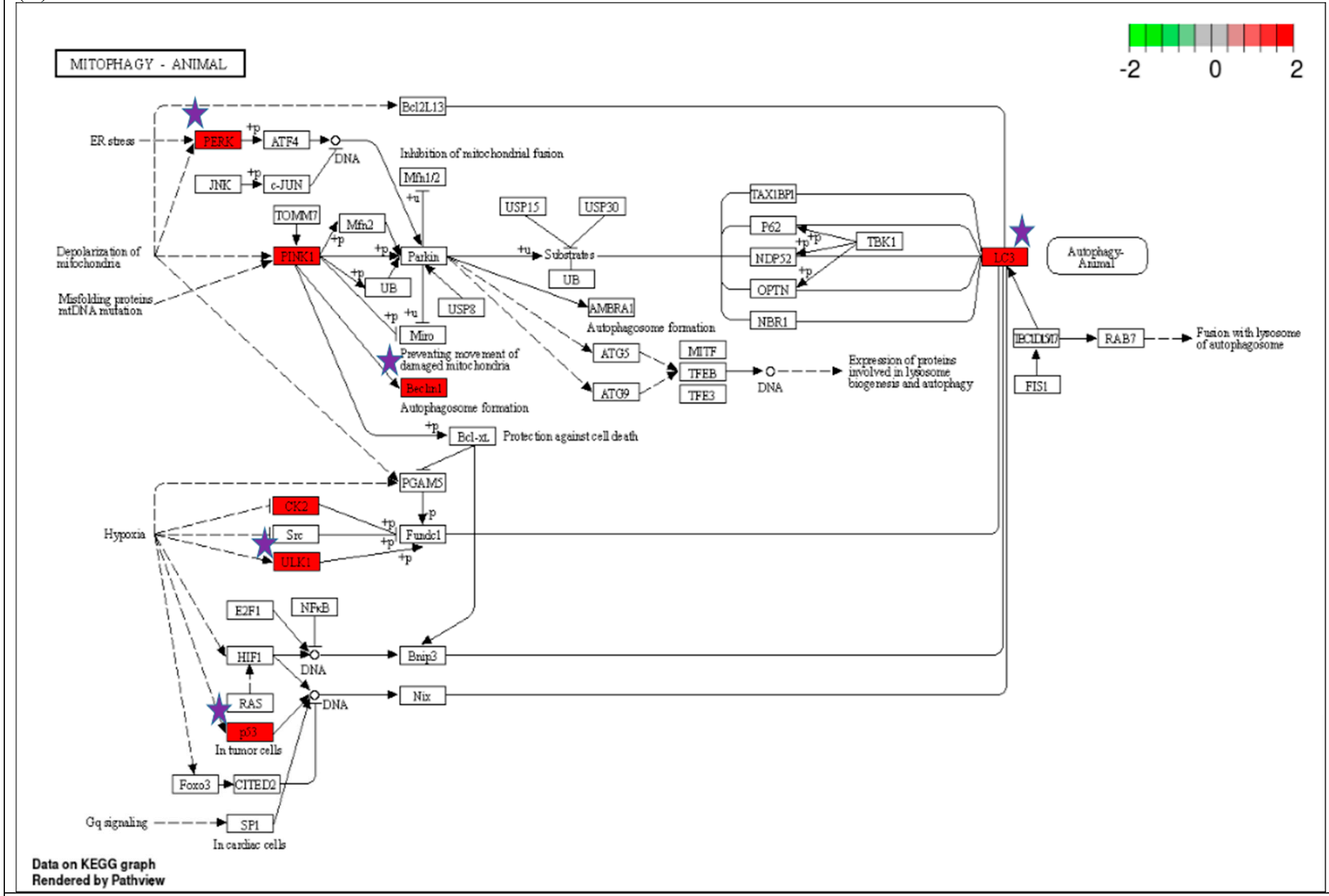

Figure S19. Hub gene identification by KEGG pathway viewer by analyzing upregulated DEGs. (**A**)AMPK signalling pathway, (**B**) FOXO signalling pathway, (**C**) p53 signalling pathway, (**D**)autophgic pathway (including mitophagy), and (**E**) apoptosis pathway

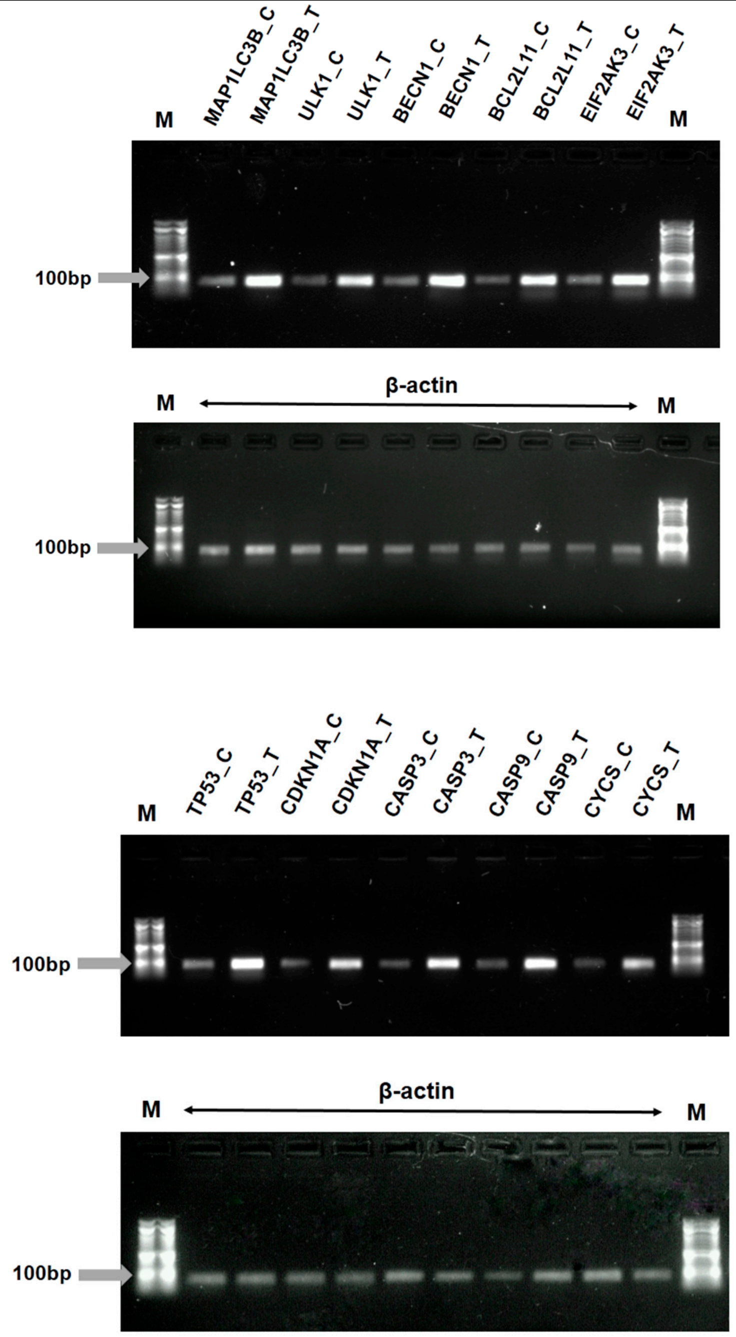

Figure S20. Validation of transcriptomic data by qRT-PCR amplification of different identified HUB genes. M-100 bp DNA ladder (Takara), gene_C - Control (autophagic ACHN cells without treatment), gene_T - Treated (Cisplatin-treated autophagic ACHN cells).

Supplementary Tables

Table S1. Quantification of RNA from autophagic ACHN (control-AT1) and cisplatin-treated autophaic ACHN (treated- AT2) cell lines. Quantification was done at 260/280nm by NanoDrop plate reader (BMG Labteck, Germany).

Sample	Quantified RNA (ng/µl)	OD $A_{260/280}$	OD $A_{260/230}$
**AT-1 (Control)**	512.3	2.03	2.28
**AT-2 (Treated)**	495.6	2.05	2.19

Table S2. Details of primers used in qRT-PCR for validation of RNAseq results.

GENE	Primer ID	Sequence	Start	Stop	Length (base)	Tm (^0C)	GC %	Amplicon Length
MAP1LC3B	MAP1LC3BF	GTTACGGAAAGCAGCAGTGTA	1265	1286	21	63.0	47.6	102
	MAP1LC3BR	CAGAAGGGAGTGTGTCTGAATG	1345	1367	22	62.0	50	
ULK1	ULK1F	CCACTGTGCTTGGACTTAGAA	4961	4982	21	62.0	47.6	113
	ULK1R	AGTCGGCCATGAAGCAATAA	5054	5074	20	62.0	45	
BECN1	BECN1F	CTACAGGATGGATGTGGAGAAAG	1179	1202	23	62.0	47.8	98
	BECN1R	TCCACTGCTCCTCAGAGTTA	1257	1277	20	62.0	50	
BCL2L11	BCL2L11F	CTGCTGGACACACACATACA	2793	2813	20	62.0	50	116
	BCL2L11R	GGGCTGAGGAAACAGAGTAAA	2888	2909	21	62.0	47.6	
EIF2AK3	EIF2AK3F	GGAGGACAAGTAGCACAAACT	3713	3734	21	62.0	47.6	107
	EIF2AK3R	GTAAACCCACCCTAGAACCATC	3798	3820	22	62.0	50	
TP53	TP53F	AGGGATGTTTGGGAGATGTAAG	1565	1587	22	62.0	45.5	99
	TP53R	CCTGGTTAGTACGGTGAAGTG	1643	1664	21	62.0	52.4	
CDKN1A	CDKN1AF	CGGAACAAGGAGTCAGACATT	1749	1770	21	62.0	47.6	105
	CDKN1AR	AGTGCCAGGAAAGACAACTAC	1833	1854	21	62.0	47.6	
CASP3	CASP3F	GCTGCCTGTAACTTGAGAGTAG	1979	2001	22	62.0	50	117

	CASP3R	GTATGGAGAAATGGGCTGTAGG	2074	2096	22	62.0	50	
CASP9	CAS9F	CACAGGGTCTGCTCTTTCTC	1376	1396	20	62.0	55	100
	CAS9R	CATTCATCTGTCCCTCTTCCTC	1454	1476	22	62.0	50	
CYCS	CYCSF	TGGCTAGTTGTGGCGTTTAG	2132	2152	20	62.0	50	115
	CYCSR	GCTTGAGCCTGGGAAATAGAG	2226	2247	21	62.0	52.4	
ß-actin	ACTBF	GGACCTGACTGACTACCTCAT	633	654	21	62.0	52.4	107
	ACTBR	CGTAGCACAGCTTCTCCTTAAT	718	740	22	62.0	45.5	

Table S3. Sequencing statistics obtained from IlluminaHiseq 4000 paired end sequencing. Where, AT-1= control (autophagic ACHN) and AT-2 = treated (cisplatin-treated autophaic ACHN).

Sample	No. PE reads	Total No. of bases	Data in Gb	GC%
**AT-1 (Control)**	1,08,78,317	3,285,251,734	3.28	48.48
**AT-2 (Treated)**	1,23,87,553	3,741,041,006	3.74	49.77

Table S4. All the significantly upregulated DEGs in response to combinatorial treatment of starvation induced autophagy and cisplatin in ACHN cell line were generated by EdgeR package. Upto 86 upregulated DEGs were identified with log2Fc >2 with adjusted p-value <0.05.

Sl. No.	Gene_ID	Entrez_ID	Gene Annotation	Gene symbol	Log2Fold Change
**1**	ENSG00000100033	5625	proline dehydrogenase (oxidase) 1	PRODH	5.86
**2**	ENSG00000136925	158427	thiosulfate sulfurtransferase like domain containing 2	TSTD2	4.61
**3**	ENSG00000158828	65018	PTEN induced putative kinase 1	PINK1	3.98
**4**	ENSG00000132906	842	caspase 9	CASP9	3.93
**5**	ENSG00000136997	4609	v-myc avian myelocytomatosis viral oncogene homolog	MYC	3.89
**6**	ENSG00000126581	8678	beclin 1	BECN1	3.88
**7**	ENSG00000141510	7157	tumor protein p53	TP53	3.85
**8**	ENSG00000172115	54205	cytochrome c	CYCS	3.83
**9**	ENSG00000173530	8793	TNF receptor superfamily member 10d	TNFRSF10D	3.81
**10**	ENSG00000168906	4144	methionine adenosyltransferase 2A	MAT2A	3.80
**11**	ENSG00000140941	81631	microtubule-associated protein 1 light chain 3 beta	MAP1LC3B	3.78

**12**	ENSG00000197769	440738	microtubule associated protein 1 light chain 3 gamma	MAP1LC3C	3.76
**13**	ENSG00000237296	641298	SMG1P1, nonsense mediated mRNA decay associated PI3K related kinase pseudogene 1	SMG1P1	3.75
**14**	ENSG00000164305	836	caspase 3	CASP3	3.73
**15**	ENSG00000172977	10524	lysine acetyltransferase 5	KAT5	3.72
**16**	ENSG00000109971	3312	heat shock protein family A	HSPA8	3.70
**17**	ENSG00000136231	10643	insulin like growth factor 2 mRNA binding protein 3	IGF2BP3	3.67
**18**	ENSG00000139618	675	BRCA2, DNA repair associated	BRCA2	3.67
**19**	ENSG00000132356	5562	protein kinase AMP-activated catalytic subunit alpha 1	AMPK	3.65
**20**	ENSG00000071794	6596	Helicase-like transcription factor	HLTF	3.63
**21**	ENSG00000178252	11180	WD repeat domain 6	WDR6	3.61
**22**	ENSG00000176986	9632	SEC24 family member C	SEC24C	3.58
**23**	ENSG00000164654	54468	meiosis regulator for oocyte development	MIOS	3.55
**24**	ENSG00000213621	642641	Ribosomal protein SA pseudogene 54	RPSAP54	3.48
**25**	ENSG00000111725	5564	protein kinase AMP-activated non-catalytic subunit beta 1	PRKAB1	3.42
**26**	ENSG00000146834	56257	Methylphosphate capping enzyme	MEPCE	3.39
**27**	ENSG00000139112	23710	GABA type A receptor associated protein like 1	GABARAPL1	3.31
**28**	ENSG00000149547	9538	EI24 autophagy associated transmembrane protein	EI24	3.29
**29**	ENSG00000253537	56108	protocadherin gamma subfamily A, 7	PCDHGA7	3.28
**30**	ENSG00000075239	38	acetyl-CoA acetyltransferase 1	ACAT1	3.25

**31**	ENSG00000117862	51060	Thioredoxin domain containing 12	TXNDC12	3.23
**32**	ENSG00000100644	3091	hypoxia inducible factor 1 alpha subunit	HiF1A	3.01
**33**	ENSG00000137496	10068	interleukin 18 binding protein	IL18BP	3.01
**34**	ENSG00000184557	9021	suppressor of cytokine signaling 3	SOCS3	2.95
**35**	ENSG00000116044	4780	nuclear factor, erythroid 2 like 2	NFE2L2	2.93
**36**	ENSG00000243547	644063	Heterogeneous nuclear ribonucleoprotein K pseudogene 4	HNRNPKP4	2.92
**37**	ENSG00000104419	10397	N-myc downstream regulated 1	NDRG1	2.90
**38**	ENSG00000004700	5965	RecQ like helicase	RECQL	2.90
**39**	ENSG00000165943	64112	modulator of apoptosis 1	MOAP1	2.89
**40**	ENSG00000005194	57019	cytokine induced apoptosis inhibitor 1	CIAPIN1	2.85
**41**	ENSG00000170345	2353	FosB proto-oncogene, AP-1 transcription factor subunit	FOS	2.84
**42**	ENSG00000159388	7832	BTG anti-proliferation factor 2	BTG2	2.83
**43**	ENSG00000076685	22978	5,-nucleotidase, cytosolic II	NT5C2	2.80
**44**	ENSG00000106100	10392	nucleotide binding oligomerization domain containing 1	NOD1	2.78
**45**	ENSG00000163399	476	ATPase, Na+/K+ transporting, alpha 1 polypeptide	ATP1A1	2.76
**46**	ENSG00000172071	9451	eukaryotic translation initiation factor 2 alpha kinase 3	EIF2AK3	2.71
**47**	ENSG00000075975	23609	Makorin ring finger protein 2	MKRN2	2.68
**48**	ENSG00000123416	10376	Tubulin, alpha 1b	TUBA1B	2.68
**49**	ENSG00000175344	1139	cholinergic receptor nicotinic alpha 7 subunit	CHRNA7	2.67
**50**	ENSG00000175104	7189	TNF receptor associated factor 6	TRAF6	2.66
**51**	ENSG00000269713	400818	neuroblastoma breakpoint family	NBPF9	2.65

			member 9		
**52**	ENSG00000163874	80149	zinc finger CCCH-type containing 12A	ZC3H12A	2.65
**53**	ENSG00000198146	54989	Zinc finger protein 770	ZNF770	2.63
**54**	ENSG00000019995	54764	Zinc finger, RAN-binding domain containing 1	ZRANB1	2.63
**55**	ENSG00000118515	6446	serum/glucocorticoid regulated kinase 1	SGK1	2.62
**56**	ENSG00000116954	64121	Ras related GTP binding C	RRAGC	2.61
**57**	ENSG00000073111	4171	Minichromosome maintenance complex component 2	MCM2	2.6
**58**	ENSG00000172530	54971	BTG3 associated nuclear protein	BANP	2.6
**59**	ENSG00000085491	29957	solute carrier family 25 member 24	SLC25A24	2.59
**60**	ENSG00000056558	7185	TNF receptor associated factor 1	TRAF1	2.55
**61**	ENSG00000142178	150094	salt inducible kinase 1	SIK1	2.51
**62**	ENSG00000141376	54828	BCAS3, microtubule associated cell migration factor	BCAS3	2.51
**63**	ENSG00000100462	10419	protein arginine methyltransferase 5	PRMT5	2.5
**64**	ENSG00000144560	9686	vestigial like family member 4	VGLL4	2.49
**65**	ENSG00000101266	1457	casein kinase 2 alpha 1	CSNK2A1	2.49
**66**	ENSG00000153094	10018	BCL2 like 11	BCL2L11	2.49
**67**	ENSG00000100129	51386	Eukaryotic translation initiation factor 3, subunit L	EIF3L	2.48
**68**	ENSG00000154803	201163	folliculin	FLCN	2.48
**69**	ENSG00000132155	5894	V-raf-1 murine leukemia viral oncogene homolog 1	RAF1	2.48
**70**	ENSG00000128016	7538	ZFP36 ring finger protein	ZFP36	2.47
**71**	ENSG00000105355	10226	Perilipin 3	PLIN3	2.46
**72**	ENSG00000069869	4734	neural precursor cell expressed, developmentally down-regulated 4, E3 ubiquitin protein	NEDD4	2.46

			ligase		
**73**	ENSG00000148339	114789	solute carrier family 25 member 25	SLC25A25	2.43
**74**	ENSG00000183098	10082	glypican 6	GPC6	2.42
**75**	ENSG00000068903	22933	sirtuin 2	SIRT2	2.42
**76**	ENSG00000124762	1026	cyclin dependent kinase inhibitor 1A	CDKN1A	2.41
**77**	ENSG00000092871	117584	ring finger and FYVE like domain containing E3 ubiquitin protein ligase	RFFL	2.39
**78**	ENSG00000135720	1783	Dynein, cytoplasmic 1, light intermediate chain 2	DYNC1LI2	2.38
**79**	ENSG00000131791	5565	protein kinase AMP-activated non-catalytic subunit beta 2	PRKAB2	2.36
**80**	ENSG00000264364	140735	dynein light chain LC8-type 2	DYNLL2	2.33
**81**	ENSG00000237506	220885	Ribosomal protein SA pseudogene 15	RPSAP15	2.31
**82**	ENSG00000131558	60412	exocyst complex component 4	EXOC4	2.27
**83**	ENSG00000182473	23265	exocyst complex component 7	EXOC7	2.11
**84**	ENSG00000177169	8408	unc-51 like autophagy activating kinase 1	ULK1	2.07
**85**	ENSG00000116285	54206	ERBB receptor feedback inhibitor 1	ERRFI1	2.07
**86**	ENSG00000165861	53349	zinc finger FYVE-type containing 1	ZFYVE1	2.01

Table S5. All the significantly downregulated DEGs in response to combinatorial treatment of starvation induced autophagy and cisplatin in ACHN cell line. Top 60 DEGs were identified by EdgeR with log2Fc >2 with adjusted p-value <0.05.

Sl. No.	Gene_ID	Entrez_ID	Gene Annotation	Gene symbol	Log2Fold Change
**1**	ENSG00000254960	100874251	KIRREL3 antisense RNA 2	KIRREL3-AS2	-5.11
**2**	ENSG00000118971	894	cyclin D2	CCND2	-3.98
**3**	ENSG00000116039	525	ATPase H+ transporting V1 subunit B1	ATP6V1B1	-3.95

**4**	ENSG00000198695	4541	Mitochondrially encoded NADH dehydrogenase 6	ND6	-3.88
**5**	ENSG00000198763	4536	Mitochondrially encoded NADH dehydrogenase 2	ND2	-3.86
**6**	ENSG00000245573	497258	BDNF antisense RNA	BDNF-AS	-3.84
**7**	ENSG00000015479	9782	matrin 3	MATR3	-3.44
**8**	ENSG00000181722	26137	zinc finger and BTB domain containing 20	ZBTB20	-3.42
**9**	ENSG00000146955	401409	RAB19, member RAS oncogene family	RAB19	-3.41
**10**	ENSG00000198886	4538	Mitochondrially encoded NADH dehydrogenase 4	ND4	-3.38
**11**	ENSG00000092853	63967	claspin	CLSPN	-3.38
**12**	ENSG00000114861	27086	forkhead box P1	FOXP1	-3.32
**13**	ENSG00000235034	342918	chromosome 19 open reading frame 81	C19orf81	-3.31
**14**	ENSG00000198625	4194	MDM4, p53 regulator	MDM4	-3.31
**15**	ENSG00000198786	4540	Mitochondrially encoded NADH dehydrogenase 5	ND5	-3.3
**16**	ENSG00000102524	10673	tumor necrosis factor superfamily member 13b	TNFSF13B	-3.28
**17**	ENSG00000130748	54958	transmembrane protein 160	TMEM160	-3.27
**18**	ENSG00000198712	4513	Mitochondrially encoded cytochrome c oxidase II	COX2	-3.27
**19**	ENSG00000157613	90993	cAMP responsive element binding protein 3 like 1	CREB3L1	-3.27
**20**	ENSG00000243449	401115	chromosome 4 open reading frame 48	C4orf48	-3.26
**21**	ENSG00000078081	27074	lysosomal associated membrane protein 3	LAMP3	-3.26
**22**	ENSG00000182154	64975	mitochondrial ribosomal protein L41	MRPL41	-3.23
**23**	ENSG00000260001	100507588	transforming growth factor beta receptor 3 like	TGFBR3L	-3.22
**24**	ENSG00000101109	6789	serine/threonine kinase 4	STK4	-3.05
**25**	ENSG00000198899	4508	Mitochondrially	ATP6	-3.04

			encoded ATP synthase 6		
**26**	ENSG00000161642	25946	zinc finger protein 385A	ZNF385A	-3.01
**27**	ENSG00000237973	107075141	Hsa-mir-6723	MTCO1P12	-3
**28**	ENSG00000112137	221692	phosphatase and actin regulator 1	PHACTR1	-3
**29**	ENSG00000225630	100652939	MT-ND2 pseudogene 28	MTND2P28	-2.99
**30**	ENSG00000248527	44575	Mitochondrially encoded ATP synthase 6 pseudogene 1	MTATP6P1	-2.99
**31**	ENSG00000106211	3315	heat shock protein family B	HSPB1	-2.99
**32**	ENSG00000198840	4537	Mitochondrially encoded NADH dehydrogenase 3	ND3	-2.94
**33**	ENSG00000060339	55749	cell division cycle and apoptosis regulator 1	CCAR1	-2.94
**34**	ENSG00000254206	728888	nuclear pore complex interacting protein family member B11	NPIPB11	-2.91
**35**	ENSG00000227507	4050	lymphotoxin beta	LTB	-2.91
**36**	ENSG00000213639	5500	protein phosphatase 1 catalytic subunit beta	PPP1CB	-2.91
**37**	ENSG00000251562	378938	metastasis associated lung adenocarcinoma transcript 1	MALAT1	-2.9
**38**	ENSG00000051523	1535	cytochrome b-245 alpha chain	CYBA	-2.77
**39**	ENSG00000198727	4519	Mitochondrially encoded cytochrome b	CYTB	-2.76
**40**	ENSG00000149639	140710	suppressor of glucose, autophagy associated 1	SOGA1	-2.7
**41**	ENSG00000242299	100131863	Ribosomal Protein S18 Pseudogene 5	RPS18P5	-2.69
**42**	ENSG00000110395	867	Cbl proto-oncogene	CBL	-2.64
**43**	ENSG00000142544	90353	cytosolic thiouridylase subunit 1	CTU1	-2.62
**44**	ENSG00000111057	3875	keratin 18	KRT18	-2.59
**45**	ENSG00000175567	7351	uncoupling protein 2	UCP2	-2.52
**46**	ENSG00000168092	5049	platelet activating factor acetylhydrolase 1b catalytic subunit 2	PAFAH1B2	-2.5
**47**	ENSG00000180211	646949	Ribosomal Protein L23 Pseudogene 6	RPL23P6	-2.49

**48**	ENSG00000249992	25907	transmembrane protein 158	TMEM158	-2.49
**49**	ENSG00000236824	618	brain cytoplasmic RNA 1	BCYRN1	-2.49
**50**	ENSG00000130222	10912	growth arrest and DNA damage inducible gamma	GADD45G	-2.49
**51**	ENSG00000164761	4982	TNF receptor superfamily member 11b	TNFRSF11B	-2.49
**52**	ENSG00000228253	4509	Mitochondrially encoded ATP synthase 8	ATP8	-2.48
**53**	ENSG00000103490	29108	PYD and CARD domain containing	PYCARD	-2.47
**54**	ENSG00000234009	388907	Ribosomal protein L5 pseudogene 34	RPL5P34	-2.46
**55**	ENSG00000087088	581	BCL2 associated X, apoptosis regulator	BAX	-2.42
**56**	ENSG00000159079	56683	ATPase H+ transporting V1 subunit B1	CFAP298	-2.4
**57**	ENSG00000156735	9530	BCL2 associated athanogene 4	BAG4	-2.4
**58**	ENSG00000101745	23253	cyclin D2	ANKRD12	-2.35
**59**	ENSG00000075151	8672	eukaryotic translation initiation factor 4 gamma 3	EIF4G3	-2.35
**60**	ENSG00000080824	3320	heat shock protein 90 alpha family class A member 1	HSP90AA1	-2.02

Table S6. Top ranked hub genes with individual scores determined by 12 different algorithm run by CytoScape plugin Cytohubba.

**Betweeness**	**Bottleneck**	**Closeness**	**Clustering coefficient**	**Degree**	**DMNC**	**EcCentricity**	**EPC**	**MCC**	**MNC**	**Radiality**	**Stress**
TP53 (238.24)	TP53 (14)	TP53 (26.5)	ZFYVE1 (1)	TP53 (24)	EIF2AK3 (0.89)	CASP3 (0.5)	TP53 (14.15)	TP53 (1453719)	TP53 (23)	TP53 (4)	TP53 (634)
MYC (92.48)	MYC (4)	MYC (23.83)	NDRG1 (1)	MYC (19)	NFE2L2 (0.87)	TP53 (0.5)	BECN1 (13.46)	CASP3 (1453202)	MYC (19)	MYC (3.79)	MYC (296)
MAP1LC3B (70.86)	MAP1LC3B (3)	MAP1LC3B (23.33)	BECN1 (1)	MAP1LC3B (18)	CASP9 (0.84)	MAP1LC3B (0.33)	MYC (13.46)	MAP1LC3B (1452894)	MAP1LC3B (18)	MAP1LC3B (3.75)	MAP1LC3B (294)
BECN1 (60.65)	GABARAPL1 (3)	HIF1A (23.33)	ZFP36 (1)	HIF1A (18)	BCL2L11 (0.83)	NFE2L2 (0.33)	HIF1A (13.36)	HIF1A (1452822)	HIF1A (18)	HIF1A (3.75)	BECN1 (266)
TRAF1 (56)	CASP3 (3)	BECN1 (23.33)	NFE2L2 (0.97)	BECN1 (18)	CYCS (0.79)	HIF1A (0.33)	CASP3 (13.25)	MYC (1452806)	BECN1 (18)	BECN1 (3.75)	HIF1A (204)
CASP3 (51.10)	SOCS3 (2)	CASP3 (22.5)	EIF2AK3 (0.96)	CASP3 (16)	CDKN1A (0.76)	RAF1 (0.33)	MAP1LC3B (13.20)	CYCS (1452240)	CASP3 (16)	CASP3 (3.72)	CASP3 (202)
HIF1A (45.07)	TRAF1 (2)	CYCS (20.83)	MAP1LC3C (0.93)	CYCS (13)	MAP1LC3C (0.66)	NEDD4 (0.33)	CYCS (12.34)	CASP9 (1452240)	CYCS (13)	CYCS (3.58)	ULK1 (134)
ULK1 (27.91)	PRKAA1 (2)	CDKN1A (20.33)	BCL2L11 (0.89)	CDKN1A (12)	RAF1 (0.65)	MYC (0.33)	CASP9 (12.10)	EIF2AK3 (1451520)	CDKN1A (12)	CDKN1A (3.55)	TRAF1 (130)
NEDD4 (15.60)	BECN1 (2)	CASP9 (20.33)	CASP9 (0.87)	ULK1 (12)	CASP3 (0.65)	KAT5 (0.33)	BCL2L11 (12.02)	BECN1 (727152)	ULK1 (12)	CASP9 (3.55)	NEDD4 (86)
SOCS3 (12.52)	MAP1LC3C (1)	ULK1 (20.16)	RAF1 (0.85)	CASP9 (12)	ZFYVE1 (0.64)	CDKN1A (0.33)	CDKN1A (12.00)	BCL2L11 (726480)	CASP9 (12)	EIF2AK3 (3.51)	GABARAPL1 (62)

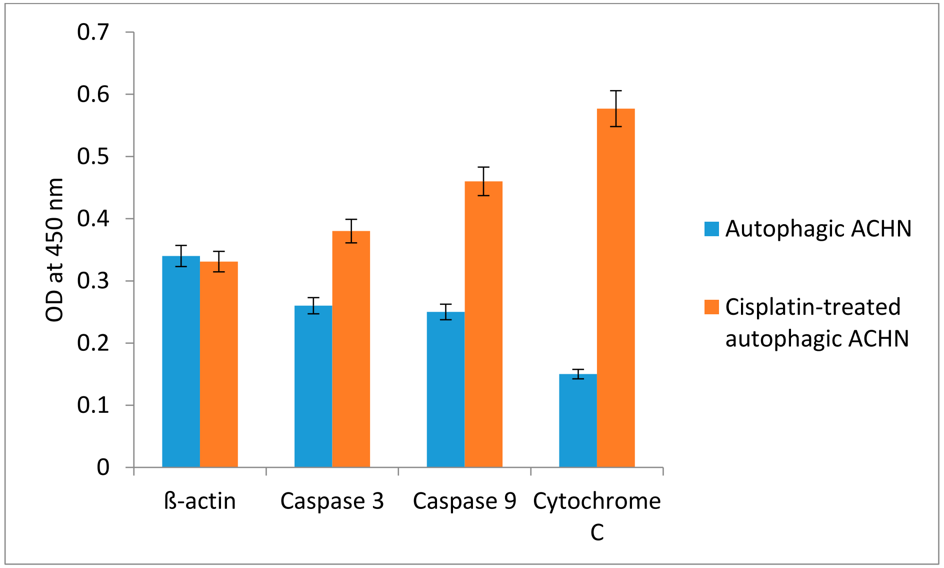

Figure S21. Antibody based indirect ELISA was used to assess apoptosis in autophagic ACHN cells and Cisplatin-treated autophagic ACHN cells. The cytosolic protein was extracted from each treatment condition and quantification of apoptosis-related biomarkers were achieved by recording absorbance at 450 nm using SPECTROStar Nano plate reader (BMG Labteck, Germany).
